# A detailed molecular picture of protein folding during active translation

**DOI:** 10.64898/2026.07.03.736445

**Published:** 2026-07-04

**Authors:** Amir Bitran, Carlos Bustamante, Susan Marqusee

**Affiliations:** 1 Department of Molecular and Cell Biology, University of California, Berkeley, CA, USA; 2 California Institute for Quantitative Biosciences, University of California, Berkeley, CA, USA; 3 Department of Chemistry, University of California, Berkeley, CA, USA; 4 Jason Choy Laboratory of Single Molecule Biophysics, University of California, Berkeley, CA, USA; 5 Howard Hughes Medical Institute, University of California, Berkeley, CA, USA; 6 Department of Physics, University of California, Berkeley, CA, USA; 7 Molecular Biophysics and Integrative Bioimaging Division, Lawrence Berkeley National Laboratory, Berkeley, CA, USA; 8 Kavli Energy Nanoscience Institute, University of California, Berkeley, CA, USA; 9 Chan Zuckerberg Biohub–San Francisco, San Francisco, CA, USA

## Abstract

All proteins can begin to fold on the ribosome, and many proteins critically rely on co- translational folding to attain their native conformation. The molecular details underlying this crucial process, however, remain largely unknown and are not accounted for by structure predictions such as AlphaFold. To probe high-resolution folding during active translation, we develop a novel application of hydrogen-deuterium pulse labeling. We show that two proteins sequentially adopt stable structure during elongation, while a third protein only has time to loosely fold during active elongation. This loose folding kinetically traps the N-terminus and alters the post-translational folding pathway, allowing it to circumvent an aggregation-prone intermediate. These results highlight the crucial non-equilibrium coupling between translation and folding and reveal diverse strategies to promote robust co-translational folding.

Despite remarkable advances in our ability to predict the three-dimensional structure of proteins from their amino acid sequences (1,2), the specific molecular mechanisms ensuring robust and efficient protein folding, and their associated breakdown in age-related misfolding diseases, remain enigmatic (3,4). Protein folding in the cell often begins on the ribosome, where a nascent polypeptide chain experiences a dynamically evolving energy landscape as elongation proceeds and amino acids are added to its C terminus. Indeed, co-translational folding has been shown to improve the folding yield of proteins that do not efficiently refold from denaturant (5-10). This process is likely broadly beneficial given the appreciable fraction of proteins across proteomes that cannot fold autonomously (11-14). These early co-translational folding events further initiate nascent-chain proteostasis by recruiting a stereotyped sequence of chaperone interactions (15-21) and by nucleating interactions with quaternary assembly partners (10,22-26) at precise points in translation. Local variations in translation elongation rate have evolved to orchestrate these processes, as evidenced by conserved rare codons at putative folding intermediates (27-31) and enhanced ribosome pausing events correlated with chaperone recruitment (15,32,33).

Despite its critical importance, we do not yet understand the detailed molecular mechanisms underlying co-translational protein folding. Recent elegant studies have elucidated conformational features of this time-resolved folding process using one-dimensional order parameters such as global extension (34,35) and intramolecular FRET or PET (36-39), but a high-resolution description of the process is lacking. This is in stark contrast to refolding from denaturant, where the folding mechanism can be probed at near residue-level resolution using pulse-labeling hydrogen deuterium exchange (HDX) coupled to mass spectrometry (MS) or NMR (40-44). Recent HDX-MS studies have been carried out on stalled ribosomal nascent chain (RNC) providing an intricate structural and dynamic characterization of RNC complexes at equilibrium including the detailed effect of chaperone interactions (10,17-19). But co-translational folding is intrinsically a non-equilibrium process, and thus attaining a complete picture of the process necessitates probing time-resolved folding during elongation. This requires solving two major technical hurdles:1) deconvoluting the non-equilibrium kinetic coupling between active elongation and folding, and 2) The ability to detect, by mass spectrometry, the nascent chain deuteration (or the analogous structural readout in other biophysical techniques) amidst the complex background of ribosomal proteins, elongation factors, and nucleic acid components involved in the reaction. Here, we address these challenges by developing a novel application of pulse-labeling HDX-MS and provide a high-resolution time-resolved description of the co-translational folding pathways of three proteins from distinct families. These studies reveal a diversity of behaviors governed by both nascent chain folding propensities and the kinetic coupling between folding and translation elongation. We further reveal how non-equilibrium folding on the ribosome can introduce long-lasting hysteresis into the folding pathway that ultimately affects the folding yield.

## Monitoring time-resolved protein folding during active translation by HDX-MS

**Figure 1:**
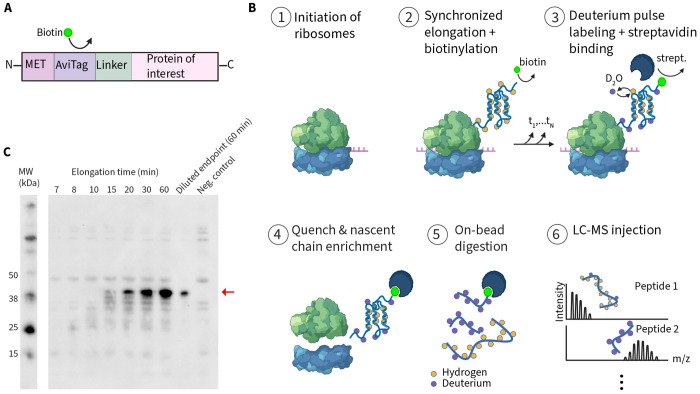
Experimental pipeline to monitor nascent chain (NC) folding during synchronized translation via hydrogen-deuterium exchange (HDX) (**A**) Map of constructs used for experiments. An N-terminal AviTag is biotinylated by the enzyme BirA (**B**) Summary of experimental design. Synchronized translation is achieved by pre-initiating ribosomes on mRNA (step 1) followed by addition of complete amino acid mix and pre-charged tRNA to begin elongation (step 2) coupled to nascent chain biotinylation. At various elongation times, an aliquot is simultaneously pulsed with deuterium to label exposed amides and bound to streptavidin beads (step 3). The HDX reaction is quenched via acidification, which also dissociates ribosomes while nascent chains (NCs) remain bound to beads. (step 4). NCs are digested into peptides via addition of pepsin to the beads (step 5), then analyzed for deuteration via liquid-chromatography mass spectrometry (step 6). For details see Materials and Methods. (**C**) Example anti-biotin western blot using streptavidin HRP to monitor progress of synchronized elongation of *E. coli* alpha Tryptophan synthase. Red arrow indicates full-length protein. The 60 min timepoint is diluted 5-fold in the penultimate lane, and the final lane shows a negative control translation reaction without genetic template, revealing bands resulting from non-specific streptavidin HRP binding.

To resolve the challenges associated with performing pulse-labeling HDX-MS on actively translating nascent chains, we developed an approach that creates a synchronized *in vitro* translation reaction using PURExpress^®^, an implementation of the PURE *in vitro* translation system (45), followed by pulse deuterium labeling at different translation times coupled to biochemical enrichment of nascent polypeptide chains for detection by mass spectrometry. This enrichment is achieved by translating a protein of interest containing an N-terminal AviTag (Fig. 1A), which is co-translationally biotinylated by the enzyme BirA; the biotinylated nascent protein can then be pulled down with streptavidin. We synchronize translation by pre-initiating ribosomes on the mRNA, followed by addition of complete amino-acid mix, pre-charged tRNAs, and aurintricarboxylic acid to promote single-turnover elongation (Fig. 1B, fig. S1A). This leads to gradual formation of longer polypeptide chains as monitored by gel electrophoresis (Fig. 1C), culminating in production of full-length protein, at an approximate elongation rate of 0.5 AA/sec, consistent with previously measured rates using the PURE system (9).

At various translation timepoints, we simultaneously perform pulse-labeling HDX and nascent-chain enrichment by diluting an aliquot of the translation reaction into an aliquot of streptavidin beads that have been pre-equilibrated in deuterated buffer. Binding of the translating protein to streptavidin beads does not disrupt the integrity of ribosomal nascent chains, nor globally alter the protein conformation (fig. S1B-E). Following a ten-second labeling and binding period, we quench both the exchange and translation reactions by diluting the sample into ice-cold, low-pH buffer. All translation components are then separated from bead-bound nascent chains, which are then digested into peptides. The level of deuteration in each peptide is then monitored by liquid-chromatography mass spectrometry (LC-MS). We confirm that this approach successfully detects the on-ribosome population during elongation (fig. S1F and S2, see also Supplementary Note 1) and yields high peptide coverage for all our proteins of interest (fig. S2B), allowing us to monitor protection from deuteration as a representation of the time-resolved folding at multiple regions in a protein during synchronized translation.

## Protein subdomains acquire stable structure during translation

**Figure 2:**
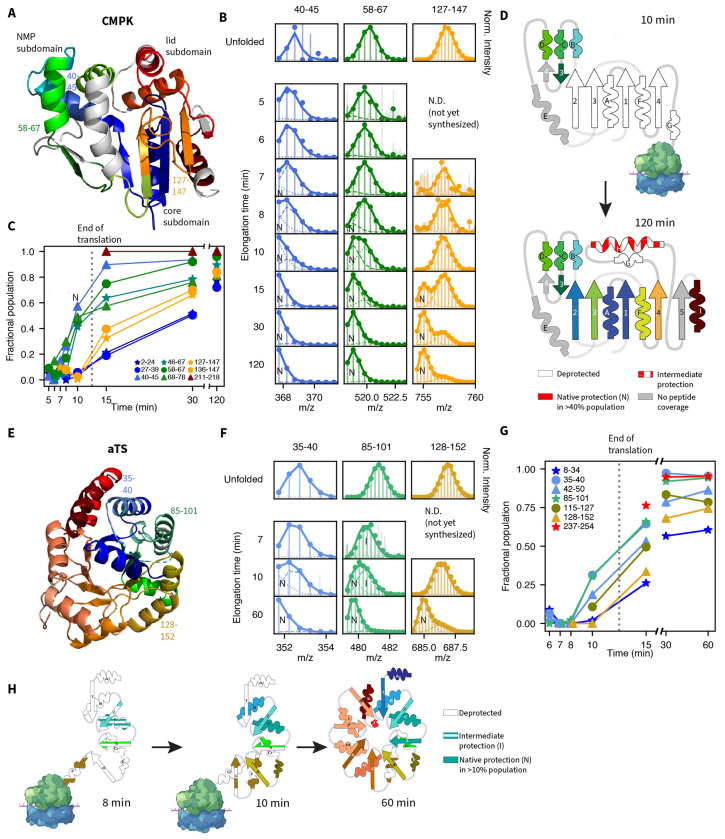
Partially translated subdomains begin folding during active translation **(A)** Structure of *E. coli* CMPK (PDB ID *2CMK*) with colored HDX-MS reporter peptides, three of which are labeled. **(B)** Mass spectra (translucent solid lines) vs. elongation time of example deuterated peptides, alongside maximally deuterated spectra (“Unfolded”). Round markers denote isotopic peaks, opaque solid lines indicate fits, and dashed lines show individual mode contributions (N=natively protected mode). **(C)** Fractional population associated with natively protected mode vs. elongation time for indicated peptides. **(D)** Schematic of CMPK co-translational folding. Structures spanned by peptides showing >40% natively protected population at the indicated times are colored solid, provided the native mode is ≤60% deuterated, while peptides showing ≥60% fractional population in a partially protected mode (<80% of maximal deuteration, see Materials and Methods) are shown in dashed colors. Peptides satisfying neither condition are shown in white, and gray denotes structures lacking peptide coverage. **(E)** Structure of *E. coli* aTS (PDB ID *1WQ5*) with color-coded reporter peptides. **(F)** Mass spectra for indicated aTS peptides as in (B) **(G)** Same as (C) for reporter peptides from aTS. **(H)** Same as (D) for aTS. In this case, solid colors refer to peptides with >10% natively protected population.

We applied this method to monitor the co-translational folding of various proteins that have been previously predicted to fold co-translationally, beginning with *E. coli* cytidine monophosphate kinase or CMPK (Fig. 2A), a protein from the P-loop structural family. All-atom simulations predict that the co-translational folding of CMPK begins with the lid subdomain after roughly 130 AAs have emerged from the ribosome (29) (fig. S3). We carried out our HDX-MS approach on a synchronized CMPK translation reaction and followed the mass spectra for multiple peptides (Fig. 2A, replicate reaction in fig. S4) spanning all three subdomains of this protein (spectra in Fig. 2B and fig. S4A).

To elucidate the CMPK folding trajectory, we globally fit the mass spectra associated with each peptide to multiple modes reflecting different levels of deuteration (Fig. 2B-C). For most CMPK peptides, two modes are required, a protected and a deprotected mode (Fig. 2D), as determined by a Bayesian information criterion approach (Materials and Methods, fig. S5). The pattern of deuteration for the protected mode correlates with the expected native secondary structure of CMPK (fig. S4D). Thus, as a proxy for native-like folding, we monitored the fractional population associated with this protected mode as a function of elongation time across all peptides (Fig. 2C and fig. S4). All peptides from the NMP subdomain show a concerted uptick in native protection after 8 mins of elongation, at which point a substantial fraction of ribosomes have translated >40 AA past the end of the subdomain (fig. S4B). This result strongly suggests that this subdomain folds co-translationally upon its emergence from the ribosome exit tunnel, which typically spans ~30-40 AA. In contrast, the core subdomain does not fold until after synthesis is complete (15 mins timepoint), consistent with the core beta-sheet topology involving long-range contacts between the N-and-C termini. We note that for some peptides, the protected fraction saturates at <100%, likely due to a small fraction of prematurely stalled ribosomes that do not complete synthesis (fig. S4B). Moreover, peptides from the lid subdomain show only partial protection even in the native state (fig. S4A-C), consistent with its known flexibility in structural homologs such as adenylate kinase (46). Thus, CMPK folds through a sequence of intermediates beginning with early co-translational folding of the N-terminal NMP subdomain, followed by the slower rearrangement of the core subdomain (Fig. 2D).

We next turned to the TIM barrel family, hypothesizing that we may observe sequential co-translational folding given that the N-terminal halves of some TIM barrels stably and rapidly fold in isolation (47,48). We performed pulse-labeling HDX-MS on a synchronized translation reaction of the well-characterized protein, *E. coli* alpha Tryptophan Synthase (aTS, Fig. 2E, replicate reaction in fig. S5). Indeed, we find that aTS folds co-translationally (Fig. 2F-H), beginning with early acquisition of partial protection of N-terminal structures, particularly the third alpha helix and beta sheet (peptide 85-101, Fig. 2F mode “I”). This is followed by an uptick in native protection from the second alpha helix through the fourth beta strand, and finally, post-translational protection of the C-terminal half barrel and the first beta strand, which must pair with the final strand to close the barrel (Fig. 2G-H). The co-translational folding intermediate seen here closely resembles an intermediate observed previously by HDX-MS upon refolding from denaturant (49) (fig. S6G), indicating that aTS folds via a similar mechanism on and off the ribosome.

## Translation alters the folding pathway of aggregation-prone HaloTag upon release from ribosome

**Figure 3:**
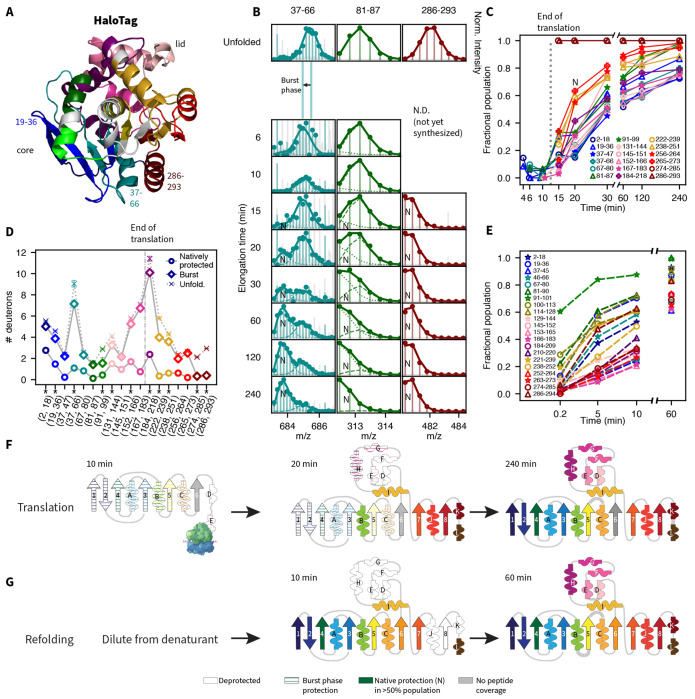
Translation biases HaloTag folding to circumvent aggregation-prone intermediate **(A)** Structure of HaloTag (PDB ID 5Y2Y) with peptides used in HDX-MS analysis colored and mapped onto the structure and subdomains indicated. **(B)** Deuterated peptide mass spectra vs. elongation time for indicated peptides alongside fully deuterated control (“Unfolded”) as in Fig. 2B, highlighting burst-phase protection for 37-66. **(C)** Native mode population vs. elongation time as in Fig. 2C. **(D)** Number of deuterons associated with natively protected mode (circles), burst-phase mode (diamonds) and unfolded state (Xs) for each peptide. Error bars represent 95% confidence intervals from bootstrapping and asterisks denote peptides exhibiting statistically significant burst-phase protection (Materials and Methods). Peptides to the right of the dashed line are only detected after 10 mins (post-translation). **(E)** Same as (C) for HaloTag refolding from 7.5M urea, with data re-analyzed from reference (42). **(F)** Schematic of HaloTag co-and-post translational folding with solid colors highlighting structures spanned by at least one peptide showing at least 50% natively protected fraction at the indicated times, striped colors indicating significant burst phase protection, white indicating no protection, and gray indicating no peptide coverage. **(G)** Same as (F) now depicting Halo Tag refolding from 7.5M urea.

The protein HaloTag (containing an α/β hydrolase fold) achieves a higher yield of natively folded protein when translated *in vitro* than upon refolding from a denatured state (9). To uncover the molecular basis underlying this observation, we performed pulse-labeling HDX-MS on a synchronized HaloTag translation reaction (Fig. 3, replicate reaction in fig. S7). As noted previously, we confirmed that HaloTag acquires its native structure upon *in vitro* translation by its ability to covalently bind the fluorescent, halogenated ligand TMR (fig. S7C) (9). Early during translation, several regions show subtle, albeit significant, protection relative to the unfolded state (Fig. 3D), indicating that HaloTag acquires a loosely folded, molten-globule like state while on the ribosome. However, for the entire protein, emergence of native-like protection is delayed until after translation is complete at which point folding occurs in three distinct stages beginning with the C-terminus of the core (Fig. 3B, C and F). This sequence of native-structure acquisition contrasts with HaloTag’s folding pathway upon dilution from denaturant (42) which proceeds through a distinct intermediate involving both the N- and C-terminal parts of the core (Fig. 3E and G). In addition, during refolding the C-terminal helices show a gradual rise in protection, in stark contrast to the near-instantaneous protection observed in these regions upon release from the ribosome. Finally, the refolding burst phase intermediate exhibits a distinct, generally less pronounced protection pattern than that seen in translation (fig. S8 C-D). These major differences are reproducible across biological replicates (fig. S7), and the delayed N-terminal folding relative to the C-terminus cannot be accounted for by lagging ribosomes (Supplementary Note 2). Thus, translation biases the subsequent folding pathway to proceed via intermediates different than that observed upon dilution from denaturant, which has been previously implicated in aggregation (9,42). This mechanism likely accounts for the observation that HaloTag achieves a higher folding yield upon translation than upon refolding off the ribosome.

## HaloTag real time co-translational folding lags behind equilibrium expectation

**Figure 4:**
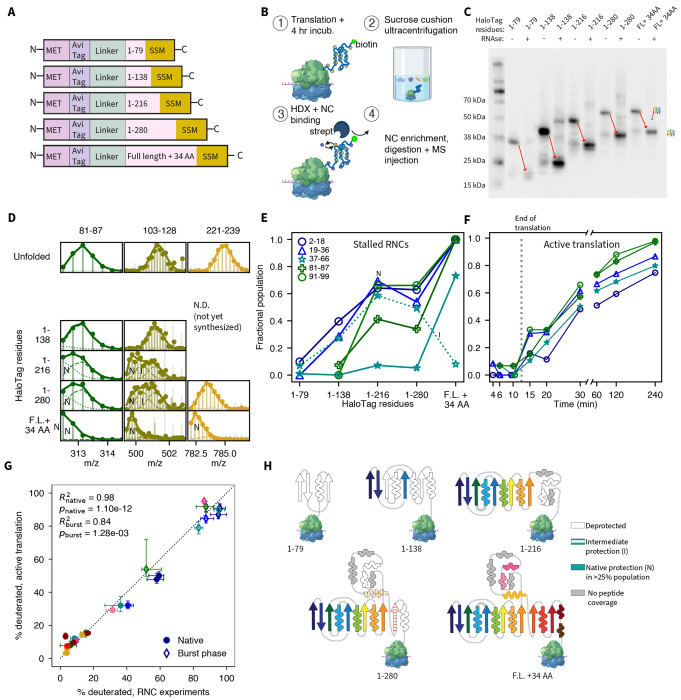
HaloTag nascent chains do not reach equilibrium during active elongation **(A)** Diagrams of constructs used for stalled ribosomal nascent chain (RNC) experiments including strong SecM (SSM) stalling sequence. **(B)** Experimental workflow for RNC preparation and analysis by HDX-MS. For details, see methods. **(C)** Streptavidin-HRP western blots showing RNAse-dependent migration shift in RNCs due to peptidyl tRNA attachment (red arrows). **(D)** Deuterated mass spectra for representative HaloTag peptides from equilibrated RNC constructs alongside maximally deuterated condition (“Unfolded”). **(E)** Fitted fractional populations associated with natively protected mode for indicated peptides from each RNC construct (opaque markers), and intermediate mode for peptide 37-66 (empty markers) **(F)** Same as Fig. 3C for subset of peptides shown in E. **(G)** Correlation between percentage deuteration (normalized to maximally deuterated control) in native (circles) and burst phase (diamonds) modes for each peptide in RNC and active translation experiments with indicated Pearson correlations. Error bars represent 95% confidence intervals from bootstrapping. **(H)** At each length, secondary structures are shown with solid colors if spanned by at least one peptide showing ≥25% natively protected population, with dashed colors if partially protected in HaloTag 1-280, in white if deprotected, and in gray if the region lacks peptide coverage.

HaloTag folds on a significantly slower timescale than that of translation itself (Fig. 3C), suggesting that nascent chains may not have time to reach conformational equilibrium during active elongation. To test this, we realized the equilibrium scenario by generating stalled ribosomal nascent chains (RNCs), which we incubated for four hours before isolation via sucrose cushion ultracentrifugation and then interrogated their folding status via pulse-labeling HDX as was done for actively translating NCs. These RNCs contained HaloTag N-terminal fragments of various lengths coupled to a strong SecM stalling sequence (50) and an N-terminal AviTag (Fig.4A-B). Our second-longest length, HaloTag 280, recapitulates the scenario where HaloTag is fully translated but not yet released from the ribosome, while our longest length (F.L. + 34 AA) contains an additional C-terminal linker to fully span the ribosomal exit tunnel, such that the native HaloTag sequence is fully emerged from the ribosome. We confirmed that our HDX pulse-labeling reports on RNCs that are stably attached to the ribosome (Fig. 4C) (51).

In contrast to actively-translating HaloTag which remains nearly deprotected on the ribosome (Fig. 3B), equilibrated RNCs show a length-dependent increase in protection across multiple structural regions (Fig. 4D and fig. S9). Various peptides show bi-or-trimodal mass spectra indicative of multiple populations that interconvert slowly relative to our labeling pulse duration (10 seconds). Thus, we quantify length-dependent protection by fitting all mass spectra to a combination of modes reflective of these populations, analogous to the analysis performed for actively translating NCs (Fig. 4E). At short lengths, N-terminal peptides show minimal, albeit significant protection relative to the unfolded state (Fig. 4D, E, and fig. S9C), resembling the burst-phase protection pattern in active elongation (Fig. 4G). However, at increasing RNC lengths, the N-terminus of the core populates increasingly protected states in a length-dependent manner (Fig. 4D,E and H), although this relationship with length is not perfectly monotonic, potentially due to complex stability effects related to distance from the ribosome (52-55). Interestingly, the regions that become protected closely match those protected in the denaturant refolding intermediate (Fig. 4H and 3G), suggesting a similar intermediate may form in equilibrated RNCs. Moreover, the native-mode protection in RNCs nearly perfectly correlates with that observed at the end of active translation (Fig. 4G), strongly suggesting the protected RNC intermediates are native-like. But crucially, while equilibrated RNCs show lengthdependent native protection of various core structures, these same regions do not show comparable protection during active translation until well after synthesis is complete (Fig. 4F). Thus, actively translating HaloTag remains out of equilibrium, as nascent chains do not have time to fold while on the ribosome despite their ability to do so.

## Discussion

**Figure 5:**
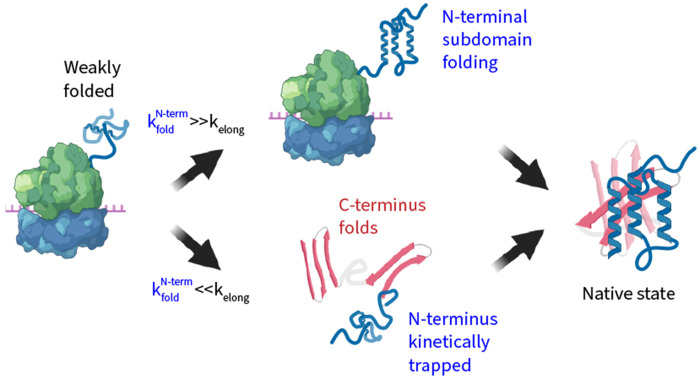
Nascent chain folding depends on kinetic competition between translation and folding rates. For details, see discussion

We have developed and implemented an HDX-MS approach that reveals the folding of a nascent polypeptide chain at high resolution during active translation. These studies provide significant new insights inaccessible from previous high-resolution structural studies of stalled, equilibrated nascent chains using HDX-MS (10,17-19) or NMR (52-54) or elegant time-resolved work following a single conformational probe at a time (34-39). The non-equilibrium nature of translation in our studies, coupled with the ability to follow folding across the entire chain, reveals how nascent chain folding is shaped by both thermodynamics and the kinetic coupling between translation and folding, leading to a diversity of co-translational behaviors. For CMPK and aTS (from the from the P-loop family and TIM barrel family, respectively) we observe that folding on the ribosome proceeds through a hierarchical series of intermediates involving N-terminal structural elements smaller than a complete domain (Fig. 5, top pathway). Although subdomain-level folding on the ribosome has long been postulated (55), including for TIM barrel proteins (56), our work provides direct experimental evidence of this process. For aTS, the co-translational intermediate we observe closely resembles an obligatory N-terminal refolding intermediate previously observed by HDX-MS (49), suggesting that, at least for some proteins, co-translational folding and folding from denaturant proceed through similar trajectories, consistent with previous findings (35,57). But for the large number of TIM barrels that do not effectively refold from denaturant (11,13,14), sequential co-translational folding as observed here may significantly simplify the acquisition of this topologically complex structure.

In contrast to the observations for aTS and CMPK, the protein HaloTag does not begin exhibiting native-like folding until after its release from the ribosome. Even though nascent HaloTag is capable of adopting a native-like intermediate on the ribosome, the timescale required for it to do so is significantly longer than the timescale of translation elongation. Instead, during active elongation, HaloTag nascent chains remain kinetically trapped in a a loosely folded, nonnative intermediate--reminiscent of that observed for the protein HemK (34-39)--characterized by subtle, albeit significant, protection at various N-terminal sequence regions (Fig. 3D). This protection is already observed at our earliest translation timepoints (Fig. 3B,D) and resembles that observed in our shortest RNCs (Fig. 4D, G), indicating this loosely folded intermediate forms early in translation. The kinetically-trapped N terminus does not have time to rearrange into a native-like structure before the emergence of the C-terminus, which instead, initiates native-like folding upon the protein’s release from the ribosome (Fig. 5, bottom pathway). In contrast, during dilution from denaturant, the initial presence of both the N and C terminus kinetically favors folding via an aggregation-prone intermediate involving contributions from both termini. Notably, this refolding intermediate resembles that formed by equilibrated stalled RNCs (Fig. 3G and 4H). Thus, the non-equilibrium folding delay during HaloTag translation is likely crucial in allowing the protein to avoid this problematic intermediate and attain a high folding yield. Our results with HaloTag underscore the importance of assays like the one developed here to monitor folding during active elongation, as folding behavior during non-equilibrium translation can differ starkly from that seen in equilibrated RNCs, as reported previously for calerythrin (35). Our HDX-MS approach can further reveal, at high resolution, alternative mechanisms by which translation benefits folding, such as allowing (sub)domains to fold sequentially to avoid post-translational misfolding (29,30,58).

It is important to note that the rate of translation elongation in PURE-based *in vitro* translation assays such as ours is significantly slower than the *in vivo* elongation rate in bacterial systems such as *E. coli*, from which PURE components are derived (~10-20 AA/sec). However, for the case of HaloTag, we expect that a faster rate of translation would only *further* heighten the discrepancy between equilibrium and real-time co-translational folding that we observe here. Moreover, for proteins that do fold via N-terminal intermediates, we expect co-translational folding to occur provided the associated folding rate is appreciably faster than translation. The N-terminal refolding intermediate populated by aTS, which closely resembles the co-translational intermediate observed here, forms in under a second (59) suggesting it may also form under a faster elongation rate. Closing the gap between the *in vitro* and *in vivo* elongation rates, for instance by performing translation in cell-based lysates or using homebuilt reconstituted systems with titratable components, will enable detailed investigations on the effects of the relative rates of translation. This includes the role of conserved rare codons such as those in the CMPK sequence shortly after the NMP-binding subdomain (27,29) which folds co-translationally in our assay.

In summary, our work reveals a diversity of strategies that are deployed by the ribosome to promote robust and efficient protein folding, from promoting sequential folding of subdomain elements to *delaying* folding to avoid problematic intermediates. Over the course of protein evolution, the fitness of a given variant may depend on its ability to exploit these folding mechanisms, and future work is needed to extend equilibrium biophysical fitness models (60-62) to account for non-equilibrium co-translational folding. Viral proteins, in particular, have likely evolved to exploit these factors given their documented reliance on cellular quality control mechanisms to fold robustly during infection (63). In the cell, co-translational folding efficiency is further influenced by ribosome-associated molecular chaperones and quaternary assembly partners that interact with nascent chains. In the future, the methodological platform presented here can be extended to account for these more complex mechanisms, as well as possible links between co-translational (mis)folding and disease. Finally, the principles presented here can be exploited to enhance the efficiency of *de novo* protein design and transgenic protein expression by optimizing for kinetic folding yield.

## Supplementary Material

Supplement 1

Materials and Methods

Supplementary Text

Figs. S1 to S9

Tables S1 to S4
