# A detailed molecular picture of protein folding during active translation

**DOI:** 10.64898/2026.07.03.736445

**Published:** 2026-08-10

**Authors:** Amir Bitran, Carlos Bustamante, Susan Marqusee

**Affiliations:** 1 Department of Molecular and Cell Biology, University of California, Berkeley, CA, USA.; 2 California Institute for Quantitative Biosciences, University of California, Berkeley, CA, USA.; 3 Department of Chemistry, University of California, Berkeley, CA, USA.; 4 Jason Choy Laboratory of Single Molecule Biophysics, University of California, Berkeley, CA, USA; 5 Howard Hughes Medical Institute, University of California, Berkeley, CA, USA; 6 Department of Physics, University of California, Berkeley, CA, USA; 7 Molecular Biophysics and Integrative Bioimaging Division, Lawrence Berkeley National Laboratory, Berkeley, CA, USA; 8 Kavli Energy Nanoscience Institute, University of California, Berkeley, CA, USA

## Abstract

All proteins can begin to fold on the ribosome, and many rely on co-translational folding to attain their native conformation. This process is not accounted for using structure prediction algorithms such as AlphaFold and its molecular details remain largely unknown. Here, we develop a hydrogen-deuterium pulse-labeling approach which reveals nascent polypeptide folding at a high level of structural detail and its kinetic coupling with translation. Two proteins exhibit hierarchical folding of structures smaller than a domain during translation. A third protein, however, does not have time to conformationally equilibrate on the ribosome, instead becoming kinetically trapped. This subsequently biases post-translational folding to avoid an aggregation-prone intermediate populated during refolding from denaturant. Our results reveal diverse strategies that promote robust protein folding during non-equilibrium translation.

The specific molecular mechanisms that ensure robust protein folding in the cell, and their associated breakdown in misfolding disease, remain enigmatic (1,2). These protein-folding dynamics are not accounted for by native-structure prediction tools such as AlphaFold (3,4). All proteins have the opportunity to begin folding during their biosynthesis on the ribosome, where a nascent polypeptide chain experiences a dynamically evolving energy landscape as elongation proceeds and amino acids are added to its C terminus. Indeed, this directional, non-equilibrium process of co-translational folding has been shown to be critical for ensuring robust folding of certain model proteins that do not efficiently refold once fully synthesized (5–10). These benefits of co-translational folding likely extend broadly given the appreciable fraction of proteins across proteomes that cannot fold autonomously (11–14). In addition, co-translational folding is directly linked to proteostasis, as nascent chains sequentially engage chaperones (15–21) and nucleate interactions with quaternary assembly partners (10,22–26) at precise points in translation. Local variations in translation elongation rate have evolved to orchestrate these processes, as evidenced by conserved rare codons at putative folding intermediates (27–32) and enhanced ribosome pausing events correlated with chaperone recruitment (15,33,34).

Despite its critical importance, we do not yet understand the detailed molecular mechanisms underlying co-translational protein folding. Recent experimental studies have succeeded in monitoring the compaction of a nascent chain during real-time elongation at high temporal resolution using optical tweezers(35,36) and intramolecular FRET or PET (37–40). But a high-resolution structural description of the process is still lacking. In contrast, refolding from denaturant can be probed at near residue-level resolution using pulse-labeling hydrogen deuterium exchange (HDX) coupled to mass spectrometry (MS) or NMR (41–45). Recent studies have managed to extend HDX-MS methodology to stalled ribosomal nascent chains (RNCs), providing an intricate structural and dynamic characterization of RNC complexes at equilibrium including the detailed effect of chaperone interactions (10,17–19). But co-translational folding is intrinsically a non-equilibrium process, and thus attaining a complete picture of the process necessitates probing time-resolved folding during elongation. This requires solving two major technical hurdles: 1) monitoring the kinetic coupling between active elongation and folding, and 2) detecting the nascent chain deuteration (or the analogous structural readout in other biophysical techniques) amidst the complex background of ribosomal proteins, elongation factors, and nucleic acid components involved in the reaction. Here, we address these challenges by developing a novel application of pulse-labeling HDX-MS and provide a high-resolution time-resolved description of the co-translational folding pathways of three proteins from distinct structural families. These studies reveal a diversity of behaviors governed by both nascent chain folding propensities and the kinetic coupling between folding and translation elongation. We further reveal how non-equilibrium folding on the ribosome can introduce long-lasting hysteresis into the folding pathway that ultimately affects the folding yield.

## Monitoring time-resolved protein folding during active translation by HDX-MS

**Figure 1:**
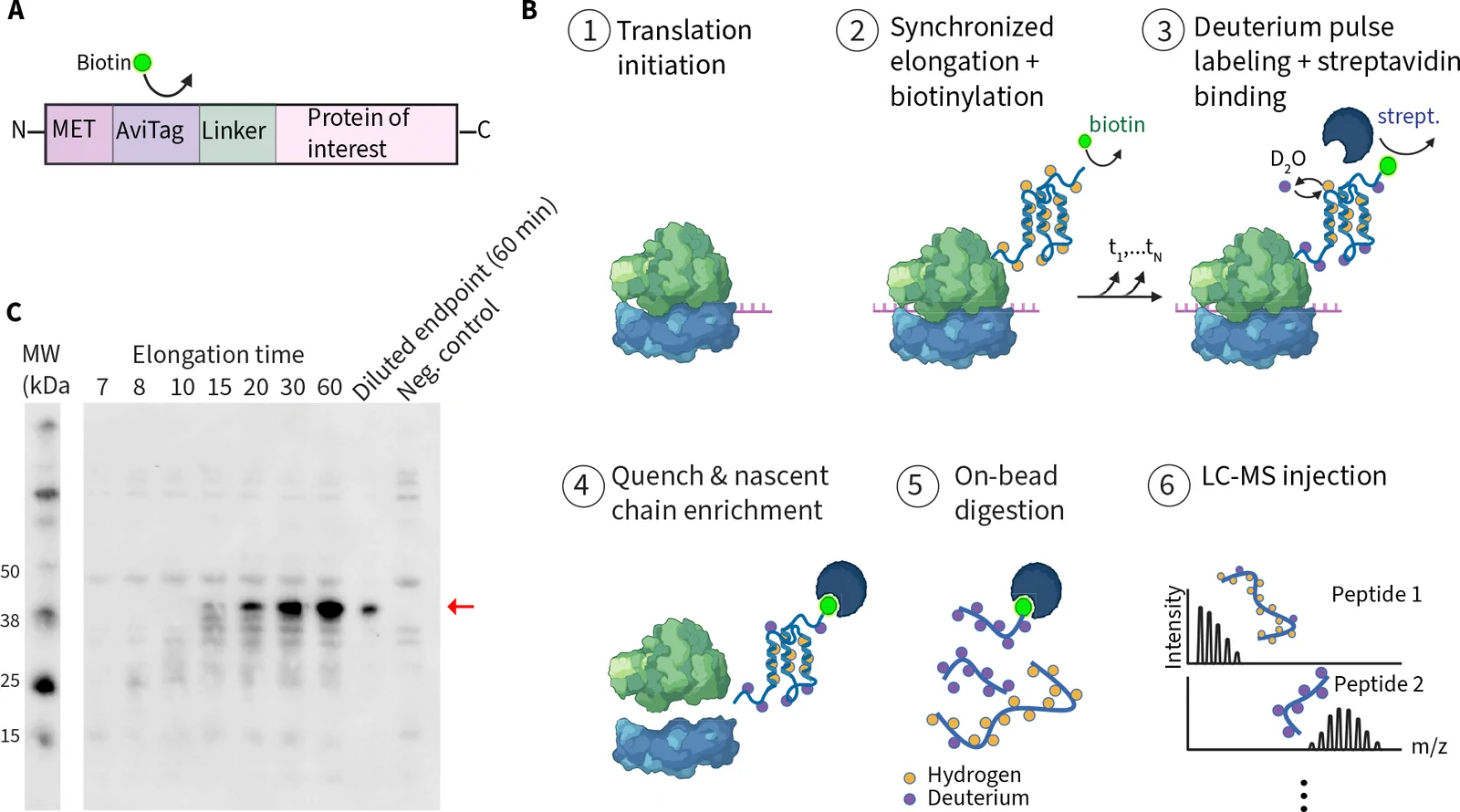
Experimental pipeline to monitor nascent chain (NC) folding during synchronized translation via hydrogen-deuterium exchange (HDX) **(A)** Map of constructs used for experiments. An N-terminal AviTag is biotinylated by the enzyme BirA. **(B)** Summary of experimental design. Synchronized translation is achieved by pre-initiating ribosomes on mRNA (step 1), followed by addition of complete amino acid mix and pre-charged tRNA to begin elongation (step 2), coupled to nascent-chain biotinylation. At various elongation times, an aliquot is pulsed with deuterium to label exposed amides and simultaneously bound to streptavidin beads (step 3). The HDX reaction is quenched via acidification, which also dissociates ribosomes while nascent chains (NCs) remain bound to beads (step 4). NCs are digested into peptides via addition of pepsin to the beads (step 5), then analyzed for deuteration via liquid-chromatography mass spectrometry (step 6). For details, see Materials and Methods. **(C)** Example anti-biotin western blot using streptavidin HRP to monitor progress of synchronized elongation of *E. coli* alpha Tryptophan synthase. Red arrow indicates full-length protein. The 60 min timepoint is diluted 5-fold in the penultimate lane, and the final lane shows a negative control translation reaction without genetic template, revealing bands resulting from non-specific streptavidin HRP binding.

To resolve the challenges associated with probing folding during active translation, we developed an approach to perform synchronized, single-turnover *in* vitro translation using the using PURExpress^®^ system (46) (Fig. 1A-B, fig. S1A). At various translation times, we pulse-label an aliquot of the translation reaction with deuterium. This allows exposed amides along the polypeptide backbone to exchange their protons for deuterons, while buried or stably hydrogen-bonded amides remain protonated, creating a chemical “snapshot” of solvent accessibility. Crucially, our hydrogen-deuterium exchange (HDX) reaction is coupled to biochemical enrichment of the nascent chain. To achieve this, we translate the protein of interest with an N-terminal AviTag (Fig. 1A), which is co-translationally biotinylated by the enzyme BirA. Then, during the pulse-labeling reaction, the biotinylated nascent protein is pulled down with streptavidin beads that have been pre-equilibrated in deuterated buffer. Bead binding does not disrupt RNC integrity, nor globally alter the protein conformation (fig. S1B-E). Following a ten-second labeling and binding period, we quench both the exchange and translation reactions by diluting the sample into ice-cold, low-pH buffer. All translation components are then separated from bead-bound nascent chains, and the nascent chains are then digested with protease, releasing the peptides from the beads. The peptides are then subjected to liquid-chromatography mass spectrometry (LC-MS) for detection of the amount of deuteration in the different regions in the nascent chain. This workflow successfully monitors the on-ribosome population during elongation (fig. S1F and S2, see also Supplementary Note 1) and yields high peptide coverage for all our proteins of interest (fig. S2B), indicating that we can monitor time-resolved folding at multiple regions in a protein during synchronized translation.

## Protein subdomains acquire stable structure during translation

**Figure 2:**
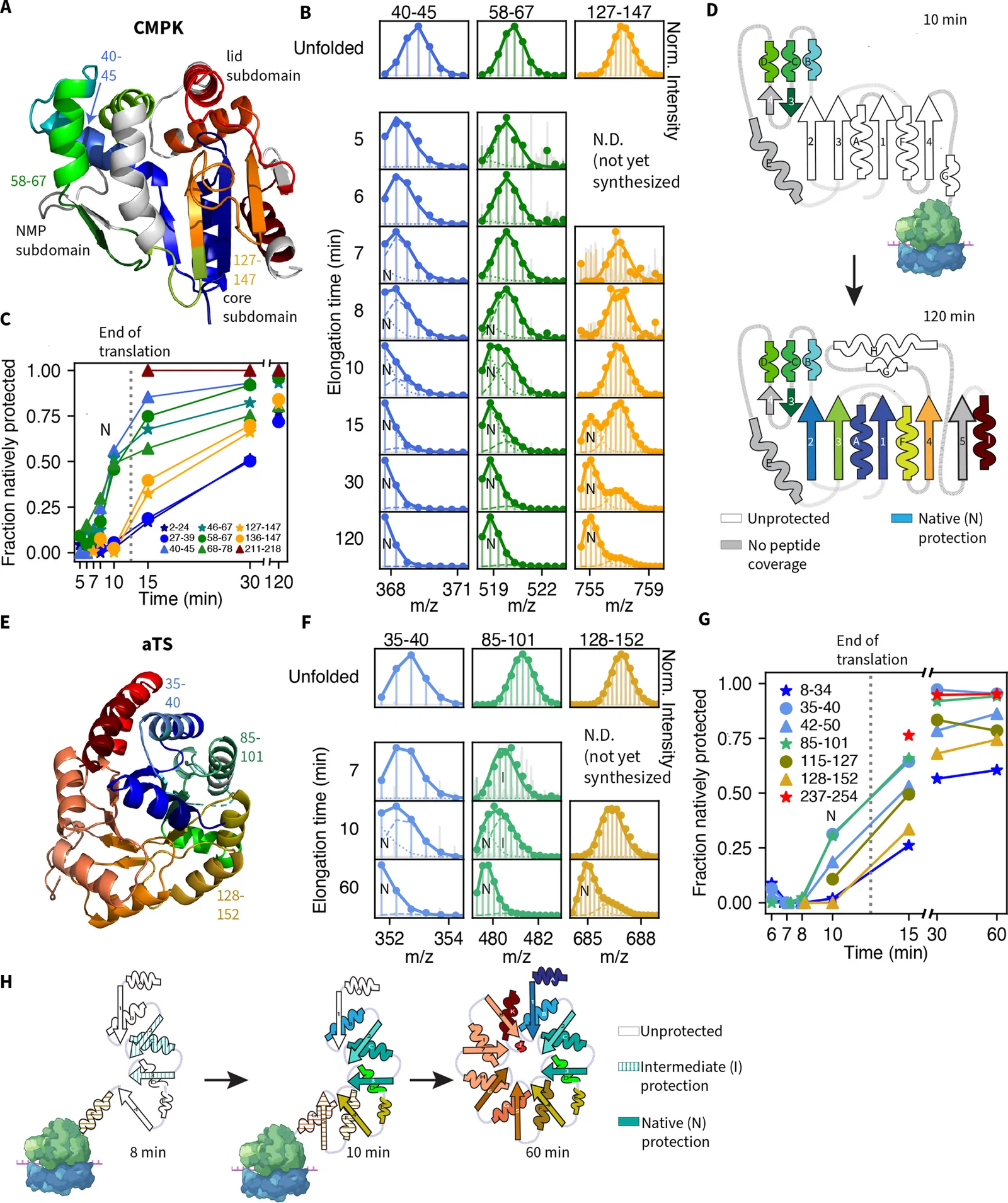
Subdomains begin folding during active translation **(A)** Structure of *E. coli* CMPK (PDB ID *2CMK*) with colored HDX-MS reporter peptides, three of which are labeled. **(B)** Mass spectra (translucent solid lines) vs. elongation time of example deuterated peptides, alongside maximally deuterated spectra (“Unfolded”). Round markers denote isotopic peaks, opaque solid lines indicate fits, and dashed lines show individual mode contributions (N=natively protected mode). **(C)** Fractional population associated with natively protected mode vs. elongation time for indicated peptides. **(D)** Schematic of CMPK co-translational folding. Structures spanned by peptides showing >40% natively protected population at the indicated times are colored solid, provided the native mode is ≤67% deuterated. All other peptides are shown in white, and gray denotes structures lacking peptide coverage. **(E)** Structure of *E. coli* aTS (PDB ID *1WQ5*) with color-coded reporter peptides. **(F)** Mass spectra for indicated aTS peptides as in (B) **(G)** Same as (C) for reporter peptides from aTS. **(H)** Same as (D) for aTS. In this case, solid colors refer to peptides with >10% natively protected population, while dashed colors denote peptides showing statistically significant, albeit partial intermediate protection (see Materials and Methods).

We applied this method to monitor the co-translational folding of various proteins that have been previously predicted to fold co-translationally, beginning with *E. coli* cytidine monophosphate kinase, CMPK, (Fig. 2A) from the P-loop structural family. All-atom simulations predict that CMPK folding begins with the NMP subdomain after roughly 130 AAs have emerged from the ribosome, while core folding is delayed until after translation is complete (29)(fig. S3). We carried out our HDX-MS approach on a synchronized CMPK translation reaction and followed the mass spectra for multiple peptides (Fig. 2A, replicate reaction in fig. S4—see also Supplementary Note 2: Reproducibility analysis) spanning all three subdomains of this protein (spectra in Fig. 2B and fig. S4A).

To elucidate the CMPK folding trajectory, we globally fit the mass spectra associated with each peptide to multiple modes reflecting different levels of deuteration (Fig. 2B-C). For most CMPK peptides, two modes are required, a protected and an unprotected mode (Fig. 2D), as determined by a Bayesian information criterion approach (Materials and Methods, fig. S5). The pattern of deuteration for the protected mode correlates with the expected native secondary structure of CMPK (fig. S4D). Thus, as a proxy for native-like folding, we monitored the fractional population associated with this protected mode as a function of elongation time across all peptides (Fig. 2C and fig. S4). Peptides from the NMP subdomain show a concerted uptick in native protection after 8 mins of elongation, at which point this subdomain has fully emerged from the exit tunnel in the fastest-translating ribosomes (fig. S4B). This result strongly suggests that this subdomain folds co-translationally. In contrast, the core subdomain does not fold until after synthesis is complete (15 mins timepoint), consistent with the core beta-sheet topology involving long-range contacts between the N-and-C termini. We note that for some peptides, a minor population remains unprotected, likely due to a small fraction of prematurely stalled ribosomes that do not complete synthesis (fig. S4E). Peptides from the lid subdomain show only partial protection even in the native state (fig. S4A-C), consistent with the known flexibility in its structural homolog adenylate kinase (47). Thus, CMPK folds through a sequence of intermediates beginning with early co-translational folding of the N-terminal NMP subdomain, followed by the slower rearrangement of the core subdomain, consistent with simulation predictions (Fig. 2D and fig. S3).

We next turned to the TIM barrel family, hypothesizing that we may observe sequential co-translational folding given that the N-terminal halves of some TIM barrels stably and rapidly fold in isolation (48,49). We performed pulse-labeling HDX-MS on a synchronized translation reaction of the well-characterized protein, *E. coli* alpha Tryptophan Synthase (aTS, Fig. 2E, replicate reaction in fig. S5). Indeed, we find that aTS folds co-translationally (Fig. 2F-H), beginning with early acquisition of partial protection of N-terminal structures, particularly the third alpha helix and beta sheet (peptide 85–101, Fig. 2F mode “I”). This is followed by an uptick in native protection from the second alpha helix through the fourth beta strand, and finally, post-translational protection of the C-terminal half barrel and the first beta strand, which must pair with the final strand to close the barrel (Fig. 2G-H). The co-translational folding intermediate seen here closely resembles an intermediate observed upon refolding from denaturant (50) (fig. S6G), indicating that aTS folds via a similar mechanism on and off the ribosome.

## Translation alters the folding pathway of aggregation-prone HaloTag upon release from ribosome

**Figure 3:**
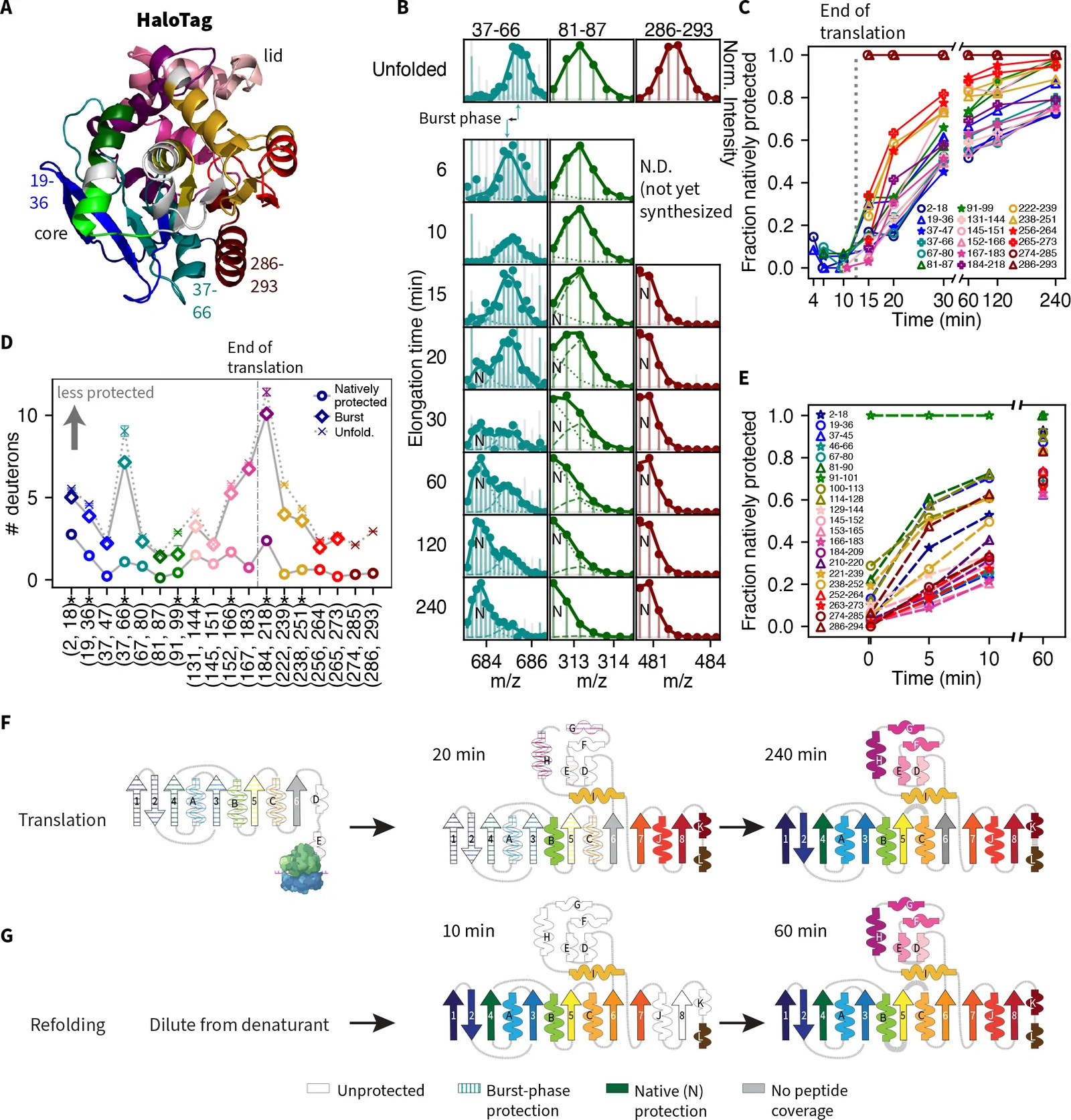
Translation biases HaloTag folding to circumvent aggregation-prone intermediate **(A)** Structure of HaloTag (PDB ID 5Y2Y) with peptides used in HDX-MS analysis colored and mapped onto the structure and subdomains indicated. **(B)** Deuterated peptide mass spectra vs. elongation time for indicated peptides alongside fully deuterated control as in Fig. 2B, highlighting burst-phase protection for 37–66. **(C)** Native population vs. elongation time as in Fig. 2C. **(D)** Number of deuterons associated with natively protected mode (circles), burst-phase mode (diamonds) and unfolded state (Xs) for each peptide. Error bars represent 95% confidence intervals from bootstrapping and asterisks above labels denote peptides exhibiting statistically significant burst-phase protection (Materials and Methods, illustrated in Fig, 2B). Peptides to the right of the dashed line are only detected after 10 mins (post-translation). **(E)** Same as (C) for HaloTag refolding from 7.5M urea, with data re-analyzed from reference (43). **(F)** Schematic of HaloTag co-and-post translational folding with solid colors highlighting structures spanned by at least one peptide showing at least 50% natively protected fraction at the indicated times, striped colors indicating significant burst-phase protection, white indicating no protection, and gray indicating no peptide coverage. **(G)** Same as (F) now depicting Halo Tag refolding from 7.5M urea.

The protein HaloTag (containing an α/β hydrolase fold) achieves a higher yield of natively folded protein during *in vitro* translation compared to refolding from a denatured state (9). To uncover the molecular basis underlying this observation, we performed pulse-labeling HDX-MS on a synchronized HaloTag translation reaction (Fig. 3, replicate reaction in fig. S7)—HaloTag acquires its native structure at the end of translation as shown by its ability to covalently bind the fluorescent ligand TMR (fig. S7C) (9). Pulse-HDX reveals that across the entire protein, emergence of native-like protection is delayed until after translation is complete at which point folding begins with the C-terminus of the core. The N-terminus of the core and lid subdomains fold much later (Fig. 3B, C and F). This sequence of native-structure acquisition contrasts with HaloTag’s folding pathway upon dilution from denaturant (43) where the first detectable intermediate involves both the N- and C-terminal parts of the core (Fig. 3E and G and fig S8). The delayed N-terminal folding relative to the C-terminus in this translation-based experiment is reproducible across replicate translation reactions (fig. S7) and cannot be accounted for by lagging ribosomes (Supplementary Note 3). Instead, this delay indicates that translation induces an N-terminal conformation that is kinetically trapped. The slow resolution of this trap biases post-translational folding to proceed through an alternative pathway, starting from the C-terminus. In support of this model, peptides arising from various N-terminal regions exhibit subtle, but significant nonnative protection early in translation (Fig. 3D) distinct from the burst-phase protection pattern seen upon dilution from denaturant (fig. S8B-D). This is consistent with a translation-induced conformational trap. In summary, translation biases HaloTag’s folding pathway to proceed via a different pathway than upon dilution from denaturant and circumvents the refolding intermediate populated during refolding that has previously been implicated in aggregation (9,43). This likely accounts for the observation that HaloTag achieves a higher folding yield upon translation than upon refolding from denaturant.

## Nascent HaloTag can fold on the ribosome given enough time to reach equilibrium,

**Figure 4:**
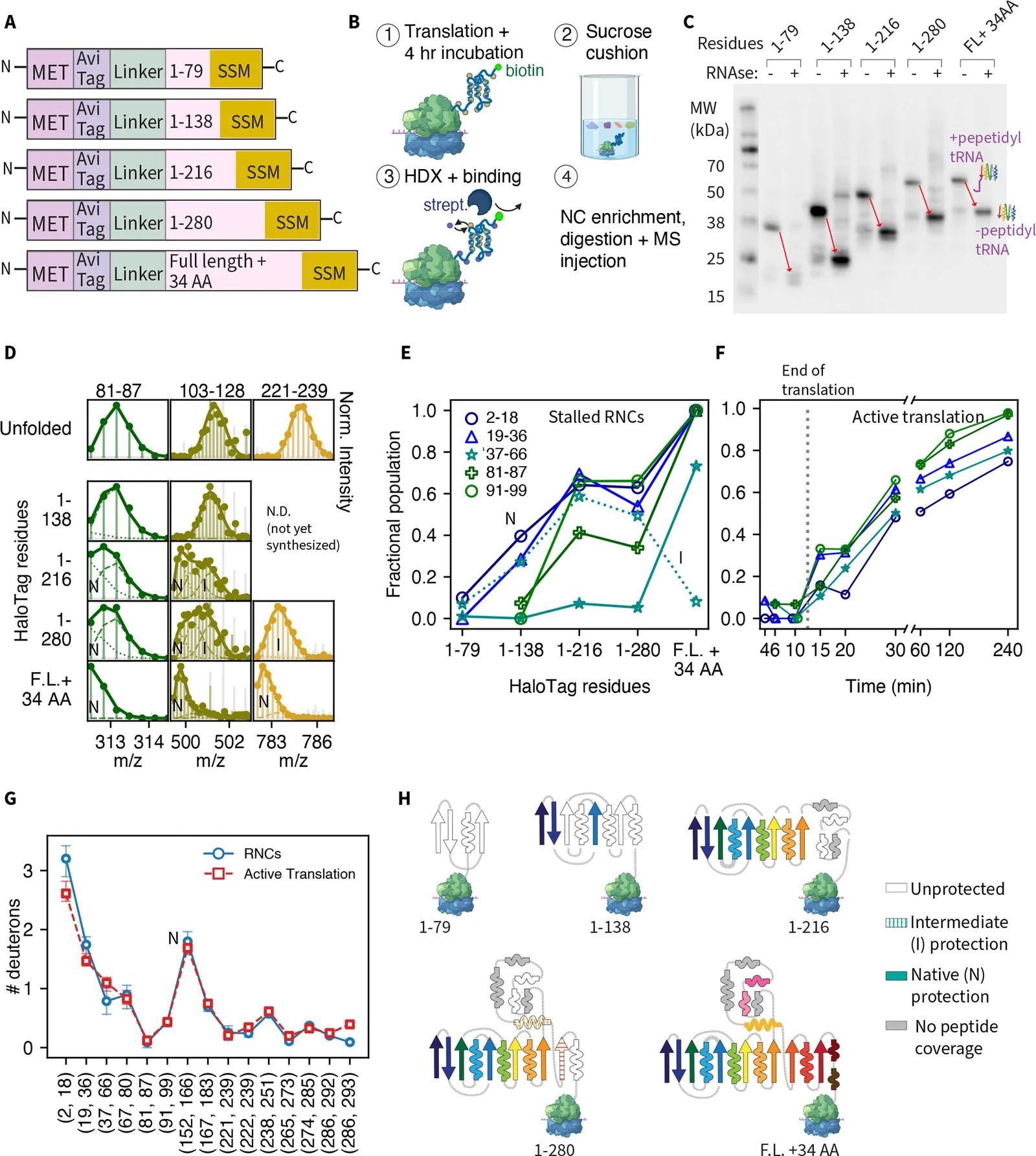
HaloTag nascent chains do not reach equilibrium during active elongation **(A)** Diagrams of constructs used for stalled ribosomal nascent chain (RNC) experiments including strong SecM (SSM) stalling sequence. **(B)** Experimental workflow for RNC preparation and analysis by HDX-MS--see Materials and Methods. **(C)** Streptavidin-HRP western blots showing RNAse-dependent migration shift in RNCs due to peptidyl tRNA attachment (red arrows). **(D)** Deuterated mass spectra for representative peptides from equilibrated RNC constructs alongside maximally deuterated condition (“Unfolded”). **(E)** Fitted fractional populations associated with natively protected mode for indicated peptides from each RNC construct (opaque markers), and intermediate mode for peptide 37–66 (empty markers) obtained from two HDX technical replicates of each sample. **(F)** Same as Fig. 3C for subset of peptides shown in E. **(G)** Number of deuterons associated with natively protected mode (N) for each peptide detected in both HaloTag RNC and active translation experiments. Error bars represent 95% confidence intervals from bootstrapping. **(H)** At each length, secondary structures are shown with solid colors if spanned by at least one peptide showing ≥25% natively protected population, with dashed colors if partially protected, in white if unprotected, and in gray if the region lacks peptide coverage.

HaloTag folds on a significantly slower timescale than that of translation itself (Fig. 3C), suggesting that nascent chains may not have time to reach conformational equilibrium during active elongation. To test this, we realized the equilibrium scenario by generating stalled ribosomal nascent chains (RNCs), which we incubated for four hours before isolation via sucrose cushion ultracentrifugation and then interrogated their folding status via pulse-labeling HDX as was done for actively translating NCs. These RNCs contained HaloTag N-terminal fragments of various lengths coupled to a strong SecM stalling sequence (51) and an N-terminal AviTag (Fig.4A-B). Our second-longest length, HaloTag 280, recapitulates the scenario where HaloTag is fully translated but not yet released from the ribosome, while our longest length (F.L. + 34 AA) contains an additional C-terminal linker to fully span the ribosomal exit tunnel, such that the native HaloTag sequence has fully emerged from the ribosome. We confirmed that our HDX pulse-labeling reports on RNCs that are stably attached to the ribosome (Fig. 4C) (52).

In contrast to actively translating HaloTag, which remains predominantly unprotected on the ribosome (Fig. 3B), equilibrated RNCs show a length-dependent increase in protection across multiple structural regions (Fig. 4D and fig. S9). Various peptides show bi-or-trimodal mass spectra indicative of multiple slowly interconverting populations. We thus quantify length-dependent protection by globally fitting all mass spectra to a combination of modes reflective of these populations (Fig. 4E). At short lengths, N-terminal peptides show minimal, albeit significant protection relative to the unfolded state (Fig. 4D, E, and fig. S9C), resembling the nonnative protection seen early during active translation of HaloTag (fig. S9D and E). However, at increasing RNC lengths, the N-terminus of the core populates increasingly protected states in a length-dependent manner (Fig. 4D, E and H), although this relationship with length is not perfectly monotonic, potentially due to complex stability effects related to distance from the ribosome (53–55). Interestingly, the regions that become protected closely match those protected in the denaturant refolding intermediate (Fig. 4H and 3G), suggesting a similar structure may form in equilibrated RNCs. Moreover, the native-mode protection in RNCs nearly perfectly correlates with that observed at the end of active translation (Fig. 4G, fig. S9D and E), strongly suggesting the protected RNC intermediates are native-like. But crucially, while equilibrated RNCs show length-dependent native protection of various core structures, these same regions do not show comparable protection during active translation until well after synthesis is complete (Fig. 4F). Thus, actively translating HaloTag remains out of equilibrium, as nascent chains do not have time to natively fold while on the ribosome despite their ability to do so.

## Discussion

**Figure 5:**
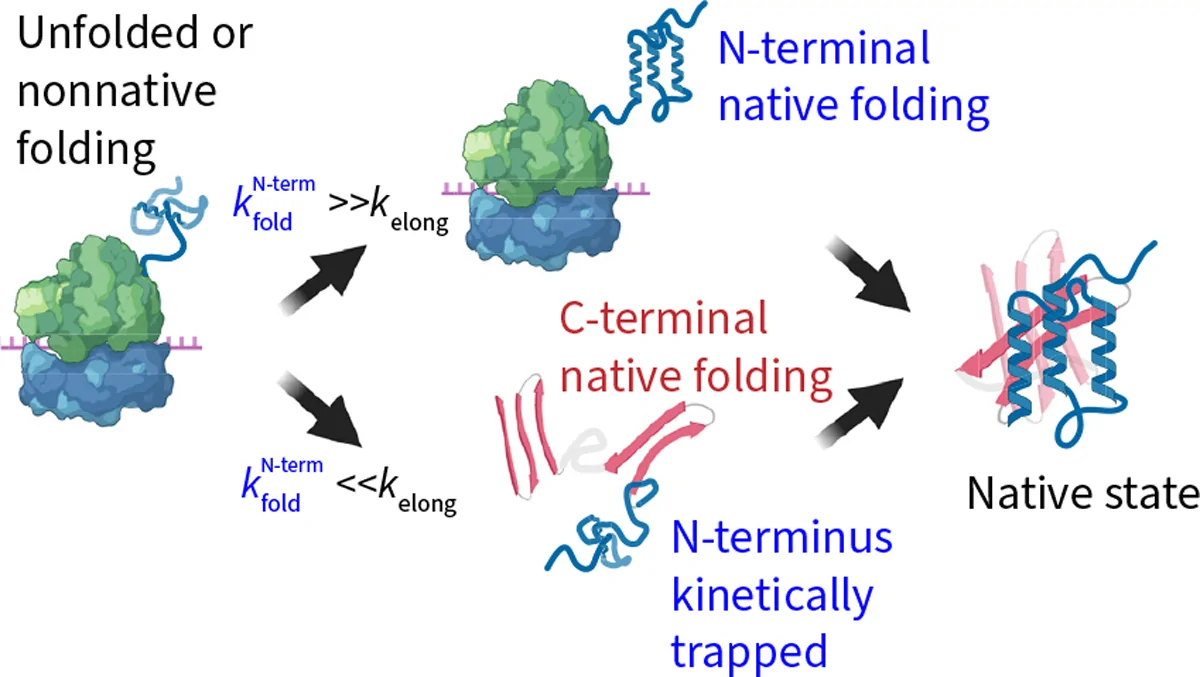
Nascent chain folding depends on kinetic competition between translation and folding rates. Cartoon depicting kinetic competition between elongation and folding during active translation. For details, see discussion.

We developed and implemented an HDX-MS approach to monitor co-translational folding across the nascent chain during active elongation. The ability to follow folding at a high level of structural detail during active elongation provides important insights inaccessible to high-resolution structural studies on stalled nascent chains (10,17–19,53–55) or time-resolved studies of compaction during elongation (35–40). We demonstrate that co-translational folding is shaped by both the thermodynamic drive to fold and the coupling between the relative kinetics of elongation and folding. This coupling results in diverse behavior that is protein dependent. For CMPK and aTS (from the P-loop family and TIM barrel family, respectively), co-translational folding proceeds through a hierarchical series of intermediates involving N-terminal structural elements smaller than a complete domain (Fig. 5, top pathway). Although subdomain-level folding during translation has long been postulated (56), including for TIM barrel proteins (57), our work provides direct experimental evidence of this process during active elongation. Atomistic simulations of CMPK using the DBFOLD algorithm (58) recapitulate our observed folding hierarchy and generate experimentally consistent structures of nascent chain intermediates (fig. S3), further highlighting the power of this and related approaches to predict and interpret co-translational folding mechanisms (40). For aTS, the co-translational intermediate we observe closely resembles an obligatory N-terminal denaturant refolding intermediate previously observed by HDX-MS (fig. S6G), suggesting the two folding mechanisms are similar (50). But, for the large number of TIM barrels that do not refold efficiently from denaturant (11,13,14), sequential co-translational folding as observed here may be critical for ensuring robust folding of this topologically complex structure. More broadly, this strategy may enable proteins to circumvent deep post-translational folding traps (5–8,29,30,59).

In contrast, HaloTag does not begin exhibiting native-like folding until after its release from the ribosome, even though the stalled RNC experiments demonstrate that nascent HaloTag is capable of folding on the ribosome. The timescale required for it to do so is much longer than the timescale of translation elongation. Instead, actively translating HaloTag shows minimal protection from HDX on the ribosome, but counterintuitively begins folding from the C-terminus upon its subsequent release from the ribosome (Fig. 5 bottom pathway). This indicates that the N-terminus becomes kinetically trapped during elongation and thereby doesn’t sample the aggregation-prone intermediate populated during refolding from denaturant which involves both termini. This intermediate resembles the conformation formed by stalled HaloTag RNCs that have been given enough time to reach equilibrium (Fig. 3G and 4H). Thus, the non-equilibrium nature of co-translational folding is likely crucial in allowing the protein to avoid this problematic intermediate through a chaperone-independent mechanism and thus attain a high folding yield (9).

The structural nature of the kinetic trap during active HaloTag translation remains to be fully elucidated. Nevertheless, our observation of significant, albeit partial, nonnative protection at early translation times are consistent with either 1) a single conformation with a small subset of native hydrogen bonds formed, or 2) a dynamic ensemble of nonnative conformations that readily exchange with each other, akin to that observed on the ribosome for the protein HemK (37–40). In either case, the barrier to escape this state must be large enough to trap the N-terminus until well after translation is complete, thus biasing the folding pathway. More broadly, our results underscore the importance of assays like the one developed here to monitor folding during active elongation, as non-equilibrium folding during translation can differ starkly from that seen in equilibrated RNCs, as noted in single molecule mechanical studies on calerythrin (36).

It is important to note that the rate of translation elongation in PURE-based *in vitro* translation assays such as ours (~0.5–1 AA/sec, consistent with previously measured rates PURE system (9)) is significantly slower than the *in vivo* elongation rate in bacterial systems such as *E. coli*, from which PURE components are derived (~10–20 AA/sec). However, for the case of HaloTag, we expect that a faster rate of translation would only *further* heighten the discrepancy between equilibrium and real-time co-translational folding, provided the kinetic trap we observe forms on rapid timescales. In contrast, aTS very rapidly refolds from denaturant (<1 sec timscales) into an obligatory N-terminal refolding intermediate closely resembling the co-translational intermediate observed here (50) suggesting it may also form under a faster elongation rate. Closing the gap between the *in vitro* and *in vivo* elongation rates will enable detailed investigations on the effects of the relative rates of translation. This includes the role of conserved rare codons such as those in the CMPK sequence shortly after the NMP-binding subdomain (27,29) which folds co-translationally in our assay.

In summary, our work reveals a diversity of strategies that are deployed by the ribosome to promote robust protein folding, from promoting sequential folding of subdomain elements to *delaying* folding to avoid problematic intermediates. Over the course of evolution, a protein’s fitness may depend on its ability to exploit these folding mechanisms, and future work is needed to extend equilibrium biophysical fitness models (60–62) to account for non-equilibrium co-translational folding. Viral proteins, in particular, rely heavily on such mechanisms to fold robustly during infection (63). In the cell, co-translational folding is further influenced by chaperones and quaternary assembly partners that interact with nascent chains. In the future, the methodological platform presented here can be extended to account for these more complex mechanisms and reveal possible links between co-translational (mis)folding and disease. Finally, these findings can be exploited to enhance the efficiency of *de novo* protein design and transgenic protein expression by optimizing for kinetic folding yield.

## Materials and Methods

### Cloning and PCR

Genes encoding proteins of interest used for synchronized translation reactions were cloned via Gibson assembly into a plasmid encoding an ampicillin resistance marker. The gene was inserted downstream of an AviTag as well as a linker region containing a threonine, a stretch of serines and glycines (41 residues for HaloTag, 54 for aTS and CMPK), and three valines. The *cmk* and *TrpA* genes encoding the proteins CMPK and aTS were amplified directly from the *E. coli* K-12 chromosome via PCR, using primers directed against the 5’ and 3’ ends of the gene prior to Gibson assembly. The identities of the inserts on the respective plasmids were verified by Sanger sequencing.

As a template for transcription and synchronized translation reactions, the genes of interest were reamplified from their harboring plasmids using a forward primer directed upstream of the T7 promoter and a reverse primer downstream of the stop codon. The resulting PCR product was DpnI-digested to remove the template plasmid, followed by PCR purification on a silica gel column and elution using molecular biology grade water (Corning 46–000). Purity and yield were assessed via NanoDrop. Any PCR products showing an $A_{260}/A_{230}$ absorbance ratio less than 1.8 were further purified using a 0.5 mL Zeba spin desalt column (Thermo Scientific 89883) to remove residual salts.

For stalled ribosome-nascent chain (RNC) experiments, the HaloTag gene, alongside an N-terminal AviTag and linker (as described above), was cloned into a plasmid containing a 17 AA C-terminal linker (GSGSGSGSGSEFLPYRQ) followed by the 17 AA strong SecM stalling sequence (FSTPVWIWWWPRIRGPP) using around-the-horn (ATH) PCR. In total, this C-terminal region (linker + SecM) contains 34 amino acids. This plasmid was then used as a template for subsequent ATH PCRs to generate plasmids consisting of various HaloTag C-terminal truncations (HaloTag 1–79, 1–138, 1–216, and 1–280) all followed by the strong SecM stalling sequence (but no linker). Prior to *in vitro* translation, the regions spanning the T7 promoter through the strong SecM stalling sequence were amplified from each plasmid via PCR, and the resulting PCR fragments were purified as described in the previous paragraph. In addition to the truncations, we generated a PCR fragment to encode an RNC harboring full-length HaloTag sequence and the 34 AA C-terminal region (linker + strong SecM) to fully span the ribosomal exit tunnel, thus ensuring that HaloTag is fully emerged from the ribosome.

### Synchronized Translation Reactions

Synchronized translation reactions are performed in two steps: (1) mRNA transcription and initiation, followed by (2) single-turnover elongation. The transcription and initiation steps are expected to be slow; thus, it is advantageous to complete them first to maximize subsequent synchrony.

For the transcription and initiation step, reactions are prepared with methionine as the sole provided amino acid to allow mRNA to be transcribed and initiation complexes to form without elongating. This is achieved by preparing two to four PUREexpress reaction equivalents, depending on the number of timepoints to be taken for HDX-MS. Each HDX-MS timepoint requires 6 μL of material, with an additional 2 μL for western blot samples. Each reaction equivalent consists of:

- 5 μL of Solution A –aa, tRNA (New England Biolabs B6841AVIAL from E6840S)
- 7.5 μL Solution B –RF123 (NEB P6854AVIAL)
- 1 μL Murine RNAse inhibitor (NEB M0314L)
- 1.25 μL of 20 μM BirA
- 2.5 μL of *E. coli* tRNA mix (NEB N6842AVIAL)
- 0.5 μL of 30 mM methionine
- 0.5 μL of 1 mM biotin (20 μM final)
- 260 ng of purified PCR product encoding the gene of interest downstream of an AviTag + glycine-serine linker (see previous section) or an equivalent volume of molecular biology grade water (for negative control reactions)
- Molecular biology grade water to bring the total volume to 30 μL.

The optimal biotin concentration of 20 μM was determined by a streptavidin bead binding experiment shown in fig. S1H. A formyl donor for the formation of fMet-tRNAs is present in Solution A, and the addition of tRNAs at this step provides a source of methionine tRNA. In certain control experiments, we added 1 μL of FluoroTect GreenLys (Promega L5001)—a lysine tRNA charged with a BODIPY-FL labeled lysine—to facilitate in-gel visualization of the translated product without requiring western blotting; however, FluoroTect is excluded from reactions destined for HDX-MS analysis. This reaction is incubated for 30 min at 37°C to allow transcription to occur and initiation complexes to form. Where indicated, a HaloTag TMR Ligand (Promega G825A) was added to a 10 μM final concentration at this stage.

During the initiation period, a working elongation mix is prepared by mixing 2.5 μL of complete amino acid mix (NEB N6843AVIAL); 0.5 μL each of release factor 1 (NEB P6851AVIAL), release factor 2 (P6852AVIAL), and release factor 3 (P6853AVIAL); at least 2.5 μL of pre-charged tRNA for a final concentration of >20 μM pre-charged tRNA (>1 μM each); and 0.95 μL of 2.37 mM aurintricarboxylic acid (ATCA, MilliporeSigma 18–940-0100MG) for a final working concentration of 75 μM per reaction equivalent. ATCA is used to prevent the reinitiation of ribosomes and ensure single-turnover elongation. We confirmed that translation is inhibited when ATCA is added during the initiation step, but not when added at the elongation step (fig. S1A). The ATCA solution is prepared fresh from powder on the day of use, stored on ice, and protected from light. The working elongation mix is prepared under RNAse-free conditions and pre-warmed at 37°C for 10 min prior to the onset of elongation.

After 30 min of initiation, 7.45 μL of elongation mix is added per reaction equivalent of initiation mix to begin elongation. The N-terminal AviTag in the nascent chains becomes rapidly biotinylated after its emergence, with significant biotinylation already occurring after 3 minutes as shown in fig. S1G. At the desired elongation timepoints, a 6 μL aliquot of the synchronized elongation reaction is drawn to perform pulse HDX and nascent chain enrichment (see section Pulse HDX-MS, Maximally Deuterated Sample Prep, and Nascent Chain Enrichment). In addition, at these same timepoints, 1.8 μL of the reaction is drawn and mixed with 3.6 μL of 2 mM puromycin (1.33 mM final) to generate samples for western blot analysis. These samples are kept at room temperature for at least 30 s and then placed on ice. They are subsequently treated with RNAse A (Thermo Scientific EN0531, 0.5 mg/mL final) for 5 min at 37°C, followed by the addition of 5.4 μL of 2 × Laemmli sample buffer prior to SDS-PAGE and/or western blot analysis. After collecting the final timepoint, 6 μL of the sample was bound to streptavidin beads pre-equilibrated in H_2_O for the preparation of an undeuterated control, and two 6 μL aliquots were used to prepare maximally deuterated samples.

Although the main text figures show one biological replicate for each active translation reaction, we performed at least two total replicates for each protein; additional replicates are shown in the supplementary figures. We verified that our main results—particularly the order of folding of distinct structural elements before and after the end of translation—reproduce across biological replicates. The precise times at which events occur vary slightly between replicates due to ribosomal batch variations that lead to minor variability in translation rates.

### Preparation of Pre-charged tRNA

Total *E. coli* tRNA (6.25 mg, Roche MRE600) was weighed and resuspended in 280 μL of molecular-biology grade water. This was mixed with 50 μL of a complete amino acid mixture containing roughly 2.2 mM of each amino acid at near-neutral pH, 20 μL of 100 mM ATP, 50 μL of 10 × charging buffer (final 1 × composition: 50 mM K-HEPES pH 7.5, 50 mM KCl, and 5 mM DTT), and 100 μL of DEAE-purified S-100 extract (64). This reaction was incubated for 1 h at 37°C, followed by the addition of 80 μL of NaOAc or KOAc pH 5.3 (final concentration of 300 mM) to acidify the reaction and maintain ester bond stability.

Protein components were then removed by phenol-chloroform extraction using cold, saturated phenol (pH 5.3). The tRNA was precipitated overnight at −80°C with 100% ethanol, followed by centrifugation for 2 h at 14,000 × *g* at 4°C. The supernatant was removed and the pellet was air-dried for 5–10 min, followed by resuspension in water (pH 5.3) and desalting through an illustra MicroSpin G-25 spin column (Cytiva 27–5325-01) pre-equilibrated in 3 mM KOAc. Following this stage, absorbance at 260 nm was measured using a NanoDrop to determine tRNA concentration. Pre-charged tRNA was aliquoted, flash-frozen in liquid nitrogen, and stored at −80°C.

### Preparation of Stalled Ribosomal Nascent Chain Samples

A translation master mix was prepared under RNAse-free conditions, consisting of:

- 15 μL Solution A –aa, tRNA (New England Biolabs B6841AVIAL from E6840S)
- 22.5 μL Solution B –RF123 (NEB P6854AVIAL)
- 3 μL Murine RNAse inhibitor (NEB M0314L)
- 3.75 μL of 20 μM BirA (0.8 μM final)
- 7.5 μL of *E. coli* tRNA mix (NEB N6842AVIAL)
- 7.5 μL of complete amino acid mix (NEB N6843AVIAL)
- 1.5 μL of 1 mM biotin (17 μM final)

This master mix was then split into 6 equal aliquots corresponding to the five HaloTag RNC constructs to be generated, with duplicate reactions for the L34 construct (which contains the 34 AA combined C-terminal linker + strong SecM sequence to fully span the exit tunnel, see Cloning and PCR) to ensure sufficient volume for undeuterated and maximally deuterated controls. To each aliquot, 130 ng of PCR product encoding the respective gene construct (including the N-terminal AviTag + linker and C-terminal strong SecM stalling sequence) was added, along with molecular biology grade water to a total volume of 15 μL. The reactions were incubated for 4 h at 37°C to allow for complete translation and equilibration of nascent chains.

Subsequently, 14 μL of each reaction was layered atop a 94 μL sucrose cushion consisting of 1 M sucrose in HKMT buffer (25 mM HEPES, 15 mM Mg(OAc)_2_, 150 mM KCl, 0.1 mM TCEP, pH 7.5) in polycarbonate centrifuge tubes (Beckman Coulter 343775). These samples were ultracentrifuged in a Beckman Coulter Optima MAX-XP ultracentrifuge for 80 min at 200,000 × *g* and 4°C using a pre-chilled Beckman Coulter TLA-100 fixed-angle rotor. Following the removal of the supernatant, the ribosomal pellets were resuspended in 20 μL of HKMT buffer, and the two resuspended pellets corresponding to the L34 construct were pooled. All resuspended pellets were re-equilibrated to 37°C for 15 min, followed by pulse-labeling HDX and streptavidin bead binding as described in the subsequent section. Two technical replicates of the pulse-labeling reaction were performed for each sample. The L34 construct was also used to generate undeuterated and maximally deuterated control samples.

Following pulse-HDX, the integrity of the nascent chains (NCs) was assessed by mixing 2 μL of resuspended pellet with 10 μL of low-pH sample buffer for a final composition of 7.5% glycerol, 62.5 mM Bis-Tris pH 5.7, 0.01% bromophenol blue (wt/vol), 0.2% DTT (wt/vol), and 2% SDS (wt/vol) (65). These samples were analyzed by SDS-PAGE and western blotting (see section *Anti-biotin Western Blotting*) to ensure homogeneity of each NC construct and to verify a migration pattern approximately 20–25 kDa larger than expected due to peptidyl-tRNA attachment. To generate “released” samples, a separate aliquot of each construct was treated with RNAse A (final concentration of 0.2 mg/mL, Thermo Scientific EN0531) and incubated for 5 min at **37°C** prior to the addition of low-pH loading buffer and SDS-PAGE analysis.

### TEV Elution Experiment

To confirm that streptavidin bead binding does not disrupt nascent chain (NC) integrity, we designed an experiment in which the AviTag is separated from the NC by a linker ending with a TEV cleavage loop; following bead binding, NCs were eluted via the addition of TEV protease. The TEV cleavage loop (ENLYFQ) was inserted at the end of the linker separating the AviTag from the nascent protein of interest (in this case, aTS), and a PCR fragment for *in vitro* transcription and translation was produced as described in subsection Cloning and PCR. This PCR fragment was designed to contain a 35 amino acid C-terminal linker downstream of aTS and lacked a stop codon, thereby promoting ribosomal stalling at the end of this linker.

A translation master mix was prepared consisting of:

- 5 μL Solution A –AA, tRNA
- 2.5 μL of 10 × AA mix
- 2.5 μL tRNA
- 7.5 μL of Solution B-RF123
- 1 μL Murine RNAse inhibitor
- 1 μL of FluoroTect GreenLys
- 1.25 μL of 20 μM BirA
- 270 ng PCR fragment

This master mix was split into three samples (A, B, and C). Samples A and B were supplemented with biotin to a 20 μM final concentration, while sample C was supplemented with an equivalent volume of water. All three samples were incubated at 37°C for 1 h to allow translation to occur. After 1 h, sample B was treated with puromycin to a final concentration of 68 μM to release nascent chains. At this stage, a 2 μL “input” aliquot was drawn from all three samples, mixed with 8 μL of HKMT buffer and low-pH loading dye (to a 1 × working concentration), and kept on ice.

A 6 μL aliquot from each sample was then incubated for 10 s at 37°C with a 54 μL streptavidin bead aliquot pre-equilibrated in D_2_O (see previous sections). After 10 s, the samples were placed on a magnetic stand and the supernatant was removed. The beads were resuspended in 30 μL of a protease mix containing 3 μM TEV protease purified in-house and 1 μL of murine RNAse inhibitor in HKMT buffer, and then incubated for 30 min at room temperature to allow TEV cleavage and nascent chain elution. Samples were placed on a magnetic stand, and 27 μL of the supernatant was recovered and mixed with 9 μL of 4 × low-pH loading dye. All samples were loaded onto a NuPAGE gel, which was run in a cold room (4°C), and the gel was imaged using a Typhoon system as described in SDS-PAGE and Anti-biotin Western Blotting.

### SDS-PAGE and Anti-biotin Western Blotting

Samples in 1 × Laemmli sample buffer (for active translation reactions) or low-pH sample buffer (for stalled RNCs or other samples requiring preservation of peptidyl-tRNA integrity) were loaded onto a NuPAGE 4–12%, 1.5 mm, 15-well gel. We included 2 μL of WesternSure Pre-stained Chemiluminescent Protein Ladder (LI-COR, 926–98000) for western blot samples, or 0.5 μL of PageRuler Plus Prestained Protein Ladder (Thermo Scientific 26619) for samples destined for fluorescence imaging. Gels were run at 125–130 V at room temperature, or at 4°C (for samples requiring preservation of peptidyl-tRNA integrity), for at least 1 h in MES running buffer. If gel samples were fluorescently labeled with FluoroTect, the gel was imaged using a Typhoon FLA9500 (GE Healthcare) with a 488 nm laser and a 510LP filter. If labeled with TMR, a 532 nm excitation laser and a 580 nm BP emission filter were used.

Prior to western blot transfer, gels were rinsed in deionized water and equilibrated for 10 min in 1 × Towbin transfer buffer containing 20% methanol, alongside blotting paper and a 0.2 μm polyvinylidene difluoride (PVDF) membrane (Bio-Rad, 1620174) that had been pre-activated in 100% methanol. Transfer was performed at 25 V for 42 min using a Trans-Blot Turbo apparatus. The membrane was then rinsed in deionized water and incubated with 10 mL EveryBlot Blocking Buffer (Bio-Rad, 12010020) on an orbital shaker for 5 min at room temperature, or overnight at 4°C. The blocking buffer was decanted, and the membrane was incubated in a streptavidin solution consisting of 0.5 μL of 2 mg/mL Streptavidin-horseradish peroxidase (HRP) conjugate (Invitrogen S911A) in 10 mL EveryBlot Blocking Buffer for 1 h at room temperature with orbital shaking. The membrane was then washed five times for 5 min each with Tris-buffered saline solution containing Tween-20 (TBST). Prior to chemiluminescent imaging, the membrane was rinsed in deionized water and blotted dry. The blot was imaged via the addition of either 5 mL of each solution of Clarity Western ECL substrate (Bio-Rad 1705061), 6 mL of each solution of SuperSignal West Pico PLUS Chemiluminescent Substrate (Bio-Rad, 34580), or 2 mL of each solution of SuperSignal West Femto substrate (Thermo 34094). The membrane was imaged on a ChemiDoc system. Where indicated, an Alexa Fluor 488 Streptavidin conjugate (Invitrogen A32361) was used in lieu of Streptavidin-HRP, and imaging was carried out using an FLA9500 (GE Healthcare) with a 488 nm laser.

### Pulse HDX-MS, Maximally Deuterated Sample Prep, and Nascent Chain Enrichment

Pierce streptavidin magnetic beads (Thermo Scientific 88816) were equilibrated to room temperature and resuspended, and 60 μL of bead slurry was drawn per translation timepoint. The beads were placed on a magnetic stand to remove the storage solution and subsequently washed with 1 mL of nascent chain labeling buffer (10 mM MES, 30 mM KOAc, 12 mM Mg(OAc)_2_, pH 6.0) in H_2_O. Beads used for deuterated samples were additionally washed in 0.5 mL of nascent chain labeling buffer in prepared in 99.8% D_2_O (TCI W002 or Thermo 426931000). The beads were then resuspended in 54 μL of nascent chain labeling buffer in D_2_O (for deuterated samples) or H_2_O (for undeuterated controls) per timepoint, and 54.3 μL aliquots corresponding to the respective timepoints of interest were transferred into 0.5 mL Protein LoBind Tubes (Eppendorf 022431064).

These aliquots were pre-warmed for 10 min at 37°C prior to use in the pulse HDX-MS reaction. A pD value of 6.0 (as read directly by a pH meter) was chosen for pulse HDX to ensure that a 10 s labeling pulse would label solvent-exposed amides and protect regions that are folded with some level of stability. predominantly label solvent-exposed amides. We calculated that under EX2 conditions, this pH and pulse duration would prevent labeling of any structure formed with a stability greater than approximately 2 kcal/mol at 37°C (66).

At indicated elongation timepoints, nascent chains were pulse-labeled and bound to streptavidin beads. This was achieved by drawing 6 μL of elongation reaction (see section Synchronized Translation Reactions) and thoroughly mixing it with the respective bead aliquot in deuterated or undeuterated buffer (prepared as described in the previous paragraph). The sample was incubated for 10 s at 37°C to allow hydrogen-deuterium exchange (for deuterated samples) and nascent chain binding to the streptavidin beads, followed by the addition of 60 μL of 2 × ice-cold quench buffer (3.5 M GdmCl, 1.5 M glycine, and 0.5 M TCEP, pH 2.4) and rapid mixing. Samples were then flash-frozen in liquid nitrogen and stored at −80°C until LC-MS injection.

Maximally deuterated control samples were prepared by mixing 6 μL of nascent chains with a 54.3 μL streptavidin bead aliquot pre-equilibrated in D_2_O, followed by a 15 min incubation at room temperature on a rotary mixer to allow complete binding of nascent chains to the beads. The samples were placed on a magnetic stand and the supernatant was removed. The beads were then resuspended in 60 μL of 8 M urea + NC labeling buffer in 90% D_2_O. This buffer was prepared by dissolving urea to 9 M in NC labeling buffer in H_2_O, followed by three rounds of lyophilization and resuspension in 90% D_2_O; the final urea concentration was adjusted to 8 M (as measured by refractometry) using NC labeling buffer in 90% D_2_O. Beads were incubated overnight at 37°C in this deuterated urea buffer, followed by the addition of 60 μL of ice-cold 2 × quench buffer. Samples were then flash-frozen in liquid nitrogen and stored at −80°C until LC-MS injection. For the first CMPK biological replicate, maximally deuterated samples were incubated at 37°C for 3 h rather than overnight; we confirmed this duration is sufficient to produce maximal deuteration. Maximally deuterated controls were prepared in duplicate for each sample.

Prior to LC-MS injection, samples of all types were thawed by rapid flicking and passed through a Corning Costar Spin-X 0.22 μm filter (Costar 8161) by centrifuging for 30 s at 12,000 × *g* to separate the quenched labeling solution from the beads, which are retained in the filter basket. The beads were then resuspended in 1 × quench buffer (2 × quench buffer diluted in NC labeling buffer in H_2_O) containing 0.2 mg/mL of porcine pepsin (Sigma P6887) to initiate digestion. A total digestion time of 4 min was found to yield optimal peptide coverage for HaloTag samples, while 5.5 min was found to be optimal for CMPK and aTS samples.Immediately after resuspension in the pepsin-containing 1 × quench, the beads were vortexed for 5 s and placed on ice. Two minutes prior to the end of digestion, the bead-containing centrifugal filter was centrifuged again for 30 s at 12,000 × *g*, and the flow-through containing the peptides was collected in a clean 1.5 mL LowBind tube and returned to ice until the end of the digestion period. At this point, the liquid sample was injected into the LC-MS system.

### LC-MS Runs

Peptide-containing samples were injected into a cooled valve system (Trajan LEAP) in line with a Thermo Ultimate 3000 LC. Peptides were desalted on a C8 pre-column (Waters 186003978) for 4 min, and subsequently bound to a Waters Acquity C18 analytical column (Waters 86002344); both columns were maintained at 2°C. The bound peptides were gradient-eluted (5–40% acetonitrile w/v and 0.1% w/v formic acid) across the analytical column at a flow rate of 40 μL/min over 14 min, followed by a gradient from 40% to 90% buffer B over 30 s. The analytical and trap columns were then sawtooth-washed and equilibrated at 5% buffer B. Eluted peptides were analyzed directly using a high-resolution Orbitrap mass spectrometer (Q Exactive, Thermo Fisher) operating in positive mode [resolution: 140,000; automatic gain control (AGC) target: 3 × 10^6^; maximum injection time: 200 ms; scan range: 300 to 1500 mass/charge ratio (m/z)]. For each construct, we performed a tandem mass spectrometry (MS/MS) experiment on an undeuterated sample [full MS resolution: 70,000; AGC target: 3 × 10^6^; maximum injection time: 50 ms; scan range: 300 to 1500 m/z; dd-MS2 settings: resolution: 17,500; AGC target: 2 × 10^5^; maximum injection time: 100 ms; loop count: 10; isolation window: 2.0 m/z; normalized collision energy: 28; charge states 1 and ≥ 8 excluded; dynamic exclusion: 15 s]. Between injections, the sample loop and trap column were manually washed at 200 μL/min with 50% acetonitrile + 0.1% formic acid for 4 min, followed by one wash of the protease column with 100 μL of 1.6 M GdmCl and 0.1% formic acid, and three sawtooth washes of the trap and analytical columns (5–90% acetonitrile gradient each).

### Mass Spectrometry Data Analysis

Our basic data analysis pipeline involves the following steps:

1. **Peptide identification:** Observed MS1 and MS2 spectra from undeuterated control samples are assigned to peptides derived either from the protein of interest or from other protein components in the translation and enrichment pipeline.
2. **Manual inspection and curation** of peptide mass spectra from deuterated samples.
3. **Fitting of mass spectra** to multimodal distributions and identification of the optimal number of modes.
4. **Bootstrapping** error analysis.

### Peptide Identification

RAW files from undeuterated control injections, containing precursor and fragment mass spectra (MS1 and MS2, respectively) across all retention times, are loaded into Byonic (Protein Metrics) software. For peptide identification, we input a FASTA file containing sequences for all *in vitro* translation components present in PUREexpress reactions, as well as protein components from the nascent chain enrichment steps (namely, streptavidin and pepsin) and the translated protein of interest downstream of the AviTag and its respective linker.

The following peptide search parameters are used:

- Precursor mass tolerance: 6 ppm
- Fragmentation type: QTOF/HCD
- Fragment mass tolerance: 20 ppm
- Digestion specificity: Non-specific
- Maximum precursor mass: 10,000
- Number of precursors per MS2: 1
- Smoothing width (m/z): 0.01

We retain all peptide and charge state identifications associated with a Pep2D score <0.1, and export these spectra and associated retention times for subsequent analysis.

### Manual Inspection of Peptide Spectra

RAW files corresponding to all deuterated samples are loaded into HDExaminer 3 (Sierra Analytics) software, alongside the peptide lists and associated retention times from the previous step. Using HDExaminer, we manually inspect deuterated mass spectra assignments for all peptides to ensure that intensity peaks are centered about their expected m/z isotopic windows, and that the retention times at which the peptide spectra are observed differ from those in undeuterated spectra by no more than ~ 0.2 min. If any retention time shifts are observed for a given peptide, we verify that such a shift is systematically observed across all peptides within that sample.

We additionally adjust the integrated retention time windows to ensure that these integration ranges are comparable across samples for each specific peptide. As an additional quality control step, we examine the corresponding m/z and retention time ranges in a negative control sample for each peptide to rule out interfering or carry-over signals. For each sample, we discard any peptide spectra for which the negative control signal in relevant m/z windows (namely, all isotopic windows ranging from the monoisotopic peak to the largest meaningful window containing signal in the maximally deuterated control) is more than one-fifth of the signal within the sample. This threshold indicates that the background is expected to contribute meaningfully to the observed signal. This criterion excludes interference from both background peptides whose spectra overlap with the target peptide and carryover; the latter is observed only in a small fraction of peptides. Following manual curation, all resulting high-confidence peptide mass spectra are exported and fit as described below. These high-quality peptides are shown as colored vertical lines in the coverage maps for each protein construct in Fig. S2.

### Peptide Spectra Fitting

During co-translational protein folding, we expect different structural regions to cooperatively fold and attain protection from deuterium exchange in a time-dependent manner. Because the pulse timescale (10 secs) is significantly shorter than the timescale of translation, we obtain a snapshot of the conformations populated at the time of the pulse. These conformations have different protection patterns, manifesting as peptide mass spectra with distinct peaks representing their respective level of protection.

For each peptide, we globally fit all mass spectra from distinct co-translational folding timepoints to a combination of basis spectra indexed by m corresponding to these distinct conformational states. We assume that the centroids themselves are constant across timepoints but allow the relative contribution of each basis (its weight) to vary. That is, for a peptide with charge state z, we assume the mass spectral intensity at mass-to-charge value m/z and translation time t is given by:

$$I_{m/z,t}=A\sum_{m=1}^{N_{c}}f_{t}^{m}\sum_{i=0}^{N}Binom\left(i;N_{eff}^{m},p_{m}\right)*I_{0}\left(\frac{\left(m-i\right)}{z}\right)$$

Where A describes the signal amplitude related to factors such as digestion and ionization efficiency, $N_{c}$ is the number of curves being fit, $f_{t}^{m}$ is the fraction of signal associated with mode m at time t (at each timepoint, these must sum to 1 over all m), N is the total number of exchangeable amides in the peptide, $Binom\left(i;N_{eff}^{m},p_{m}\right)$ refers to a normalized binomial distribution evaluated at integer i with parameters $N_{eff}^{m}$ and $p_{m}$ (the product of which is the average number of deuterons taken up for mode m), and $I_{0}(m/z)$ is the mass spectral intensity for the natural abundance isotopic distribution for the undeuterated peptide at m/z. In this formulation, we neglect the instrument-induced mass spectrometric peak width as well as any interference from other overlapping peptides.

To analyze data for a given peptide, we globally fit the above equation to the mass spectrometric signal at all isotopic peaks across all timepoints, optimized for parameters $A,\;\left\{f_{t}^{m}\right\},\;N_{eff}^{m}$, and $p_{m}$. The $\left\{f_{t}^{m}\right\}$ parameters reveal the population fractions associated with each mode at each translation time, while the product $N_{eff}^{m}p_{m}$ gives the centroid for mode m. Where technical replicates exist for a given condition (in the case of stalled RNCs), we required that both replicates be fit to the same parameters corresponding to that respective condition. Fitting is implemented in custom Python scripts using the SciPy package. Our approach and fitting algorithm, including our calculation of the natural isotopic distribution, are largely based on those in reference (67), but adapted to allow for global fitting across timepoints.

To decide on an optimal number of curves $N_{c}$, we balance attaining a high-quality fit with the risk of overfitting. This is achieved in two steps. First, we compute the Bayesian information criterion (BIC) associated with the fit for each $N_{c}$ value ranging from 1 to 3, which we approximate as:

$${BIC}_{N_{c}}=n_{s}\;ln\left(\sum_{i=1}^{n_{s}}\;{\left(I_{i}^{N_{c}}-I_{i}\right)}^{2}/n_{s}\right)+k_{s}\;ln\;n_{s}$$

Where the numerator in the first logarithm is the residual sum of squares, obtained by summing the squared differences between the model fit $I_{i}^{N_{c}}$ (given $N_{c}$ curves) and the experimental peak intensities $I_{i}$ over data points indexed by i spanning the total number of data points for the peptide $\left(n_{s}\right)$, and $k_{s}$ is the total number of model parameters (excluding the final fraction for each timepoint whose value is constrained by normalization). We note that intensities are normalized per-spectrum prior to being plugged into the above calculation so that timepoints with higher ion abundance are not weighted more heavily. We then determine the smallest number of curves $N_{B}$ for which the $\mathrm{BIC}\left(N_{B}\right)$ is greater than the smallest BIC value (across all $N_{c}$ ranging from 1 to 3) by no more than 10 units. As a second method, we compute the goodness of fit for each configuration as:

$$R_{N_{c}}^{2}=1-\frac{\sum_{i=1}^{n_{s}}\;{\left(I_{i}^{N_{c}}-I_{i}\right)}^{2}}{\sum_{i=1}^{n_{s}}\;{\left(I_{i}-\overset{‾}{I}\right)}^{2}}$$

Where $\overset{‾}{I}$ is the average intensity over per-timepoint normalized spectra. From these values, we determine $N_{R}$, which is the smallest value of $N_{c}$ required to achieve $R_{N_{c}}^{2}>0.95$. Finally, the optimal number of curves is chosen as:

$$N_{opt}=min\left\{N_{B},N_{R}\right\}$$

These combined criteria prevent overfitting in a few cases where the BIC calculation favors a larger number of curves than is needed to achieve a satisfactory fit. In the SI, we show plots of selected spectra fit to various numbers of curves with an indication of the optimal number per the above criteria, as well as the mathematical justification. In Fig. S5, we show fits for three representative peptides from aTS to one, two, or three curves as visual evidence that this approach yields a sensible $N_{\text{opt}}$ value. The above criteria are applied to the majority of peptides and overwritten manually in a limited number of cases, primarily where interfering peaks from other peptides are spuriously fit by the algorithm—in this case, the values obtained by these calculations are not reliable. Maximally deuterated samples, in contrast, are fit to just one curve in all cases.

### Bootstrapping and Centroid Comparison

To obtain error estimates on fitting parameters, we bootstrap by resampling global fit residuals (relative to the fit of the initial data) with replacement. Residuals are freely shuffled across spectra from different timepoints and technical replicates (where present) for a given peptide; however, we only allow residual exchange between a given pair of data points if their respective intensities differ by no more than a factor of 5, accounting for the possibility that the noise distribution differs across intensity scales. We also enforce positivity for all resampled intensities.

These resampled data points are then globally re-fit to the same number of curves determined as per the previous section, and the resulting parameters are recorded. This process is repeated 2,000 times for each peptide to obtain distributions and confidence intervals for all parameters, including the number of deuterons for each mode m, computed for each bootstrap iteration as the product $D_{m}=N_{eff}^{m}p_{m}$. We discard any bootstrap iterations for which $D_{m}$ differs from the median value by more than three times the median absolute deviation, as these often represent scenarios where the fit is distorted by spurious peaks due to interfering peptides. We refer to the resulting bootstrapped distribution for the number of deuterons in mode m as $P\left(D_{m}\right)$. Separately, we bootstrap the two maximally deuterated control technical replicates for each sample to obtain a parameter distribution for that condition.

For certain analyses, we compare the deuteration of the least protected (highest $D_{m}$) mode with $D_{m,max}$, the number of deuterons in the unfolded state under the null hypothesis that the former is no smaller than the latter. Extracting $D_{m,u}$ from our maximally deuterated control sample requires correcting for the fact that this sample was prepared via overnight incubation in 90% D_2_O, whereas pulse HDX-MS samples are deuterated for only 10 s. This cannot be trivially corrected experimentally because the presence of 8 M urea slows down the rate of exchange in the base-catalyzed regime (68). At a meter-read pH of 6, we find this leads to incomplete labeling of denatured proteins within 10 s. Instead, we calculate the fraction of deuterons that we expect to have exchanged during our 10 s pulse for each peptide and scale the number of deuterons taken up in our maximally deuterated control by this fraction, as a back exchange correction.

To achieve this, we use a Python implementation of the algorithm in reference (3) to compute the expected exchange rates $k_{i}$ for all backbone amides across the intact protein sequence. These calculations are performed assuming a temperature of 310 K and pH 5.6. For a given peptide spanning residues indexed from a through b, we define the corrected exchange rates as follows:

$${\left\{k_{i}\right\}}_{a\ldots b}=0\;\text{for}\;i=a,a+1\;\;k_{i}=k_{i}\;\text{otherwise}\;$$

where the first two peptide residues have an effective exchange rate of zero because they back-exchange very quickly following digestion (the first residue, in fact, contains a free amine rather than an amide). We then compute the corrected deuteration for the unfolded state as:

$$D_{m,corr}=fD_{m,max}$$

where:

$$f=\frac{1}{b-a-2}\sum_{i=a+2}^{b}\left(1-e^{-k_{i}t}\right)$$

Where t=10s, assuming single-exponential exchange kinetics for each site, and $D_{m,max}$ is the number of deuterons exchanged in the maximally deuterated control. For most peptides, we obtain f values in the range of ~ 0.9 to 1. We likewise apply this correction to the centroid obtained for all bootstrap iterations performed on the maximally deuterated control to obtain a corrected $P\left(D_{m,corr}\right)$ for the expected deuteration of an unfolded polypeptide following 10 s of pulse labeling under our experimental conditions. Finally, we compute the p-value associated with the null hypothesis that $D_{m}\geq D_{m,corr}$ as the fraction of bootstrap trials for which the observed centroid is at least as large as the corrected maximally deuterated centroid as an approximation to:

$$p=\int_{0}^{\infty }dD_{m,corr}P\left(D_{m,corr}\right)\int_{D_{m,corr}}^{\infty }dD_{m}P\left(D_{m}\right)$$

For any peptides for which p<0.01 for the least protected mode and for which $D_{m,corr}-D_{m}\geq 0.5$, we reject the null hypothesis and conclude that the least protected mode is significantly more protected than would be expected if it reflected the unfolded state. This same definition of statistically significant burst-phase protection is also used to designate which peptides are shown with stripped shading in Fig. 2H, with the additional requirement that this partially protected mode show no more than 85% deuterium uptake in this specific panel. For fig. S9E which shows mode deuterations normalized by the maximally deuterated value, the error bars are obtained via element-wise divided samples from the bootstrapped distribution for the respective mode and that for the corrected maximally deuterated control, respectively and then extracting the 95% confidence intervals from the resulting distributions.

We note that, in main text figures showing fractional populations as a function of time and mode protection at each peptide, a subset of high-quality peptides showing poor global fits is excluded. In addition, in cases where multiple charge states exist for a given peptide, the main text figures present only the charge state showing the stronger signal and/or higher quality fitting. In the supplementary figures, we present these graphs with all high-quality peptides and redundant charge states included. We observe reproducible behavior across charge states for a given peptide, even though these are fit and analyzed independently (see supplementary figures).

### End of Translation Determination

As a lower-bound estimate for the first timepoint associated with the end of translation, we considered, for each timepoint t, the most intense amplitude $A_{Cmax,t}$ associated with any peptide/charge state combination spanning the most C-terminal residue for which we have coverage, as well as the least intense amplitude $A_{Nmin,t}$ associated with any peptide spanning the most N-terminal residue for which we have coverage, and computed their ratio, $R_{CN,t}$. We then normalized by this intensity ratio for the exact same peptide/charge state pair at the final translation timepoint, $R_{CN,t_{final}}$, and determined the first timepoint t for which:

$$\frac{R_{CN,t}}{R_{CN,t_{final\;}}}>0.05$$

This threshold implies that enough ribosomes have completed translation that full length protein will appreciably contribute to the detectable mass spectrometric signal at N-terminal peptides. For all proteins, we additionally verified that this timepoint coincides with the appearance of appreciable full-length protein by Western blotting.

## Supplementary Material

Supplement 1

Materials and Methods

Supplementary Text

Figs. S1 to S9

Tables S1 to S4

## Data Availability

All data (raw peptide mass spectra, fitting parameters, unedited western blot images), as well as Python code used to generate the figures in this text, are available on Dryad at https://datadryad.org/share/LINK_NOT_FOR_PUBLICATION/iAhpdnuyJjdeCEWNpFPp52pa9WgKLhZZ8I-9m_axRwI. All DNA constructs are available upon request.
