# Supplementary material for: A detailed molecular picture of protein folding during active translation
Source: bioRxiv. 2026 Aug 10:2026.07.03.736445. Originally published 2026 Jul 4. Preprint. [Version 2] doi: 10.64898/2026.07.03.736445 (PMC13345067; doi:10.64898/2026.07.03.736445)
Supplement: Supplementary file 1 [file NIHPP2026.07.03.736445v2-supplement-1.pdf]

bioRxiv preprint doi: https://doi.org/10.64898/2026.07.03.736445; this version posted August 10, 2026. The copyright holder for this preprint (which was not certified by peer review) is the author/funder, who has granted bioRxiv a license to display the preprint in perpetuity. It is made available under a CC-BY 4.0 International license.

Supplementary text

Supplementary Note 1: Evidence that our method detects folding on the ribosome

The control experiments shown in fig. S1D indicate that nascent chain (NC) binding to streptavidin beads under HDX conditions does not disrupt the integrity of NCs. But this experiment does not rule out detection bias in favor of released proteins resulting from subsequent steps in our sample preparation and injection pipeline, including quench or digestion-related biases. Here, we provide two lines of evidence pointing against such a bias. Together, these strongly indicate that our MS signal faithfully reports on NCs at timepoints when translation is still occurring.

Evidence 1: Mass spectrometric intensities in CMPK are inconsistent with strong bias in favor of released proteins

Let us consider the alternative hypothesis that our mass spectrometric signal is exclusively sensitive to released proteins, namely either nascent chains that are released prematurely or fully synthesized, released protein. For CMPK, we observe that roughly ~70% of ribosomes finish translation, as obtained by quantifying the intensity of the full-length band, relative to the total intensity in the 15 min lane in fig. S4E. Thus, under our **alternative hypothesis, the maximum mass spectrometric signal we will be able to detect from nascent chain peptides at co-translational timepoints will be ~30% that at post-translational times**, assuming 1) negligible variability in total injected material (see next paragraph) and 2) all peptides whose signal is compared have been fully synthesized by 10 mins; this is likely true for N-terminal peptides on which we focus our attention.

In reality, however, we consistently observe that these two conditions show comparable signal at N-terminal peptides (Table S1). For example, for the core peptide 2-24, z=3, the overall intensities (quantified as the total amplitude associated with the global fits at the respective timepoints) at the 10 and 15 min timepoints nearly identical at 1.8E8 and 1.7E8, respectively. The 10 and 15 min intensities are likewise nearly identical for the lid peptide 40-45 z=2, at 1.2E8 and 1.1E8, respectively. We note that this result holds true when corrected for injection-to-injection variability in total amount of injected material. As a proxy this total injected material, we quantify the intensities associated with peptides from two distinct ribosomal proteins, L5 and L23, as detected in these specific samples (although our biotin-streptavidin pulldown strategy appreciably enriches for the NC, some signal from ribosomal proteins is nonetheless detected due to nonspecific binding to streptavidin beads and/or co-association with NCs stemming from incomplete ribosomal dissociation during quench).

Although these analyses reveal that the amount of material in the 10 mins sample is slightly greater than that in the 15 mins sample by a factor of 1.5 using the proxy ribosomal protein L5 (Table S2) and a factor of 1.4 when using the proxy protein L23 (Table S3), these ratios are not sufficient to support the alternative hypothesis; the total injected material in the 10 min timepoint would have to be at least ~3.3 times that in the 15 min timepoint in order for the two timepoints

bioRxiv preprint doi: https://doi.org/10.64898/2026.07.03.736445; this version posted August 10, 2026. The copyright holder for this preprint (which was not certified by peer review) is the author/funder, who has granted bioRxiv a license to display the preprint in perpetuity. It is made available under a CC-BY 4.0 International license.

to show similar N-terminal CMPK intensities under the hypothesis that no more than 30% of the population (i.e. the spuriously released NCs) is detectable at the 10 min timepoint.

We additionally note that for lid peptide 40-45, ~60% of this signal at 10 min is attributed to the protected mode, and the remaining 40% is attributed to a burst phase mode with significant protection (Fig. 2C and fig. S4). Let us consider the related hypothesis that this ~60% detectable protected population consists entirely of spuriously released NCs, which comprise up to 30% of the population. Then this would require that the total amount of injected material associated with the 10 mins timepoint be at least ~2x associated with the 15 mins sample, again larger than the ~1.4x-1.5x factor observed. **This strongly supports that our method can detect co-translational folding in actively translating nascent chains.**

We note that over long timescales (~30 min to hours), the signal at all peptides, including N-terminal peptides steadily increases, which we attribute to multiphasic biotinylation kinetics (fig. S1G).

Evidence 2: Mass spectrometric signal is unlikely to be skewed by nascent-chain-specific precipitation during HDX quench

As a second line of reasoning, we explicitly consider what we believe to be the most likely mechanistic origin for any possible detection bias against nascent chains: namely, selective precipitation of peptidyl-tRNA attached NCs during low-pH quench conditions *(52)*, which could inhibit digestion of these species while potentially leaving released proteins accessible to digestion. As our quench step is applied to NCs that are physically attached to micron-sized streptavidin beads, we cannot simply perform a centrifugation-based pelleting assay to quantify the amount of precipitated NCs under our conditions. Instead, we address this hypothesis by systematically increasing the concentration of GdmCl in our quench buffer from 2M (the concentration used in most of our experiments) to 6M which should significantly reduce precipitation. Thus, if the signal under our standard 2M quench is heavily biased by aggregates, then this [GdmCl] increase should notably alter the mass spectra we observe. We note that in both cases we reduce the [GdmCl] to 2M while adding pepsin to ensure the protease can efficiently digest the sample—this complete buffer exchange is uniquely enabled by our bead setup.

Contrary to this expectation, we observe highly similar mass spectra for the model protein aTS between two simultaneously-performed pulse-labeling HDX replicates after 8 mins of synchronized elongation—these replicates differ only in their concentration of GdmCl in the quench as described above. For example, under both quench conditions, peptides 1-7 and 128-152 show no protection at 8 mins, whereas peptides 35-40 and 85-101 show partial protection. As controls, we also confirm that the 60 mins (post-translational) and maximally deuterated conditions also show similar spectra across both quenches with slightly increased back-exchange when 6M GdmCl is used. These results strongly suggest that our signal is not notably biased against NCs due to acid-induced precipitation under our standard quench conditions.

We note that, formally, we cannot rule out the possibility that some on-bead aggregation occurs amongst bead-attached NCs in our 6M sample following buffer-exchange into the 2M buffer to enable digestion. However, this is extremely unlikely under single-turnover translation

bioRxiv preprint doi: https://doi.org/10.64898/2026.07.03.736445; this version posted August 10, 2026. The copyright holder for this preprint (which was not certified by peer review) is the author/funder, who has granted bioRxiv a license to display the preprint in perpetuity. It is made available under a CC-BY 4.0 International license.

conditions where the maximal NC concentration equals the ribosome concentration, which is about 2 μM in PURE systems *(46)*. We estimate that under our experimental conditions assuming this upper-bound concentration, the average spacing between NCs on the beads would be about 16 nm. This spatial isolation strictly constraints macro-aggregation, limiting any possible interactions to low-order oligomerization at most. Moreover, the concentration of NCs is almost certainly much less than 2 μM in our experiments, as per our observation that the total intensity due to translated protein on a Coomassie-stained gel is significantly less than that associated with ribosomal proteins (see fig. S7C for such a gel on HaloTag).

	**2-21, z2**	**2-24, z3**	**2-26, z3**	**27-39, z2**	**40-45, z2**	**46-67, z4**
**5mins**	3.85E+07	5.22E+07			1.91E+07	
**6mins**	2.31E+07	6.31E+07	1.01E+07		2.59E+07	1.46E+07
**7mins**	7.88E+07	1.18E+08	1.64E+07	2.93E+07	7.33E+07	1.53E+07
**8mins**	6.89E+07	1.20E+08	1.44E+07	2.42E+07	7.70E+07	2.66E+07
**10mins**	7.79E+07	1.79E+08	3.14E+07	3.82E+07	1.19E+08	5.96E+07
**15mins**	8.88E+07	1.73E+08	3.11E+07	3.61E+07	1.05E+08	6.16E+07
**30mins**	1.97E+08	2.81E+08	5.81E+07	7.81E+07	2.64E+08	6.10E+07
**2hrs**	4.27E+08	6.56E+08	1.09E+08	1.49E+08	5.60E+08	1.90E+08

	**58-67, z2**	**68-78, z2**	**120-126, z2**	**127-147, z3**	**136-147, z2**	**148-162, z4**
**5mins**	1.52E+07					
**6mins**	1.93E+07	7.17E+06				
**7mins**	1.10E+08		1.03E+07	3.46E+06		
**8mins**	1.18E+08	1.48E+07	2.45E+07	2.03E+07	2.04E+07	
**10mins**	1.65E+08	3.25E+07	7.40E+07	9.84E+07	1.09E+08	5.66E+06
**15mins**	1.69E+08	3.82E+07	9.68E+07	1.99E+08	1.92E+08	2.52E+07
**30mins**	4.58E+08	5.42E+07	2.71E+08	3.78E+08	6.80E+08	2.95E+07
**2hrs**	1.07E+09	1.52E+08	8.21E+08	7.74E+08	1.90E+09	1.19E+08

	**175-200, z5**	**207-218, z2**	**208-218, z2**	**211-218, z2**
**5mins**				
**6mins**				
**7mins**				
**8mins**				
**10mins**	2.04E+06		1.72E+06	
**15mins**	3.40E+07		3.08E+07	6.49E+06

bioRxiv preprint doi: https://doi.org/10.64898/2026.07.03.736445; this version posted August 10, 2026. The copyright holder for this preprint (which was not certified by peer review) is the author/funder, who has granted bioRxiv a license to display the preprint in perpetuity. It is made available under a CC-BY 4.0 International license.

**30mins**	6.81E+07	1.93E+07	1.17E+08	4.04E+07
**2hrs**	2.24E+08	7.40E+07	4.19E+08	1.44E+08

Table S1.
Global fit amplitude, a quantification of overall mass spectrometric intensity, as a function of translation time for spectra corresponding to indicated CMPK peptide and charge state combinations. No value indicates the peptide was not detected at the indicated time

	**20-36, z3**	**37-57, z3**	**139-153, z4**	**139-153, z3**	**171-179, z3**
**8mins**	6.80E+08	4.93E+09	1.51E+09	7.77E+08	1.43E+09
**10mins**	5.40E+08	4.24E+09	1.57E+09	7.45E+08	1.48E+09
**15mins**	4.29E+08	3.31E+09	9.37E+08	4.61E+08	9.88E+08
**30mins**	4.29E+08	3.10E+09	7.49E+08	4.04E+08	9.08E+08

**Average 10min to 15min ratio**	**1.46585922**

Table S2.
Global fit amplitude, a quantification of overall mass spectrometric intensity, as a function of translation time for spectra corresponding to indicated peptide and charge state combinations from ribosomal protein L5 during translation reaction of CMPK. Only a subset of translation times is shown. Final row shows average ratio of intensity at 10 mins to that at 15 mins over all peptides.

	**1-7, z2**	**1-7, z3**	**8-29, z4**	**8-29, z5**	**30-50, z5**	**33-50, z4**	**33-50, z5**
**8mins**	6.30E+07	6.30E+07	8.96E+07	1.03E+08	7.84E+07	6.80E+07	4.17E+07
**10mins**	6.20E+07	7.17E+07	9.13E+07	1.02E+08	8.88E+07	7.60E+07	6.32E+07
**15mins**	5.74E+07	5.87E+07	7.19E+07	6.17E+07	7.73E+07	5.19E+07	3.16E+07
**30mins**		6.85E+07	4.76E+07	6.08E+07	3.79E+07	5.01E+07	3.99E+07

bioRxiv preprint doi: https://doi.org/10.64898/2026.07.03.736445; this version posted August 10, 2026. The copyright holder for this preprint (which was not certified by peer review) is the author/funder, who has granted bioRxiv a license to display the preprint in perpetuity. It is made available under a CC-BY 4.0 International license.

**Average 10min to 15min ratio**	**1.40545405**

Table S3.
Global fit amplitude, a quantification of overall mass spectrometric intensity, as a function of translation time for spectra corresponding to indicated peptide and charge state combinations from ribosomal protein L23 during translation reaction of CMPK. Only a subset of translation times are shown. Final row shows average ratio of intensity at 10 mins to that at 15 mins over all peptides.

Supplementary Note 2: Reproducibility analysis

We analyze reproducibility in the order in which peptides become protected across biological replicates of our translation reactions by generating scatter plots showing the natively protected fractional population at a given timepoint in both replicates and computing the spearman correlation. These analyses are conducted at timepoints at which a subset of peptides has begun to show protection, while others do not, so as to most cleanly delineate the sequence of acquisition of protection. These plots are shown in fig. S4I-J, fig. S6H, and fig. S7H-I for CMPK, aTS, and HaloTag, respectively at the indicated timepoints. These analyses all show high Spearman correlation coefficients above 0.8 with associated p values less than 10^{-5}, except for CMPK which has a reduced peptide coverage relative to the other proteins and p-values of approximately 10^{-4} to10^{-3}. The following factors contribute to the slight remaining variability between replicates:

1. As the two replicates are fit independently, the centroids associated with the relevant modes may vary slightly between replicates. As a result, the associated population fractions at a given timepoint may vary. This is especially applicable for peptides exhibiting closely overlapping modes. This may be further confounded by the fact that, in some replicate reactions, certain timepoint were not collected.
2. Variations in translation speed imply that, at a given timepoint, different replicates may have progressed to different degrees in the folding reaction. This will lead to noise in the fitted population fractions, particularly when the fractions are close to 0 or 1 in one replicate, but not the other.

The observation of high and statistically significant correlation coefficients despite these limitations in the analysis strongly indicates a high degree of reproducibility.

Notably, applying this comparison of HaloTag co-translational folding and refolding at comparable timepoints did not yield a meaningful correlation (R=-0.21, p=0.55, fig. S8E), supporting that these proceed through different folding pathways.

bioRxiv preprint doi: https://doi.org/10.64898/2026.07.03.736445; this version posted August 10, 2026. The copyright holder for this preprint (which was not certified by peer review) is the author/funder, who has granted bioRxiv a license to display the preprint in perpetuity. It is made available under a CC-BY 4.0 International license.

Supplementary note 3: Altered HaloTag folding sequence cannot be accounted for by lagging ribosomes

We note that peptides from N-terminal regions will generally appear to fold more slowly in our assay than peptides from the C-terminus, even if the regions fold simultaneously in reality, as lagging ribosomes will present as a population of unfolded N-terminal peptides that is slow to vanish. However, this effect is not sufficient to account for our observation that newly-translated HaloTag folds starting from the C terminus, in contrast to refolding from denaturant where initial folding involves both termini. To show this, we consider the null hypothesis in which the folding sequence of newly-synthesized HaloTag is precisely the same as that observed during refolding, and show that even after accounting for the apparent delay in N-terminal folding due to lagging ribosomes, this model cannot explain our observed results .

To do this, we develop an analytical null model under minimal assumptions and set upper bounds on the ratio of the probability that C-terminal peptides appear folded to the probability that N-terminal peptides appear folded at various times under our model. We show that the upper bound on this ratio under our null model is lower than many of the values we observe.

Null model assumptions

1. We assume native folding of all regions in a given HaloTag molecule does not begin until that molecule is fully synthesized and released from the ribosome, based on our observation of no native-like co-translational folding.

2. We let f denote the fraction of ribosomes that successfully finish translation, conditional on them having translated through the N-terminal residues of interest, and assume that the remaining fraction $1 - f$ of aborted nascent chains do not show any folding of residues that have been synthesized. In reality, our stalled RNC experiments demonstrate that these may fold given enough time, but allowing for this will only decrease the worst-case upper bound that our null model predicts for the C-terminal to N-terminal folded ratio.

bioRxiv preprint doi: https://doi.org/10.64898/2026.07.03.736445; this version posted August 10, 2026. The copyright holder for this preprint (which was not certified by peer review) is the author/funder, who has granted bioRxiv a license to display the preprint in perpetuity. It is made available under a CC-BY 4.0 International license.

3. While folding of neither N- nor C-terminal regions is permitted until after the C-terminus is complete, we assume that N-terminal peptides can be *detected* (in the unfolded state) immediately upon their translation.

1 The model

We define the following general functions:

- $g_N(t)$: The probability density function that a ribosome finishes translating the N-terminal residues of interest at time t.
- $g_C(t)$: The probability density function that a ribosome finishes translating the full protein at time t. Note that $g_C(t) \leq g_N(t)$ for all t.
- $f_N(t)$: The probability that an N-terminal region of interest is folded in a given HaloTag molecule some time t after that molecule's translation is complete.
- $f_C(t)$: The probability that a C-terminal region of interest is folded in a given HaloTag molecule some time t after that molecule's translation is complete.

To begin, we consider pairs of N- and C-terminal regions where the former folds more rapidly than the latter during refolding from denaturant. Thus under our null assumption, $f_N(t) \geq f_C(t)$ for all t.

Under these assumptions, the observed probability that an N-terminal region and a C-terminal region of interest appear folded at time t are given by, respectively:

$$P_{obs,N}(t) = \frac{f \int_{-\infty}^{t} d\tau g_C(\tau) f_N(t-\tau)}{\int_{-\infty}^{t} d\tau g_N(\tau)} \tag{1}$$

$$P_{obs,C}(t) = \frac{\int_{-\infty}^{t} d\tau g_C(\tau) f_C(t-\tau)}{\int_{-\infty}^{t} d\tau g_C(\tau)} \tag{2}$$

Using $f_N(t) \geq f_C(t)$, we then have:

$$\frac{P_{obs,C}(t)}{P_{obs,N}(t)} \leq \frac{1}{f} \frac{\int_{-\infty}^{t} d\tau g_N(\tau)}{\int_{-\infty}^{t} d\tau g_C(\tau)} \tag{3}$$

bioRxiv preprint doi: https://doi.org/10.64898/2026.07.03.736445; this version posted August 10, 2026. The copyright holder for this preprint (which was not certified by peer review) is the author/funder, who has granted bioRxiv a license to display the preprint in perpetuity. It is made available under a CC-BY 4.0 International license.

Let us further assume that all ribosomes complete synthesis of the N-terminal region before any ribosome finishes translation of the full protein, in which case the integral in the numerator of Equation 3 is 1 for all t of interest. Although this assumption may not be true in general, making this assumption allows us to set the strictest possible upper bound on $P_{obs,C}(t)/P_{obs,N}(t)$. In this case, we have:

$$\frac{P_{obs,C}(t)}{P_{obs,N}(t)} \leq \frac{1}{f} \frac{1}{\int_{-\infty}^{t} d\tau g_C(\tau)} \tag{4}$$

Or inverting, we have

$$\frac{P_{obs,N}(t)}{P_{obs,C}(t)} \geq f \int_{-\infty}^{t} d\tau g_C(\tau) \tag{5}$$

In other words, the observed ratio of the N-terminal folded fraction to the C-terminal folded fraction must be at least as large as the total fraction of ribosomes that has finished translated at time t.

Null model predictions applied to experiment

We now show that $P_{obs,N}(t)/P_{obs,V}(t)$ is smaller than allowed by Equation 5 at various timepoints t, in violation of our null model. In all cases we use a value of $f = 0.7$, under the assumption that all protein regions that have not folded at the final (240 min) timepoint come from aborted ribosomes—this yields an aborted fraction of 0.3. In reality, this assumption may be too strict as this unfolded fraction may also include a subset of fully synthesized molecules that fails to fold. We further reiterate that this bound reflects the extreme scenario where N and C termini fold simultaneously. In the following section ("Null model under additional assumptions"), we explicitly vary the ratio of folding rates and, under additional assumptions, can obtain a looser bound.

20 minute timepoint

At this time, the intensity ratio of the most intense C-terminal peptide to the least intense N-terminal peptide, normalized by this same ratio at the final timepoint, is ~ 0.65, indicating that approximately this fraction has finished translating. Notably, quantification of the 20 min lane in the western blot in Fig. S7B gives an even higher fraction of 0.8 (which itself may be an underestimate due to saturation of the full-length band). Using the more conservative value of 0.65, Equation 5 gives:

$$\frac{P_{obs,N}(t)}{P_{obs,C}(t)} \geq 0.65 \times f \approx 0.45 \tag{6}$$

bioRxiv preprint doi: https://doi.org/10.64898/2026.07.03.736445; this version posted August 10, 2026. The copyright holder for this preprint (which was not certified by peer review) is the author/funder, who has granted bioRxiv a license to display the preprint in perpetuity. It is made available under a CC-BY 4.0 International license.

Again using $f = 0.7$. At the 20 min timepoint, the C-terminal peptide 274–285 is already 100% folded, so all regions that fold more rapidly than this C-terminal region during refolding must be at least 45% folded at this time under our null model. But as shown in Fig. 3C, many such peptides are notably less folded than this. For example, 19–36 is only about 30% folded. Peptide 2–18 is less than 20% folded—this is true of both charge states detected for this peptide (Fig. S7). Finally, peptide 81–87 is only about 35% folded (We note that during refolding, this region is reported on by an analogous peptide 81–90).

Similarly, at 20 mins, peptide 252–264 is approximately 65% folded. Thus, all peptides that fold more rapidly than 252–264 during refolding must be at least 29.5% folded per our null model. But this is again violated by 2–18.

We note that even in the unlikely case that both our methods for quantifying the amount of fully translated protein yielded a significant overestimate and the true fully translated fraction were as low as $\sim 30\%$ (a highly unrealistic scenario given the substantial intensity for full length band on western blot and the high intensity for C-terminal peptides that matches expectation from the final timepoint assuming synthesis is complete), then the observed folded fraction for peptide 2–18 would still be too low in comparison with 274–285 to be accounted for by our null model, which would set a bound at 21% in this extreme case.

60 minute timepoint

At this time, the intensity ratio of the most intense C-terminal peptide to the least intense N-terminal peptide, normalized by this same ratio at the final timepoint, is ~ 1.14 times that at the final (240 min) timepoint, a strong indication that essentially all ribosomes have finished translation. At these times, the integral in the denominator of Equation 4 is simply 1, and so we have:

$$\frac{P_{obs,C}(t)}{P_{obs,N}(t)} \leq \frac{1}{f} \approx 1.43 \tag{7}$$

But as shown in Fig. 3C, if we consider the C-terminal peptide 274–285 and the N-terminal peptide 2–18, we observe a $P_{obs,C}(t)/P_{obs,N}(t)$ ratio of approximately 2 at 60 mins, notably higher than this bound, despite the fact that during refolding, 2–18 folds more rapidly.

A note on 274–285 vs 286–293

Let us now consider peptide 274–285 as the "N-terminal peptide" and 286–293 as the "C-terminal peptide". We note that at all timepoints prior to the end of refolding, the analogous 286–294 peptide is *more* folded than 274–285, so for this peptide pair, the inequality in Equation 3 flips:

bioRxiv preprint doi: https://doi.org/10.64898/2026.07.03.736445; this version posted August 10, 2026. The copyright holder for this preprint (which was not certified by peer review) is the author/funder, who has granted bioRxiv a license to display the preprint in perpetuity. It is made available under a CC-BY 4.0 International license.

$$\frac{P_{obs,C}(t)}{P_{obs,N}(t)} > \frac{1}{f}\frac{\int_{-\infty}^{t} d\tau g_N(\tau)}{\int_{-\infty}^{t} d\tau g_C(\tau)} \tag{8}$$

Since these peptides are adjacent, it is reasonable to assume that few ribosomes stall between them, so it is likely that $f \approx 1$. Moreover, using the fact that the numerator in Equation 8 is greater than the denominator, we would thus expect:

$$\frac{P_{obs,C}(t)}{P_{obs,N}(t)} > 1 \tag{9}$$

In reality, the expected lower bound would be much higher than this since 274–285 folds significantly more slowly than 286–293 during refolding. But even neglecting this, at all timepoints in our translation experiment, this ratio is actually 1, in violation of our null model.

Null model under additional assumptions

Under a few additional reasonable assumptions, we can set even more strict bounds on the ratio of folded fractions that is expected under the null model at times shortly after the first ribosomes have completed synthesis. At these early times, for example, the 15 mins translation timepoint in the main-text replicate, it is reasonable to assume single-exponential folding kinetics, as the folding times will be much shorter than the characteristic timescales associated with any slower exponential rates in a multi-exponential process. Thus,

$$f_{N/C}(t) = 1 - e^{-k_{N/C}t} \tag{10}$$

where $k_{N/C}$ denote rate constants for N and C terminal regions, respectively. For analytical tractability, we further assume a uniform distribution of synthesis-completion times of width τ_f. That is:

$$g_C(t) = \begin{cases} 1/\tau_f & \text{if } 0 \le t \le \tau_f \\ 0 & \text{otherwise} \end{cases} \tag{11}$$

Under these conditions, we have:

$$\frac{P_{obs,C}(t)}{P_{obs,N}(t)} = \frac{\tau_f}{f\ t}\frac{\int_{0}^{t} d\tau(1 - e^{-k_C(t-\tau)})}{\int_{0}^{t} d\tau(1 - e^{-k_N(t-\tau)})} \tag{12}$$

for $0 \le t \le \tau_f$. The integrals can be evluated and the result expressed in terms of dimensionless quantities:

bioRxiv preprint doi: https://doi.org/10.64898/2026.07.03.736445; this version posted August 10, 2026. The copyright holder for this preprint (which was not certified by peer review) is the author/funder, who has granted bioRxiv a license to display the preprint in perpetuity. It is made available under a CC-BY 4.0 International license.

$$\frac{P_{obs,C}(t)}{P_{obs,N}(t)} = \frac{1}{f} \frac{\left[1 - \frac{1}{k_N t}\left(\frac{k_C}{k_N}\right)^{-1}\left(1 - e^{-\left(\frac{k_C}{k_N}\right)k_N t}\right)\right]}{\frac{t}{\tau_f} - \frac{(t/\tau_f)}{k_N t}\left(1 - e^{-k_N t}\right)} \tag{13}$$

Plots of Equation 13 are shown in Fig. S7J as a function of k_C/k_N. We expect a value of approximately 0.1 from Dall et al., PNAS 2024. We further note that at 15 mins, the intensity ratio of the most intense C-terminal peptide to the least intense N-terminal peptide, normalized to this same quantity at the final timepoint, is roughly 0.4, so we set $t/\tau_f = 0.4$, although we consider a range of values. Finally, we note that in our translation experiment, N-terminal peptides fold at timescales slower than 30 mins, and that at the 15 min timepoint, the fastest ribosome must have finished translation less than 5 minutes ago (since no C-terminal peptides were observed at the 10 min timepoint). We thus insist that $k_N t < 5/30 \approx 0.17$. We use this value as an upper bound. Using values lower than this does not impact the result (Fig. S7K).

We find in Fig. S7J that over a range of k_C/k_N values around 0.1, $P_{obs,C}(t)/P_{obs,N}(t)$ is no larger than ~ 1 in our null model for a wide range of t/τ_f values above ~ 0.15—we note that values lower than this are unreasonable anyways since the mass spectrometric intensity of C-terminal peptides and the intensity of the full-length western blot band at 15 mins are both inconsistent with only 15% having finished translation at this time. This is starkly inconsistent with the observation that, at 15 mins of translation, C-terminal peptides show a folded fraction $\sim 2-5$ times larger than N-terminal peptides that fold significantly faster than the respective C-terminal regions during refolding from denaturant.

bioRxiv preprint doi: https://doi.org/10.64898/2026.07.03.736445; this version posted August 10, 2026. The copyright holder for this preprint (which was not certified by peer review) is the author/funder, who has granted bioRxiv a license to display the preprint in perpetuity. It is made available under a CC-BY 4.0 International license.

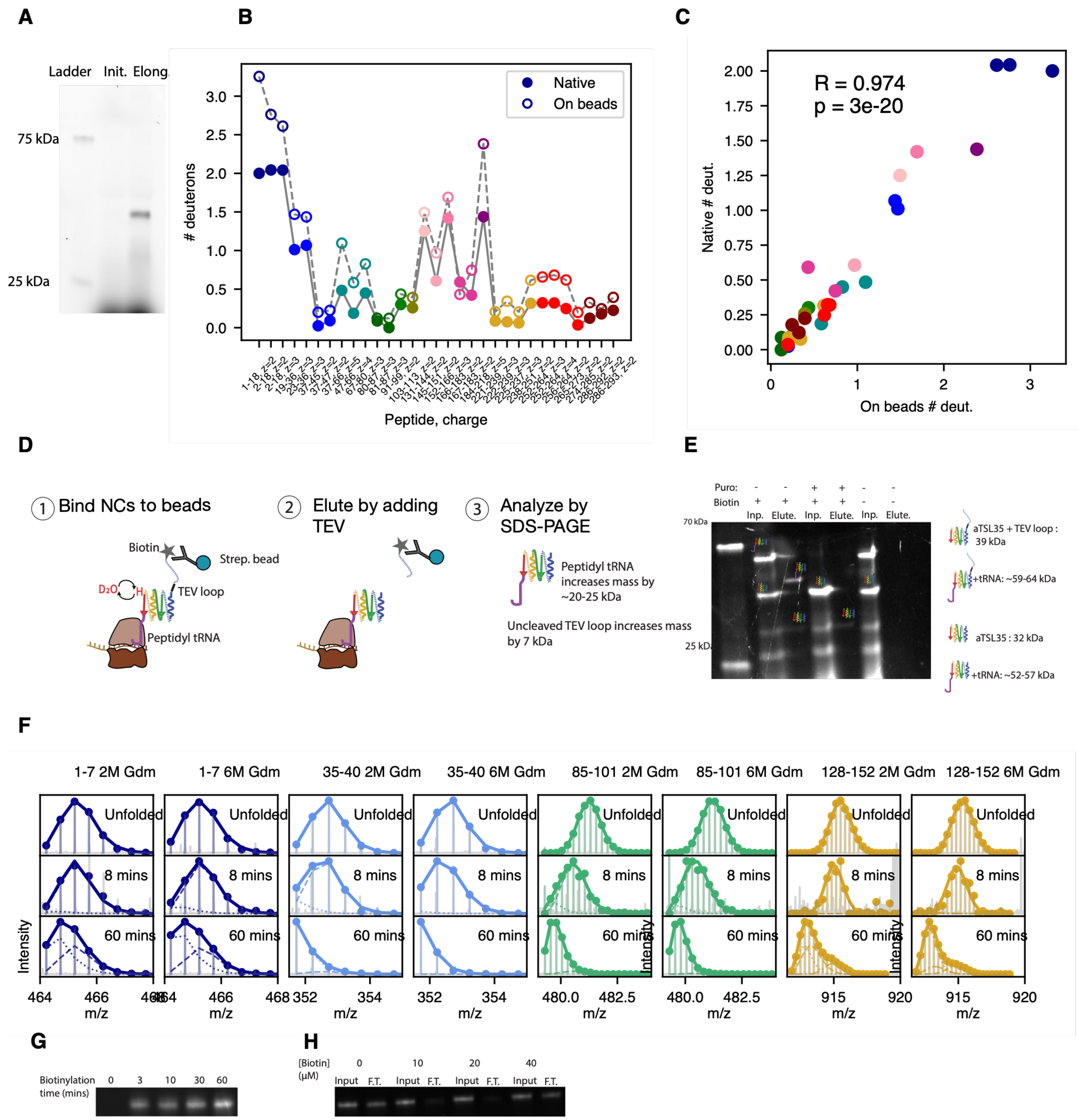

bioRxiv preprint doi: https://doi.org/10.64898/2026.07.03.736445; this version posted August 10, 2026. The copyright holder for this preprint (which was not certified by peer review) is the author/funder, who has granted bioRxiv a license to display the preprint in perpetuity. It is made available under a CC-BY 4.0 International license.

fig. S1. Validation of single-turnover translation and HDX-MS enrichment protocols

(A) Addition of 75 μM aurintricarboxylic acid (ATCA) at the elongation step leads to single-turnover translation. A synchronized translation reaction of aTS with Fluorotect GreenLys was treated with ATCA at either the initiation or elongation step, resolved via SDS-PAGE, and visualized by fluorescent imaging. Full-length aTS product is observed only when ATCA is added at the elongation step (right lane, "Elong.") but not at the initiation step (left lane, "Init."), indicating that ATCA blocks translation initiation without inhibiting actively elongating ribosomes. **(B)** Nascent protein enrichment coupled to HDX-MS does not globally disrupt protein structure. Pulse HDX-MS was performed on a HaloTag sample after 4 hours of elongation, enriched via biotin/streptavidin affinity capture. The number of deuterons accumulated per peptide in the protected mode (empty markers) is compared to a standard 10-second pulse-labeled HDX control on purified HaloTag protein (filled markers) in the same pH 6 buffer. **(C)** Correlation between peptide deuteration levels for the two experiments shown in (B). The high Pearson correlation coefficient and low p-value indicate a strong linear correlation. The slope of less than 1 indicates that deuterium uptake under enrichment conditions is systematically increased across the protein by a constant factor, suggesting that while the enrichment protocol (biotinylation and streptavidin bead binding) slightly destabilizes the protein and/or affects the extent of back exchange, it does not globally alter its folded structure. **(D)** Schematic of protocol for assessing nascent chain (NC) integrity following streptavidin bead binding. We translate a stalled NC construct with an N-terminal AviTag separated from the protein of interest by a linker containing a TEV loop. Following hydrogen-deuterium exchange and bead binding, TEV protease is added to elute NCs from beads, and samples are analyzed by SDS PAGE. If NCs are attached to the ribosome upon elution, then they will migrate ~20-25 kDa larger than expected due to peptidyl tRNA attachment, a migration shift that vanishes if NCs are deliberately released from the ribosome via puromycin addition. For details, see Materials and Methods. **(E)** SDS-PAGE gel, imaged via fluorescence of NCs containing FluorTect GreenLys (see Methods), showing input (prior to bead binding) and elution (post TEV addition) samples as described in (D). Puromycin is added prior to bead binding, and biotin is included in IVT reactions, as indicated. We observe elutants migrate ~20-25 kDa larger in the absence of puromycin than in its presence, indicating peptidyl tRNA attachment, so long as biotin is added to the translation reaction. The absence of biotin precludes streptavidin bead binding, and thus no elutants are observed. We note that elutants migrate 7 kDa faster than their respective input samples due to loss of N-terminal linker following TEV loop cleavage. **(E)** Mass spectra (represented as described in main text Fig. 2B) of various aTS-derived peptides derived from maximally deuterated control (“Unfolded”), 8 mins translation timepoint, and 60 mins translation timepoint following pulse-labeling HDX and bead binding reaction quenched with low pH buffer containing either 2M or 6M GdmCl. The high degree of similarity between trends observed in the spectra from these two conditions both during mid-translation (8 mins) and post-translation (6 mins) strongly suggests against the possibility that the observed mass spectrometric signal in our active translation experiment is biased towards spuriously released NCs due to acid-induced precipitation of peptidyl-tRNA attached species. If such precipitation were to occur, then increasing [GdmCl] from 2 to 6M would significantly reduce its extent and thus affect the observed signal. We note that the 6M GdmCl quench leads to slightly increased back exchange

bioRxiv preprint doi: https://doi.org/10.64898/2026.07.03.736445; this version posted August 10, 2026. The copyright holder for this preprint (which was not certified by peer review) is the author/funder, who has granted bioRxiv a license to display the preprint in perpetuity. It is made available under a CC-BY 4.0 International license.

as observed in respective maximally deuterated control samples. (**F)** Determination of time required to biotinylate nascent chains: The protein HaloTag with an N-terminal AviTag and C-terminal 35 AA linker was translated *in vitro* in the presence of 20 μM biotin for 1.5 hours, then was supplemented with 75 μM ATCA (to prevent further initiation) and 1 μM BirA to begin biotinylation. At indicated times (including t=0, immediately prior to addition of BirA), a 4 μL reaction aliquot was drawn and translation was quenched with 18 μM puromycin at room temperature for 30 seconds, and samples were then mixed with Lamelli dye at 1X final concentration and immediately incubated at 95C for 5 mins to fully inactive BirA. All samples were then loaded on an SDS-PAGE gel and transferred to a PVDF membrane for anti-biotin western blotting with an Alexa-488 conjugated streptavidin probe. We conclude that nascent chain biotinylation is multiphasic, with a substantial degree of biotinylation occurring within a few minutes, indicating it is possible to appreciably enrich for nascent chains at early translation times. However, complete biotinylation requires hour timescales (H) Determination of optimal biotin concentration for nascent chain binding to streptavidin beads: The protein HaloTag with an N-terminal AviTag and C-terminal 35 AA linker was translated *in vitro* for 1 hour at 37C in the presence FluoroTect and variable biotin concentrations as indicated. Subsequently, 2 μL of each sample was drawn as an "input sample", diluted in HKMT for a total volume of 8 μL, and frozen, then the remaining volume was bound for 2 hours with rotary mixing to a 15 μL aliquot of Pierce Streptavidin beads pre-equilibrated in HKMT. The flow-through was removed, and 8 μL of flow-through (F.T.) was drawn for gel analysis—we note that, if we assume no bead binding, these 8 μL aliquots would contain the same quantity of nascent chains as the input sample. All 8 μL samples were then treated with RNAse A to 1 mg/ml, followed by addition of Laemmli buffer to 1X concentration. Samples were then analyzed on a NUPAGE 4-12% Bis Tris gel and FluoroTect fluorescence was imaged. We conclude the 20 μM biotin corresponds to the optimal concentration for bead binding—increasing to 40 μM leads to reduced bead binding due to free biotin competing with streptavidin binding sites.

bioRxiv preprint doi: https://doi.org/10.64898/2026.07.03.736445; this version posted August 10, 2026. The copyright holder for this preprint (which was not certified by peer review) is the author/funder, who has granted bioRxiv a license to display the preprint in perpetuity. It is made available under a CC-BY 4.0 International license.

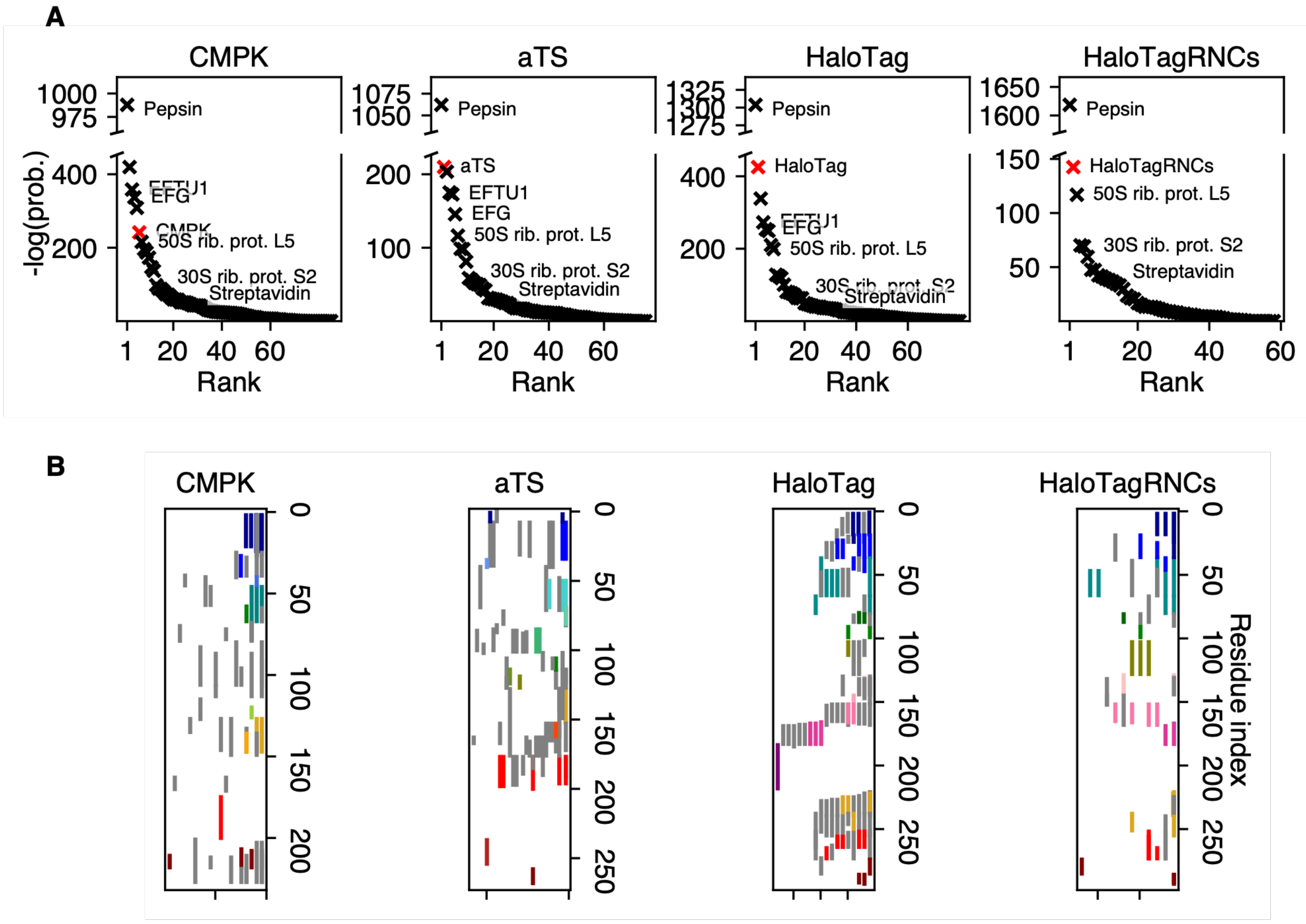

fig. S2. Nascent chain enrichment and coverage

(A) For each protein of interest that we analyze in this work (indicated above respective plots), we show confidence associated with identification of various protein species derived from our *in vitro* translation mixture, as well as proteins from our enrichment + digestion pipeline. Protein identification confidences are ranked by negative log p-values, with higher values indicating a lower probability of false assignment by the Byonic search algorithm. In each plot, the red marker indicates the protein of interest, while other proteins are shown in black. The positions corresponding to various proteins are annotated including pepsin (used for digestion), elongation factors EFTU1 and EFG, two ribosomal proteins L5 and S2, as well as streptavidin. For all proteins except CMPK, the protein of interest produces the highest confidence identification other than pepsin, which is added to bead slurry at high levels for digestion. The score corresponding to CMPK is slightly lower due to a lower digestion efficiency for this protein, but nonetheless high compared to the majority of proteins that were originally present in the non-enriched sample. HaloTagRNCs refers to a HaloTag + 34 AA C-terminal linker containing a strong SecM stalling sequence purified by sucrose cushion ultracentrifugation, which de-enriches

bioRxiv preprint doi: https://doi.org/10.64898/2026.07.03.736445; this version posted August 10, 2026. The copyright holder for this preprint (which was not certified by peer review) is the author/funder, who has granted bioRxiv a license to display the preprint in perpetuity. It is made available under a CC-BY 4.0 International license.

non-ribosomal proteins from this sample. **(B)** Coverage maps indicating high confidence peptides detected from the indicated proteins. Each vertical bar represents a unique peptide + charge state combination that spans the residues indicated on the right axis provided its corresponding Pep2D value (a confidence metric used by Byonic) is less than 0.1, and colored bars represent high-quality peptides used for HDX-MS analysis. We note that these figures show representative coverage maps from a single replicate; the exact coverage varies slightly from replicate to replicate.

bioRxiv preprint doi: https://doi.org/10.64898/2026.07.03.736445; this version posted August 10, 2026. The copyright holder for this preprint (which was not certified by peer review) is the author/funder, who has granted bioRxiv a license to display the preprint in perpetuity. It is made available under a CC-BY 4.0 International license.

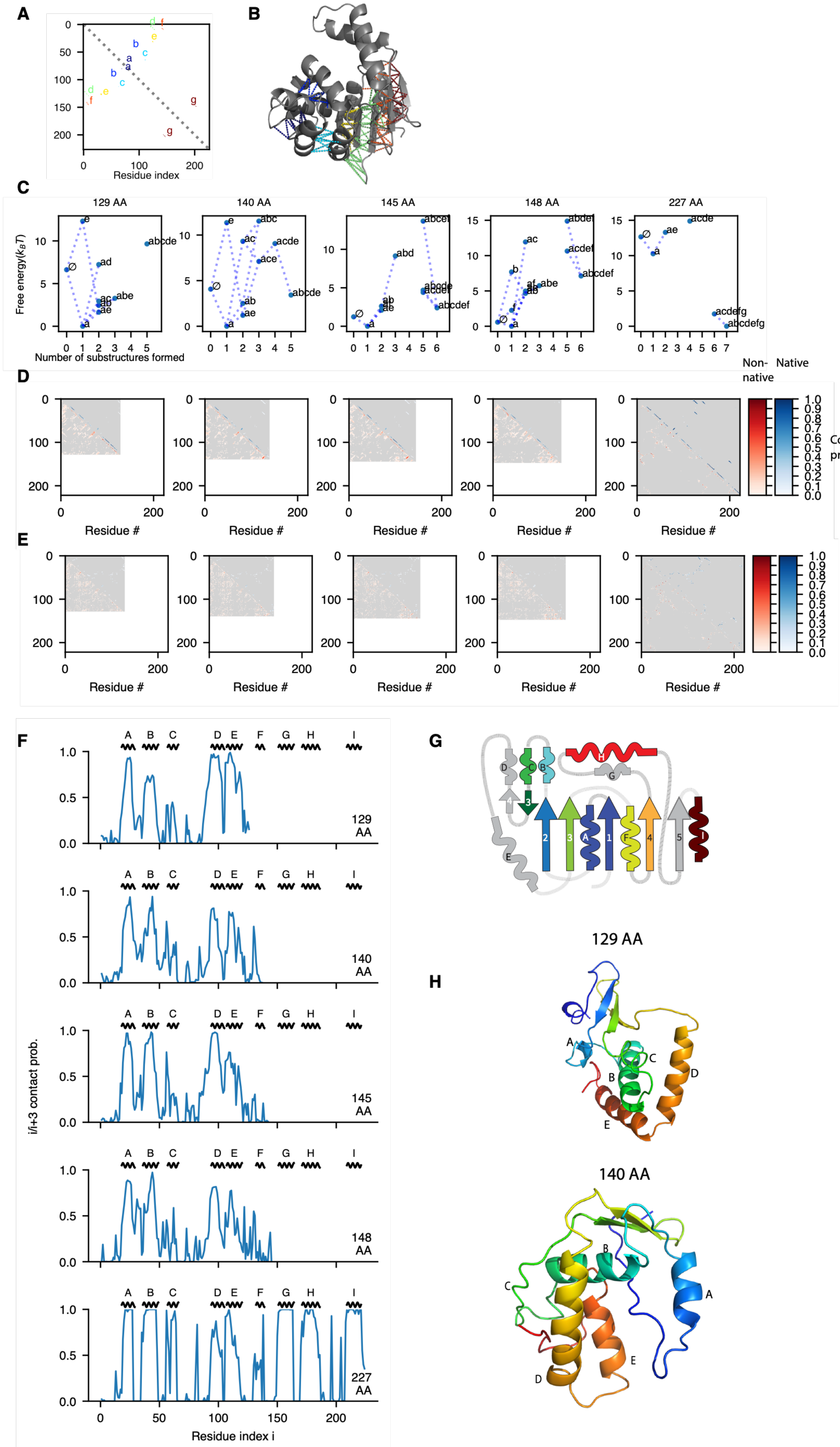

bioRxiv preprint doi: https://doi.org/10.64898/2026.07.03.736445; this version posted August 10, 2026. The copyright holder for this preprint (which was not certified by peer review) is the author/funder, who has granted bioRxiv a license to display the preprint in perpetuity. It is made available under a CC-BY 4.0 International license.

fig. S3 DBFOLD simulations predict that the CMPK NMP subdomain folds co-translationally while core folding is delayed until after translation

Replica-exchange Monte-Carlo simulations with native-contact centric umbrella biasing using the MCPU method from reference *(29)* were reanalyzed as follows: **(A)** CMPK native contact map substructures, defined as in *(58),* shown in color and labeled*.* Each substructure defines a set of contiguous native contacts that is expected to form cooperatively during protein folding. For details on this substructure approach, see *(58)* **(B)** CMPK substructures mapped onto the native structure—each dashed line is a native contact belonging to a given substructure, color-coded as in (A). **(C)** Potentials of mean force (PMFs) showing free energy as a function of topological configuration—defined as a state in which a given subset of substructures is formed or absent--for CMPK constructs in which various numbers of amino acids (AAs) have been synthesized. This analysis was performed using a slightly more lenient *f* parameter (defined as the maximum allowable ratio of the average distance between contacting residues in a given snapshot, to that same distance in the native state, such that a substructure involving those contacts is deemed present in the snapshot) than in reference *(29)* to more accurately capture the presence of dynamic inter-helix contacts. These PMFs are shown at a simulation temperature of T=0.5 (in arbitrary simulation energy units), at which point the folding free energy at full length (227 AA) is about 10 k_BT, comparable to the experimentally-measured value of 6.4 kcal/mol for CMPK in *(69)* **(D)** Average alpha-carbon contact maps, computed using the MBAR method *(70)*, as a function of translation length at a simulation temperature of T=0.5. Two alpha-carbons are deemed in contact in a given snapshot if their distance is within 6 Angstroms. The color shading reflects contact probability (as shown in colorbar) and blues in the upper triangular region reflect native contacts, while nonnative contacts are shown in shades of red in the lower triangular region. For details on this approach, see *(40)*. This analysis reveals that native N-terminal alpha-helices form co-translationally with high probability, but native beta sheets (with the exception of beta hairpin belonging to the NMP-binding subdomain, indicated as substructure *a* in previous panels) do not form until translation is complete. **(E)** Same as (D) but now showing average side-chain contact maps, with a distance cutoff threshold of 5 Angstroms for defining a contact. These contact maps reveal a diversity of interconverting, nonnative tertiary packings at intermediate lengths, while stable, persistent native contacts do not form until full length as reflected by the dark shades of blue at length 227. **(F)** The third diagonal from the upper right triangular region in panel (D) is extracted at each chain length and plotted to reveal the probability of alpha helical contacts between each residue pair i and i+3. The locations for native alpha helices (labeled as in main text Fig. 2) are indicated above the panels. According to this analysis, DBFOLD simulations predict a high probability of NMP subdomain helix formation at intermediate chain lengths while core helices form post-translationally. The exception is core helix A, which is predicted to form co-translationally—this is in disagreement with experimental results and likely reflects an artificial over-stabilization of this helix in the simulation forcefield. **(G)** Topology map for CMPK with secondary structures labeled and color coded as in Fig. 2. **(H)** Sample simulation snapshots from indicated lengths with helices labeled. Note that these snapshots are shown in chainbow coloring that does not match the coloring shown in panel G.

bioRxiv preprint doi: https://doi.org/10.64898/2026.07.03.736445; this version posted August 10, 2026. The copyright holder for this preprint (which was not certified by peer review) is the author/funder, who has granted bioRxiv a license to display the preprint in perpetuity. It is made available under a CC-BY 4.0 International license.

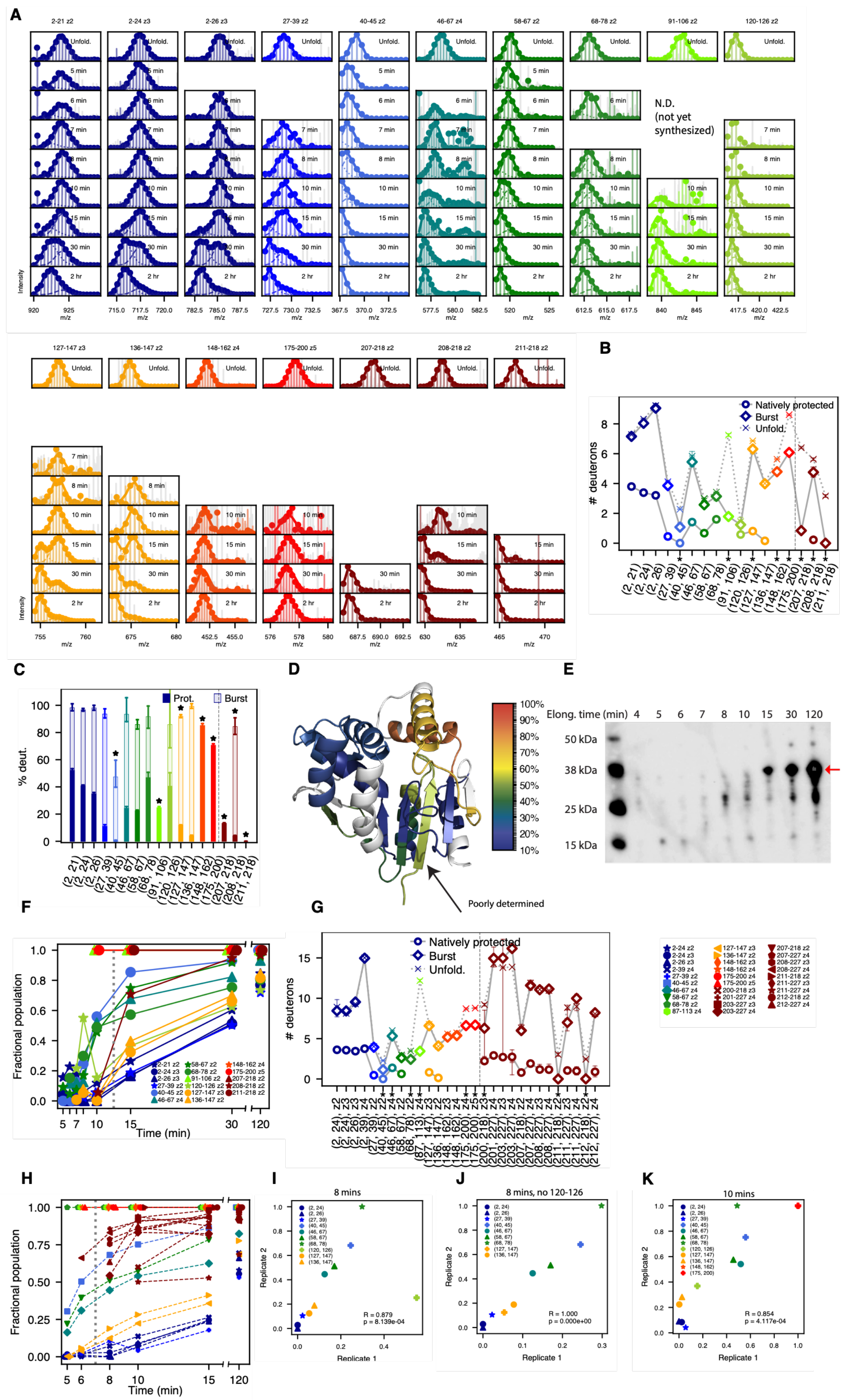

bioRxiv preprint doi: https://doi.org/10.64898/2026.07.03.736445; this version posted August 10, 2026. The copyright holder for this preprint (which was not certified by peer review) is the author/funder, who has granted bioRxiv a license to display the preprint in perpetuity. It is made available under a CC-BY 4.0 International license.

fig. S4 CMPK synchronized translation + pulse-labeling HDX-MS
(A) Mass spectra for all CMPK peptides derived from unfolded control (top row) and all elongation times, as in main text Fig. 2B. Empty panels reflect lack of peptide signal, either because the peptide is not yet synthesized or due to stochastic variabilities in mass-spectrometric detection for low-abundance peptides. **(B)** Number of deuterons associated with natively protected mode (circles), burst-phase mode (the unprotected mode observed early in translation, diamonds) and unfolded state (Xs) for each peptide. Unfolded deuteration levels are corrected for 10 second labeling (see Materials and Methods). Error bars represent 95% confidence intervals from bootstrapping and asterisks denote peptides exhibiting statistically significant burst-phase protection (see Methods). Peptides to the right of the dashed line are only detected after 10 mins (post-translation). **(C)** Same data as (B) but with deuterations expressed as a percentage of maximally deuterated value. **(D)** Percentage deuterations of all peptides are mapped onto the CMPK structure (PDB ID *2cmk*). In general, protection from deuteration correlates with secondary structure. Beta strand 1 appears to show anomalously high deuteration as it is only spanned by large peptides that encompass significant disordered regions, and hence its deuteration cannot be accurately determined. **(E)** Anti-biotin western blot (as in main text Fig. 1C.) showing progress of CMPK synchronized translation reaction as a function of time. This blot reflects the replicate that was used for pulse-labeling experiments shown in main text Fig. 2 and panel (A). Red arrow indicates full-length protein. At around 8 mins, we observe appreciable accumulation of a ~26 kDa translation intermediate, which corresponds to translation of roughly 180 AA from CMPK (with additional ~6 kDa due to the N terminal AviTag + linker). At this stage, the lid subdomain (residues 36-120) has fully emerged from the ribosomal exit tunnel, which typically sequesters the last 30-50 AA. Our observation that lid folding begins around this time (Fig. 2C) strongly suggests that this subdomain folds shortly after its emergence from the ribosome. **(F)** Same as main text Fig. 2C, now showing all peptides with high-quality mass spectra and replicate charge states where present **(G)** Same as (B) for replicate CMPK synchronized translation reaction. **(H)** Same as main text Fig. 2C for a replicate CMPK synchronized translation reaction. As in the first replicate, we observe that lid peptides synchronously begin acquiring protection prior to completion of synthesis, followed by post-translational core folding and rapid NMP subdomain folding. **(I)** To confirm peptides acquire protection in a reproducible sequence across replicates, we compare, for each peptide, the fractional population associated with the natively protected mode at 8 mins in both replicates. Peptides are color coded as in previous panels. A high Spearman correlation coefficient and low p value (shown on the plot) indicate a high degree of reproducibility. **(J)** Same as (I) omitting peptide 120-126 which is poorly fit in the first replicate (see panel A). **(K)** Same as (I) now shown at the 10 mins timepoint.

bioRxiv preprint doi: https://doi.org/10.64898/2026.07.03.736445; this version posted August 10, 2026. The copyright holder for this preprint (which was not certified by peer review) is the author/funder, who has granted bioRxiv a license to display the preprint in perpetuity. It is made available under a CC-BY 4.0 International license.

A

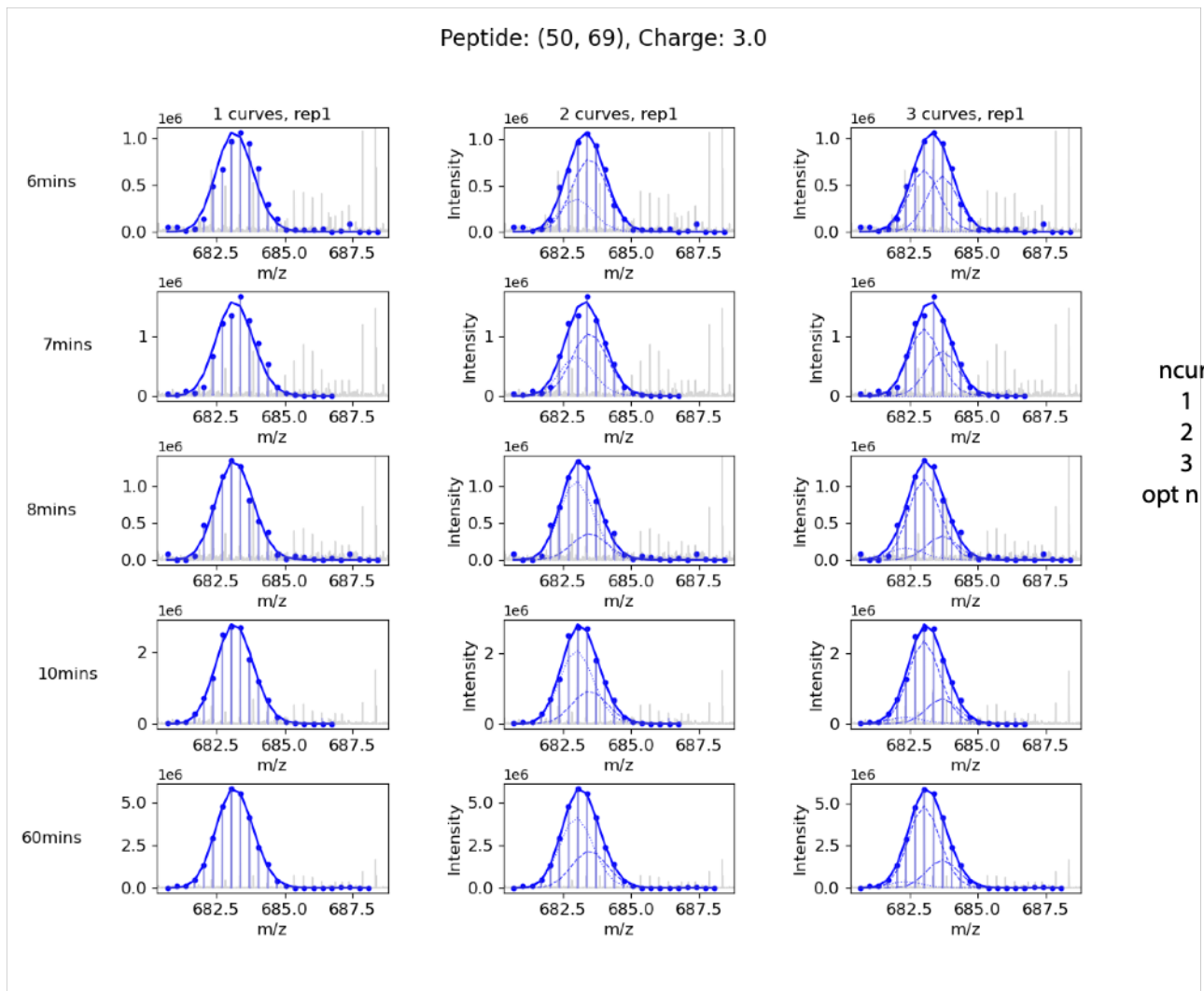

ncurves	RSS	R^2	k	n	BIC
1	0.011483	0.972820	7	95	-825.096922
2	0.004037	0.990444	14	95	-892.528740
3	0.003607	0.991462	21	95	-871.352148

opt n curves = 1

B

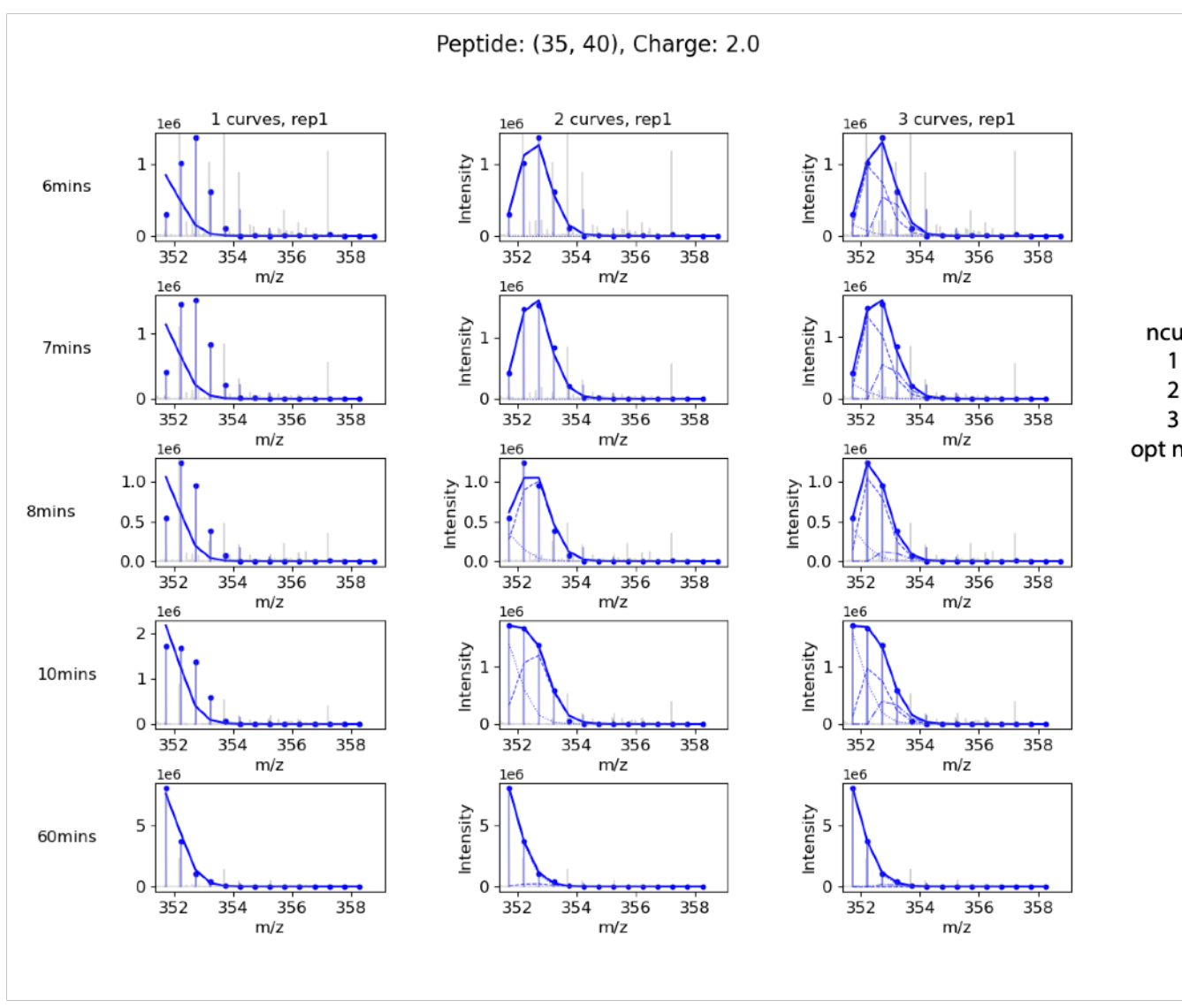

ncurves	RSS	R^2	k	n	BIC
1	0.922705	-0.259103	7	30	-80.640907
2	0.011201	0.984716	14	30	-189.172831
3	0.003223	0.995602	21	30	-202.733914

opt n curves = 2

C

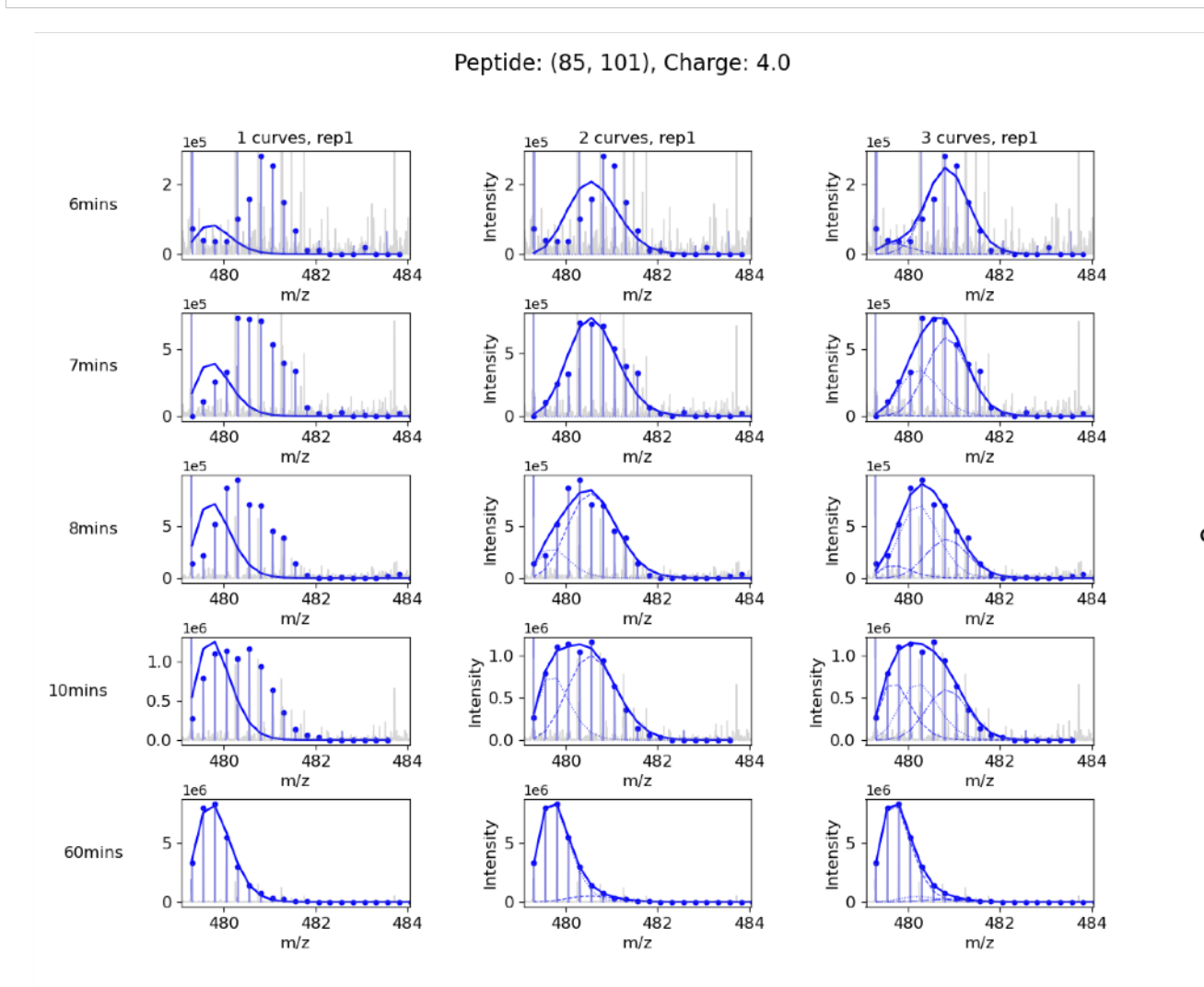

ncurves	RSS	R^2	k	n	BIC
1	0.621762	-0.570887	7	80	-357.903718
2	0.045262	0.885646	14	80	-536.837308
3	0.011983	0.969725	21	80	-612.480767

opt n curves = 3

bioRxiv preprint doi: https://doi.org/10.64898/2026.07.03.736445; this version posted August 10, 2026. The copyright holder for this preprint (which was not certified by peer review) is the author/funder, who has granted bioRxiv a license to display the preprint in perpetuity. It is made available under a CC-BY 4.0 International license.

fig. S5 Determination of optimal number of curves for fitting aTS peptides. **(A)-(C)** For indicated peptides and charge states, we globally fit mass spectra from aTS synchronized translation experiments to either one, two or three curves. Mass spectra for each translation time and number of curves, alongside fits, are shown as in main text Fig. 2B. To the right of each set of plots, we indicate the RSS (residual sum of squares), R^2 (goodness of fit), k (number of free fitting parameters), n (number of fitted datapoints) and BIC (Bayesian information criterion) associated with each number of curves, as well as the optimal number, determined as described in Materials and Methods.

bioRxiv preprint doi: https://doi.org/10.64898/2026.07.03.736445; this version posted August 10, 2026. The copyright holder for this preprint (which was not certified by peer review) is the author/funder, who has granted bioRxiv a license to display the preprint in perpetuity. It is made available under a CC-BY 4.0 International license.

A

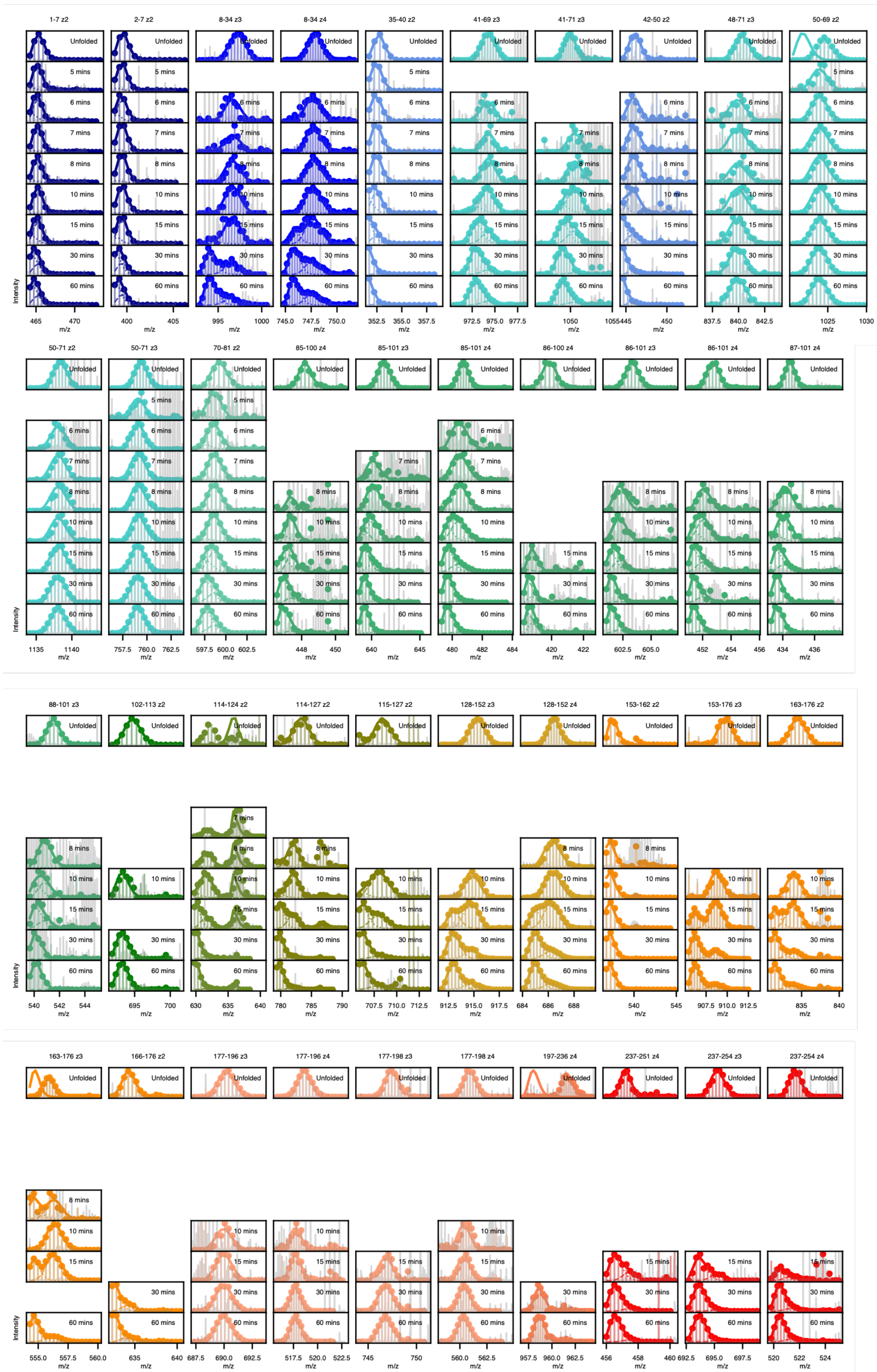

bioRxiv preprint doi: https://doi.org/10.64898/2026.07.03.736445; this version posted August 10, 2026. The copyright holder for this preprint (which was not certified by peer review) is the author/funder, who has granted bioRxiv a license to display the preprint in perpetuity. It is made available under a CC-BY 4.0 International license.

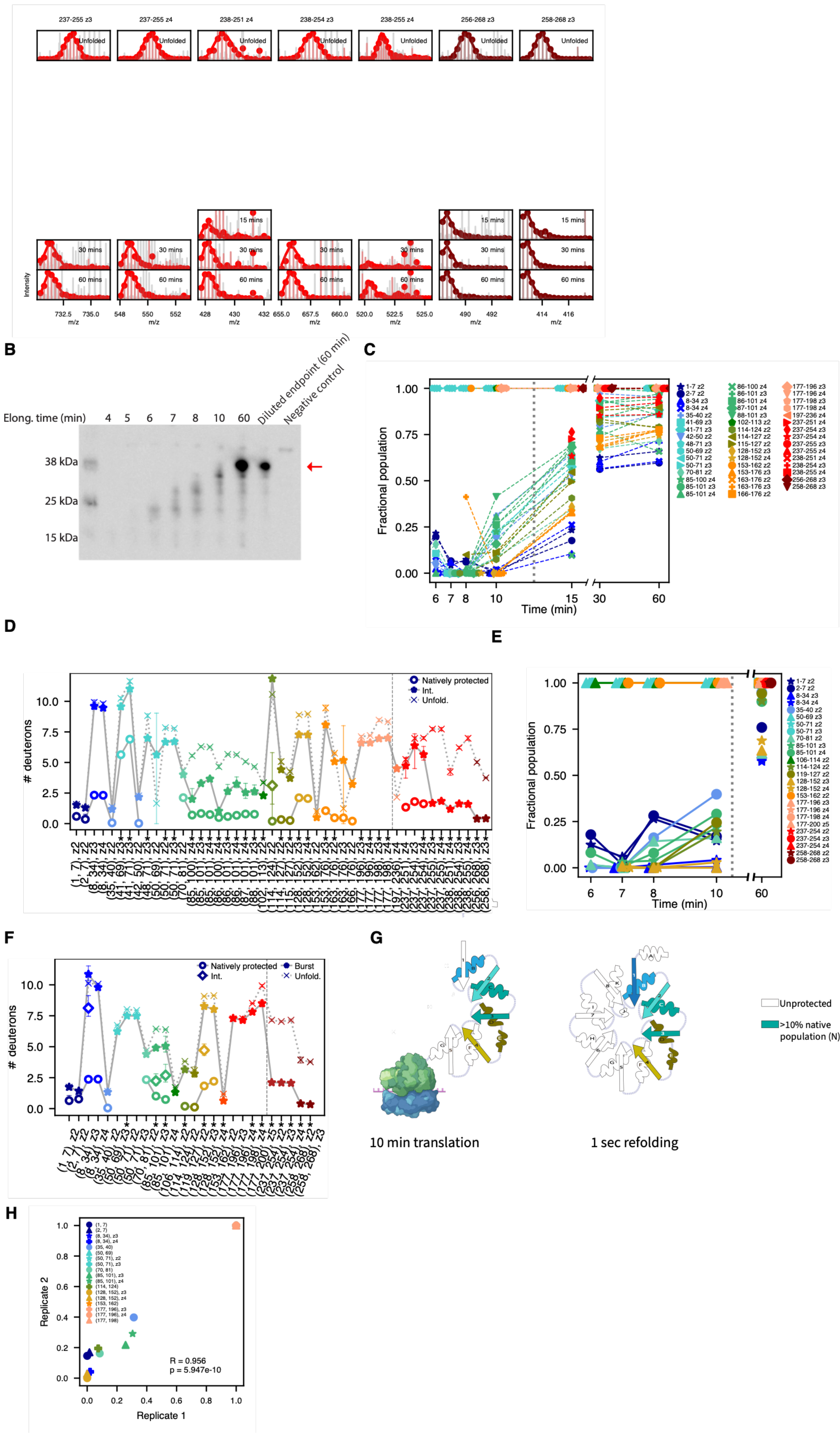

bioRxiv preprint doi: https://doi.org/10.64898/2026.07.03.736445; this version posted August 10, 2026. The copyright holder for this preprint (which was not certified by peer review) is the author/funder, who has granted bioRxiv a license to display the preprint in perpetuity. It is made available under a CC-BY 4.0 International license.

fig. S6 aTS synchronized translation + pulse-labeling HDX-MS
(A) Mass spectra for all aTS peptides derived from unfolded control (top row) and all elongation times, represented as in main text Fig. 2B. Empty panels reflect lack of peptide signal, either because the peptide is not yet synthesized or due to stochastic variabilities in mass-spectrometric detection for low-abundance peptides. **(B)** Anti-biotin western blot (as in main text Fig. 1C.) showing progress of aTS synchronized translation reaction as a function of time. This blot reflects the replicate that was used for pulse-labeling experiments shown in main text Fig. 2 and panel (A). Red arrow indicates full-length protein. (**C)** Same as main text Fig. 2G, now showing all peptides with high-quality mass spectra and replicate charge states where present. **(D)** Number of deuterons associated with natively protected mode (circles), intermediately protected mode where present (diamonds), burst-phase mode (the deprotected mode observed early in translation, asteriks) and unfolded state (Xs) for each peptide. Unfolded deuteration are corrected for 10 second labeling (see Materials and Methods). Error bars represent 95% confidence intervals from bootstrapping and asterisks denote peptides exhibiting statistically significant burst-phase protection (see Methods). Peptides to the right of the dashed line are only detected after 10 mins (post-translation). Various aTS peptides optimally fit to a single mode, For these peptides, only a star and x are shown, representing deuteration of the single global fit mode and the unfolded mode, respectively. **(E)** Same as panel C for a replicate aTS synchronized translation reaction. **(F)** Same as (D) for replicate aTS synchronized translation reaction **(G)** Comparison of folding intermediates populated during aTS translation and refolding from denaturant. Left panel shows secondary structures spanned by peptides showing a natively protected fractional population of at least 10%, provided the deuterium uptake in this native mode is no more than 50% that in the maximally deuterated control, as in main text Fig. 2H, while right panel shows structures spanned by at least one peptide showing a natively protected fractional population of at least 10% at 1 second of refolding from denaturant in the pulse-labeling HDX-MS experiment from Wintrode et. al. 2005 *(50).* For numerical values, see table S4. The co-translational and refolding intermediates largely resemble each other with the exceptions of beta strand 1, which is protected during refolding but not in the co-translational intermediate. **(H)** To confirm peptides acquire protection in a reproducible sequence across replicates, we compare, for each peptide, the fractional population associated with the natively protected mode at 10 mins in both replicates. Peptides are color coded as in previous panels. A high Spearman correlation coefficient and low p value (shown on the plot) indicate a high degree of reproducibility.

bioRxiv preprint doi: https://doi.org/10.64898/2026.07.03.736445; this version posted August 10, 2026. The copyright holder for this preprint (which was not certified by peer review) is the author/funder, who has granted bioRxiv a license to display the preprint in perpetuity. It is made available under a CC-BY 4.0 International license.

Peptide	Charge	Native Fraction (10 min translation)	Native Fraction (1 sec refolding)
**(1, 7)**	2	0	0.04
**(2, 7)**	2	0.02	N.D.
**(8, 11)**	N.S.	N.D.	0.05
**(8, 34)**	3	0	N.D.
**(8, 34)**	4	0.02	N.D.
**(12, 19)**	N.S.	N.D.	0.22
**(20, 34)**	N.S.	N.D.	0.46
**(35, 40)**	2	0.31	0.44
**(40, 46)**	N.S.	N.D.	0.31
**(41, 49)**	N.S.	N.D.	0.44
**(41, 69)**	3	0.21	N.D.
**(41, 71)**	3	0.22	N.D.
**(42, 50)**	2	0.19	N.D.
**(48, 71)**	3	1	N.D.
**(50, 65)**	N.S.	N.D.	N.D.
**(50, 69)**	2	1	N.D.
**(50, 71)**	2	1	N.D.
**(50, 71)**	3	1	N.D.
**(70, 81)**	2	0.08	0.16
**(82, 86)**	N.S.	N.D.	0.79
**(85, 100)**	4	0.22	N.D.
**(85, 101)**	3	0.26	N.D.
**(85, 101)**	4	0.31	N.D.
**(86, 101)**	4	0.26	0.43
**(86, 101)**	3	0.08	0.43
**(87, 101)**	4	0.16	N.D.
**(88, 101)**	3	0.41	N.D.
**(102, 105)**	N.S.	N.D.	N.D.
**(102, 113)**	2	1	N.D.
**(106, 113)**	N.S.	N.D.	N.D.
**(114, 118)**	N.S.	N.D.	0.15
**(114, 124)**	2	0.07	N.D.
**(114, 127)**	2	0.12	N.D.
**(115, 124)**	N.S.	N.D.	0.11
**(115, 127)**	2	0.11	N.D.

bioRxiv preprint doi: https://doi.org/10.64898/2026.07.03.736445; this version posted August 10, 2026. The copyright holder for this preprint (which was not certified by peer review) is the author/funder, who has granted bioRxiv a license to display the preprint in perpetuity. It is made available under a CC-BY 4.0 International license.

**(128, 134)**	N.S.	N.D.	N.D.
**(128, 152)**	3	0	0.06
**(128, 152)**	4	0	0.06
**(153, 162)**	2	1	N.D.
**(153, 176)**	3	0	N.D.
**(163, 172)**	N.S.	N.D.	0.07
**(163, 176)**	3	0	0.07
**(163, 176)**	2	0	0.07
**(177, 196)**	4	1	N.D.
**(177, 196)**	3	1	N.D.
**(177, 198)**	4	1	0.03
**(199, 236)**	N.S.	N.D.	0.1
**(237, 251)**	N.S.	N.D.	0.04
**(258, 268)**	N.S.	N.D.	0.05

Table S4: aTS co-translational folding intermediate resembles refolding intermediate from denaturant. For each peptide and charge state, we indicate the fraction protected in both the 10 minute co-translational timepoint (our experiment) and the 1 second refolding timepoint (Wintrode et. al. 2005) *(50)*. N.D. indicates peptide is not detected in the respective experiment, and N.S. indicates that the charge state was not specified for peptides in Wintrode et. al. 2005.

bioRxiv preprint doi: https://doi.org/10.64898/2026.07.03.736445; this version posted August 10, 2026. The copyright holder for this preprint (which was not certified by peer review) is the author/funder, who has granted bioRxiv a license to display the preprint in perpetuity. It is made available under a CC-BY 4.0 International license.

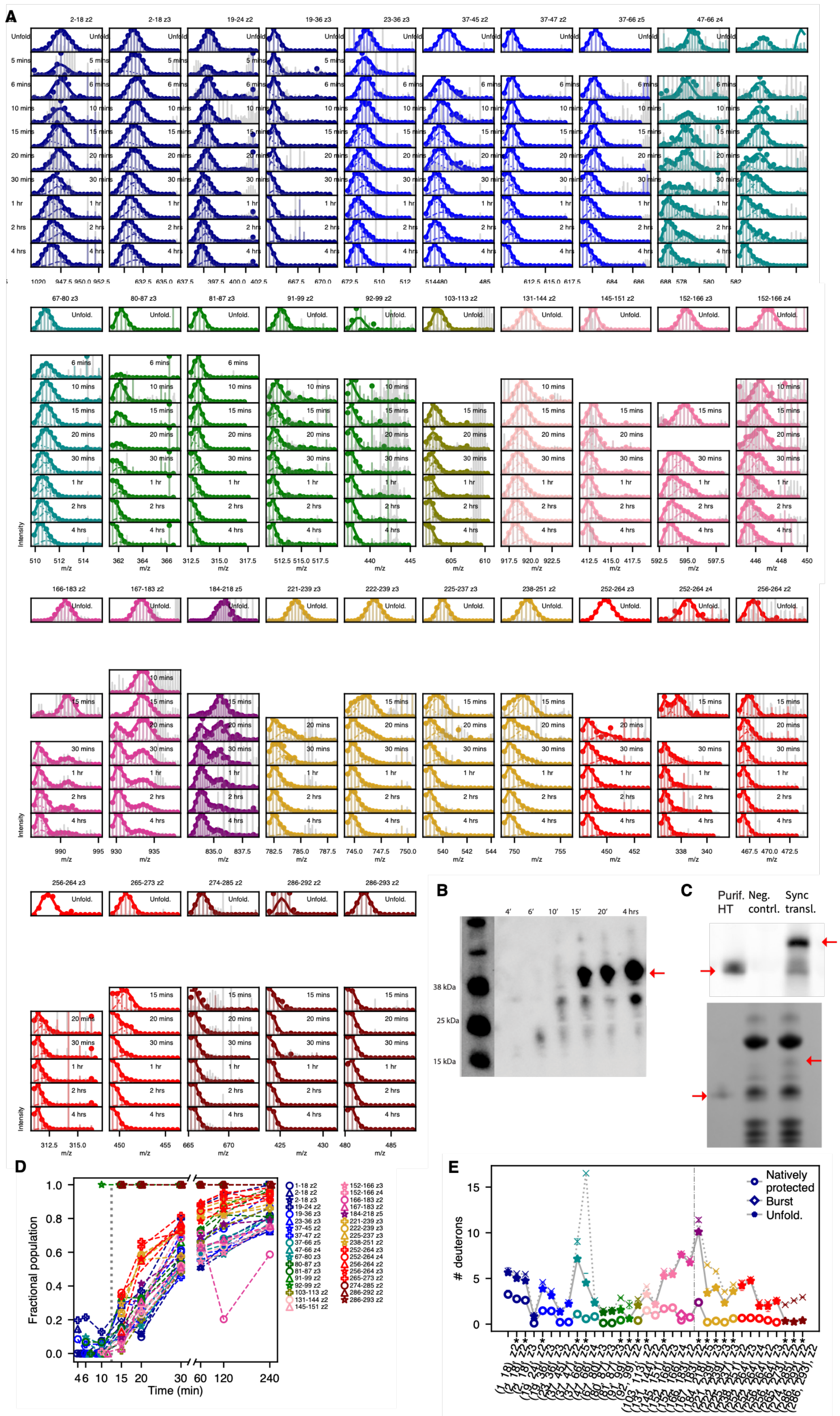

bioRxiv preprint doi: https://doi.org/10.64898/2026.07.03.736445; this version posted August 10, 2026. The copyright holder for this preprint (which was not certified by peer review) is the author/funder, who has granted bioRxiv a license to display the preprint in perpetuity. It is made available under a CC-BY 4.0 International license.

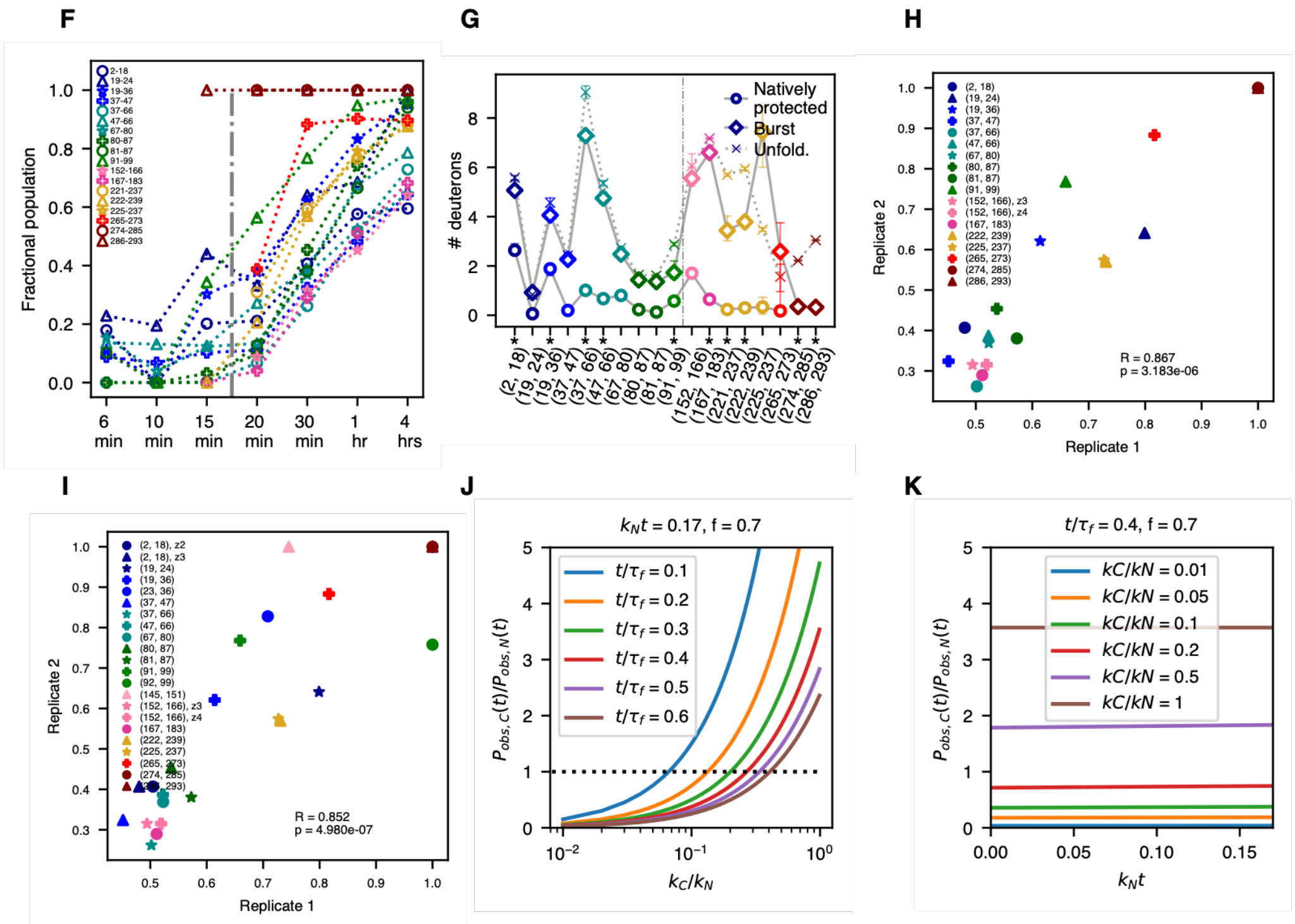

fig. S7: HaloTag synchronized translation + pulse-labeling HDX-MS
(A) Mass spectra for all HaloTag peptides derived from unfolded control (top row) and all elongation times, represented as in main text Fig. 2B. Empty panels reflect lack of peptide signal, either because the peptide is not yet synthesized or due to stochastic variabilities in mass-spectrometric detection for low-abundance peptides. **(B)** Anti-biotin western blot (as in main text Fig. 1C.) showing progress of HaloTag synchronized translation reaction as a function of time. This blot reflects the replicate that was used for pulse-labeling experiments shown in main text Fig. 2 and panel (A). Red arrow indicates full-length protein. **(C)** Top: To verify HaloTag folds into its native structure in our experimental conditions, we perform a synchronized translation reaction in the presence of 10 µM of fluorescent HaloTag ligand TMR and load the final timepoint on an SDS-PAGE gel which we fluorescently image to confirm covalent TMR bidning (middle lane). As a negative control, we perform an equivalent translation reaction without the HaloTag gene (rightmost lane), and we also load purified HaloTag that was similarly incubated with 10 µM TMR as a positive control (leftmost lane). Translated HaloTag runs larger than purified protein due to the added presence of the N-terminal AviTag and linker. Truncated fragments are also observed to bind TMR to some extend as seen previously *(9)*. Bottom: Coomassie-stained gel from above. In both images, red arrows indicate HaloTag bands. **(D)** Same as main text Fig. 3C now showing all peptides with high-quality mass spectra and replicate charge states where present. **(E)** Same as main text Fig. 3D now showing all peptides with high-quality mass spectra and replicate charge states where present. **(F)** Same as main text Fig. 3C for a replicate HaloTag synchronized translation reaction. As in our first replicate, peptides from across the protein do not begin showing native protection until after translation is complete, and we observe protection in three similar stages, namely 1) rapid folding of C-terminal most helices,

bioRxiv preprint doi: https://doi.org/10.64898/2026.07.03.736445; this version posted August 10, 2026. The copyright holder for this preprint (which was not certified by peer review) is the author/funder, who has granted bioRxiv a license to display the preprint in perpetuity. It is made available under a CC-BY 4.0 International license.

2) folding of C-terminal regions of the core, with limited involvement from the N-terminus, and 3) folding of the majority of N-terminus and lid. We note that translation occurred at a slightly slower and yielded slightly reduced peptide coverage than the initial replicate, particularly near the C-terminus. **(G)** Same as main text Fig. 3C for replicate HaloTag synchronized translation reaction. As in the initial replicate, we observe subtle albeit significant burst-phase protection at various N-terminal peptides. We note that for peptide 91-99, the maximally deuterated control for this replicate gave poor spectra, and we thus used the spectra from the initial replicate instead. We validated this substitution by verifying that the maximally deuterated for this peptide when derived from stalled nascent chains showed nearly identical deuteration. **(H)** To confirm that the order of peptide protection is consistent across replicates, we compare, for each peptide, the fractional population associated with the natively protected mode at 30 mins in both replicates. The x and y axis denote the native population fraction in the two respective replicates at the 30 min timepoint. Peptides are color coded as in previous panels. A high Spearman correlation coefficient and low p value (shown on the plot) indicate a high degree of reproducibility. This plot only includes the subset of peptides with high-quality fits in both replicates that are plotted in main text Fig. 3. **(I)** Same as (H) now showing *all* peptides common to both replicates. **(J)** Plots of eq. 12 in supplementary note 3 as a function of k_C/k_N for indicated parameter values. **(K)** Same as J, now as a function of $k_N t$.

bioRxiv preprint doi: https://doi.org/10.64898/2026.07.03.736445; this version posted August 10, 2026. The copyright holder for this preprint (which was not certified by peer review) is the author/funder, who has granted bioRxiv a license to display the preprint in perpetuity. It is made available under a CC-BY 4.0 International license.

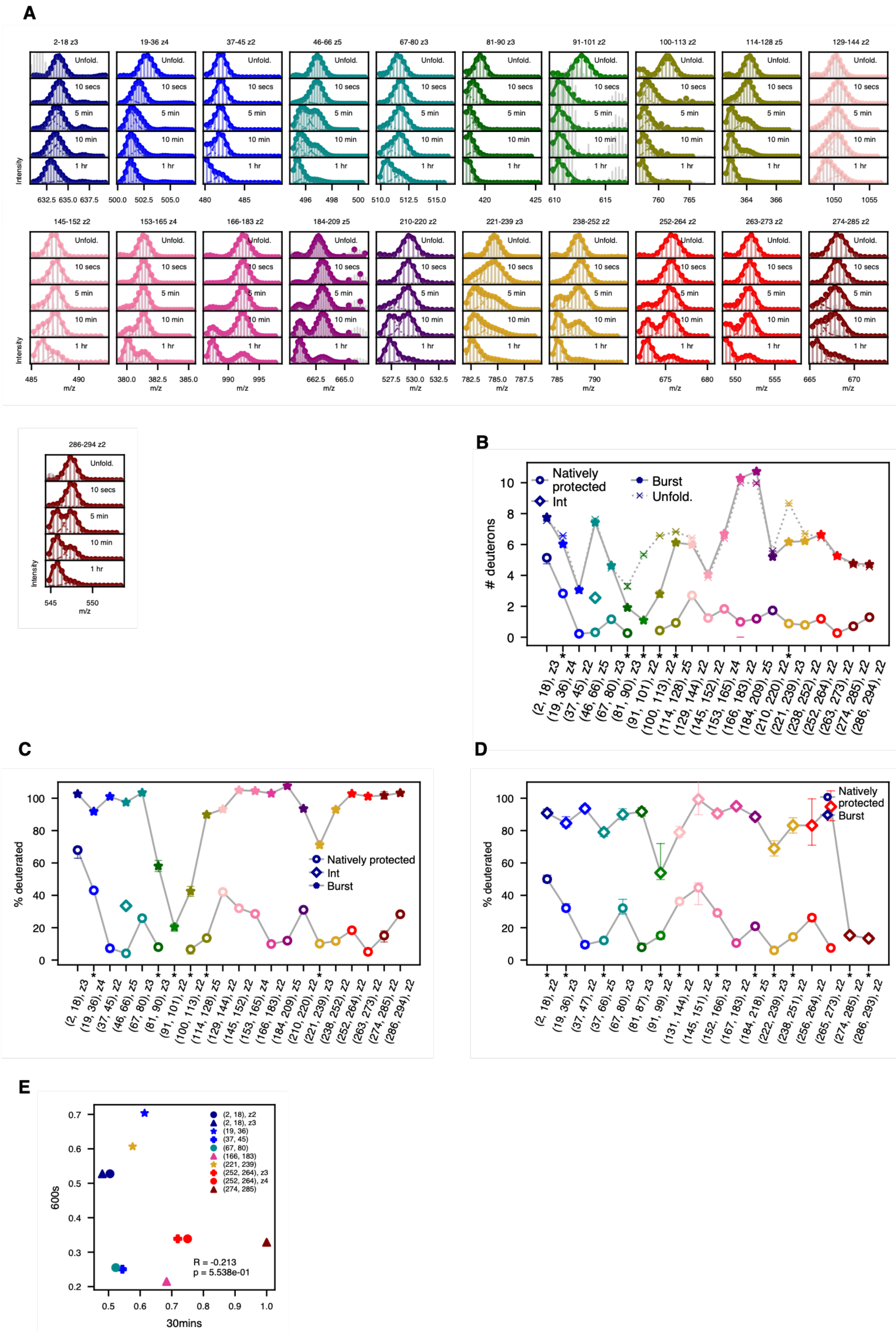

Fig. S8: HaloTag refolding from denaturant + pulse-labeling HDX-MS
(A) Mass spectra for all HaloTag peptides derived from unfolded control (top row) and all timepoints during refolding from denaturant, as in main text Fig. 2B. Data were obtained from reference *(43)* and reanalyzed using the same global fitting approach applied to co-translational

bioRxiv preprint doi: https://doi.org/10.64898/2026.07.03.736445; this version posted August 10, 2026. The copyright holder for this preprint (which was not certified by peer review) is the author/funder, who has granted bioRxiv a license to display the preprint in perpetuity. It is made available under a CC-BY 4.0 International license.

experiments as described in Materials and Methods . **(B)** Number of deuterons associated with natively-protected mode (circles), intermediately-protected mode where present (stars), burst-phase mode (the deprotected mode observed early in translation, diamonds) and unfolded state (Xs) for each peptide. Error bars represent 95% confidence intervals from bootstrapping and asterisks denote peptides exhibiting statistically significant burst-phase protection (see Methods). (**C)** Same as (B) with all # of deuteron values expressed as a percentage relative to the respective values in the unfolded state. (**D)** Same as main text Fig. 3D, which shows deuteration of native and burst-phase modes in active translation experiment, with all # of deuteron values expressed as a percentage relative to the respective values in the unfolded state. We note that the pattern of burst-phase protection differs from that observed in the refolding experiment, with generally higher protection seen across the N terminus. This strongly suggests that the burst-phase intermediate in co-translational folding differs from that observed in refolding. **(E)** Correlation scatterplot between natively protected fractions in 30 minutes timepoint from first replicate of co-translational folding experiment (x-axis) and 10-minute timepoint of refolding experiment (y-axis). At these timepoints, all peptides have begun to show some degree of native population in both experiments. The low Spearman correlation provides further support that these proceed through different intermediates.

bioRxiv preprint doi: https://doi.org/10.64898/2026.07.03.736445; this version posted August 10, 2026. The copyright holder for this preprint (which was not certified by peer review) is the author/funder, who has granted bioRxiv a license to display the preprint in perpetuity. It is made available under a CC-BY 4.0 International license.

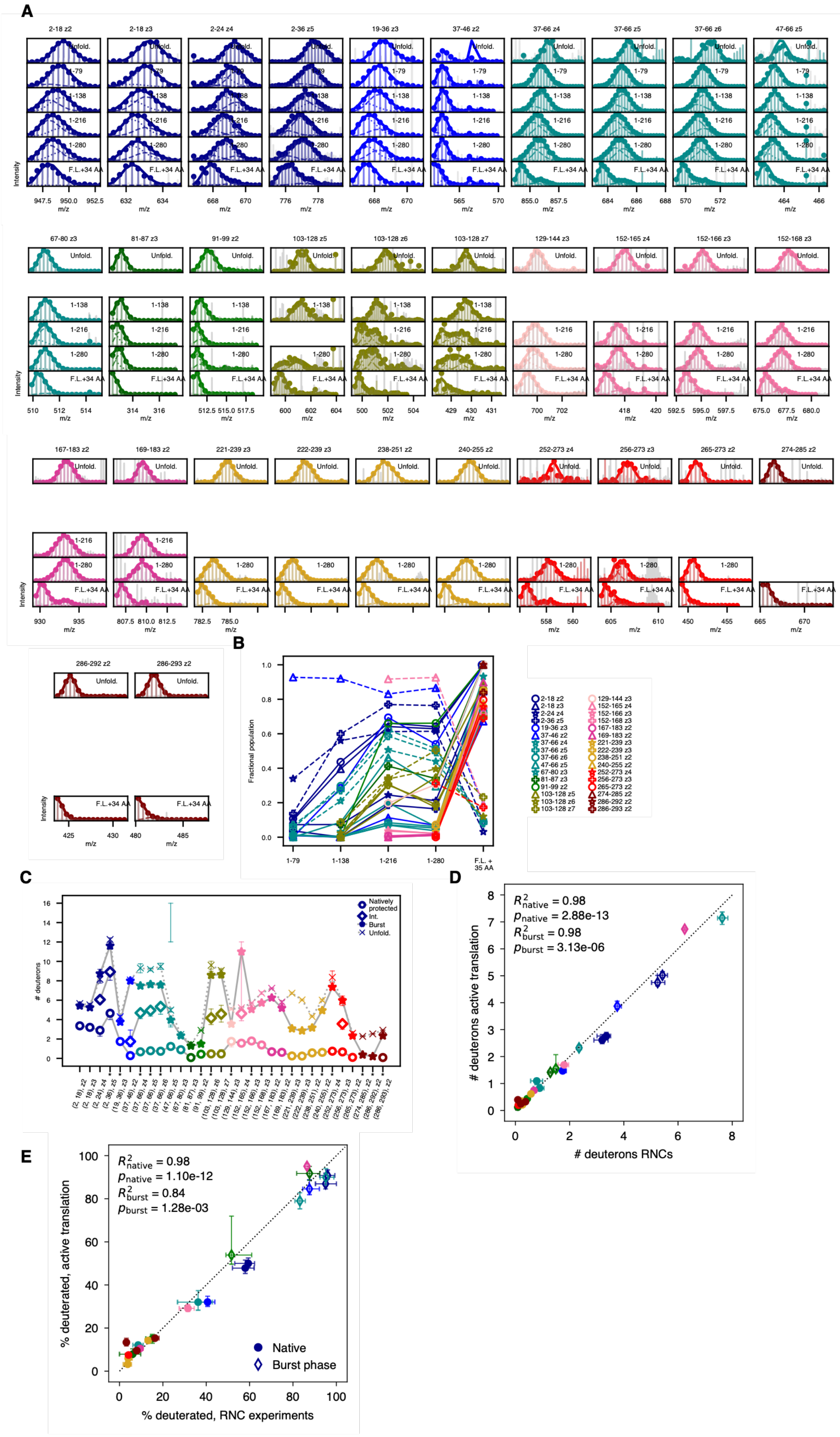

bioRxiv preprint doi: https://doi.org/10.64898/2026.07.03.736445; this version posted August 10, 2026. The copyright holder for this preprint (which was not certified by peer review) is the author/funder, who has granted bioRxiv a license to display the preprint in perpetuity. It is made available under a CC-BY 4.0 International license.

Fig. S9: HaloTag stalled ribosomal nascent chains (RNCs) + pulse-labeling HDX-MS
(A) Mass spectra for all HaloTag RNC peptides derived from unfolded control (top row) and all translation lengths, as in main text Fig. 4B. Empty panels reflect lack of peptide signal, either because the peptide is not yet synthesized at that length or due to stochastic variabilities in mass-spectrometric detection for low-abundance peptides. **(B)** Same as main text Fig. 4E, now showing all peptides with high-quality mass spectra and replicate charge states where present. **(C)** Number of deuterons associated with natively protected mode (circles), intermediately protected mode where present (stars), burst-phase mode (the deprotected mode observed early in translation, diamonds) and unfolded state (Xs) for each peptide. Unfolded deuteration are corrected for 10 second labeling (see Materials and Methods). Error bars represent 95% confidence intervals from bootstrapping and asterisks denote peptides exhibiting statistically significant protection, relative to the maximally deuterated control corrected for 10 sec labeling, in the deprotected mode (see Materials and Methods). **(D)** Correlation between deuterium uptake associated with native (circles) and burst phase (diamonds) modes for each peptide in RNC and active translation experiments with indicated Pearson correlations. Error bars represent 95% confidence intervals from bootstrapping. **(E)** Same as (D) but with all values normalized by respective maximally deuterated control values. This normalization corrects for the confounding effect of peptide length.
