# Supplementary material for: A detailed molecular picture of protein folding during active translation
Source: bioRxiv. 2026 Jul 4:2026.07.03.736445. Preprint. [Version 1] doi: 10.64898/2026.07.03.736445 (PMC13345067; doi:10.64898/2026.07.03.736445)
Supplement: Supplement 1 [file media-1.pdf]

Supplementary Materials for

A detailed molecular picture of protein folding during active translation

Amir Bitran, Carlos Bustamante, Susan Marqusee

Correspondence to: marqusee@berkeley.edu

This PDF file includes:

Materials and Methods
Supplementary Text
Figs. S1 to S9
Tables S1 to S4
References

Materials and Methods

Cloning and PCR

Genes encoding proteins of interest used for synchronized translation reactions were cloned via Gibson assembly into a plasmid encoding an ampicillin resistance marker. The gene was inserted downstream of an AviTag as well as a linker region containing a threonine, a stretch of serines and glycines (41 residues for HaloTag, 54 for aTS and CMPK), and three valines. The *cmk* and *TrpA* genes encoding the proteins CMPK and aTS were amplified directly from the *E. coli* K-12 chromosome via PCR, using primers directed against the 5' and 3' ends of the gene prior to Gibson assembly. The identity of the inserts on the respective plasmids were verified by Sanger sequencing.

As a template transcription + synchronized translation reactions, the genes of interest were reamplified from the plasmids harboring them using a forward primer directed upstream of the T7 promoter, and a reverse primer downstream of the stop codon. The resulting PCR product was DpnI digested to remove template plasmid, followed by PCR purification on a silica gel column and elution using molecular biology grade water (Corning 46-000). Purity and yield were assessed via Nanodrop. Any PCR products showing an A260/A230 absorbance ratio less than 1.8 were further purified using a 0.5 ml Zeba spin desalt column (Thermo Scientific 89883) to remove residual salts.

For stalled RNC experiments, the HaloTag gene alongside an N-terminal AviTag and linker (as described above) were cloned onto a plasmid containing a C-terminal glycine serine linker followed by a strong SecM stalling sequence (FSTPVWIWWWPRIRGPP) using around-the horn (ATH) PCR. In total, this C-terminal region (linker + SecM) contains 34 AA. This plasmid was then used as a template for subsequent ATH PCRs to generate plasmids consisting of various HaloTag C-terminal truncations (HaloTag 1-79, 1-138, 1-216 and 1-280) immediately followed by a strong SecM stalling sequence. Prior to *in vitro* translation, the regions spanning the T7 promoter through the strong SecM stalling sequence were amplified from each plasmid through PCR, and the resulting PCR fragments were purified as described in the previous paragraph.

Synchronized translation reactions

Synchronized translation reactions are performed in two steps: 1.) mRNA transcription and initiation, followed by 2.) single-turnover elongation. The transcription and initiation steps are expected to be slow, and thus it is advantageous to complete them first to maximize subsequent synchrony.

For the transcription + initiation step, reactions are prepared with methionine as the sole amino acid provided, to allow mRNA to be transcribed and initiation complexes to form without elongating. This is achieved by preparing two to four PURE express reactions, depending on the number of timepoints to be taken for HDX-MS. Each HDX-MS timepoint requires 6 µL of material, and an additional 2 µL for western blot samples. Each reaction equivalent contains of 5 µL of Solution A -aa,tRNA (New England Biolabs B6841AVIAL from E6840S), 7.5 µL Solution B -RF123 (NEB P6854AVIAL), 1 µL Murine RNAse inhibitor (NEB M0314L), 1.25 µL of 20 µM BirA , 2.5 µL of *E. coli* tRNA mix (NEB N6842AVIAL), 0.5 µL of 30 mM methionine, 0.5 µl of 1 mM biotin (20 µM final), and 260 ng of purified PCR product encoding gene of interest downstream of an AviTag + glycine serine linker (see previous section) or an

equivalent volume of molecular biology grade water (for negative control reactions), and molecular biology grade water to a total volume of 30 µL. A formyl donor for formation of fmet tRNAs is present in solution A, and the addition of tRNAs at this step provides a source of methionine tRNA; we confirm that no appreciable amino acids are present in the tRNA solution by the observation that no full length protein is detected at early elongation timepoints via western blot, nor are C-terminal peptides detected at these times via mass spectrometry. In certain control experiments, we add 1 µL of FluoroTect GreenLys (Promega L5001), a Lysine tRNA charged with a BODIPY-FL labeled lysine to facilitate in-gel visualization of translated product without requiring western blotting; however, FluoroTect is excluded from reactions that will be analyzed by HDX-MS. This reaction is incubated for 30 mins at 37C to allow transcription to occur and initiation complexes to form. Where indicated, we added a HaloTag TMR Ligand (Promega G825A) to 10 µM final concentration at this stage.

During the initiation period, working elongation mix is prepared by mixing 2.5 µL of complete amino acid mix (NEB N6843AVIAL), 0.5 µL of release factor 1 (NEB P6851AVIAL), release factor 2 (P6852AVIAL), and release factor 3 (P6853AVIAL) each, at least 2.5 µL of pre-charged tRNA for a fincal concentration of >20 µM pre-charged tRNA (>1 µM each) and 0.95 µL of 2.37 mM aurintricarboxylic acid (ATCA, MilliporeSigma 18-940-0100MG) for a final working concentration of 75 µM per reaction equivalent. ATCA is used to prevent reinitiation of ribosomes and ensure single-turnover elongation; we confirm that translation is inhibited when ATCA is added at to the initiation step, but not at the elongation step (SI). ATCA solution is prepared fresh from powder the day of use and stored on ice, protected from light. Working elongation mix is prepared under RNAse free conditions, and pre-warmed at 37C for 10 mins prior to onset of elongation. After 30 mins of initiation, 7.45 µL of elongation mix is added per reaction equivalent of initiation mix to begin elongation. At desired elongation timepoints, a 6 µL of synchronized elongation reaction is drawn in order to perform pulse HDX and nascent chain enrichment (see later section). In addition, at these same times, 1.8 µL of reaction is drawn and mixed with 3.6 µL of 2 mm puromycin (1.33 mM final) to generate samples for western blot analysis. These samples are kept at room temperature for at least 30 seconds, then placed on ice. These were then treated with RNAse A (Thermo Scientific EN0531, 0.5 mg/ml final) for 5 min at 37C, followed by addition of 5.4 µL of 2X Laemmli sample buffer followed by SDS-PAGE and/or western blot analysis (see later section). After collecting the final timepoint, 6 µL was bound to streptavidin beads pre-equilibrated in H2O for preparation of an undeuterated control, and 2 x 6 µL aliquots were used to prepare maximally deuterated samples (see later section).

Although the main text figures show one biological replicate for each active translation reaction, we performed at least two replicates total for each protein, and additional replicates are shown in the supplementary figures. We verified that our main results, particularly order of folding of distinct structural elements before and after end of translation, reproduce across biological replicates, although the precise times at which events occur vary slightly between replicates due to ribosomal batch variations leading to slightly variability in translation rate.

<u>Preparation of pre-charged tRNA</u>

6.25 mg of total E Coli tRNA (Roche MRE600) was weighed out and resuspended in 280 µL of molecular-biology grade water, and mixed with 50 µL of complete amino acid mixture containing roughly 2.2 mM of each AA at near-neutral pH, 20 µL of 100 mM ATP, 50 µL of 10x

charging buffer (final 1x composition of 50 mM k-HEPES pH 7.5, 50 mM KCl, and 5 mM DTT) and 100 µL of DEAE-purified S-100 extract*(1)*. This reaction was incubated for 1 hour at 37C, followed by addition of 80 µL of NaOAC or KOAC pH 5.3 (final concentration of 300 mM) to acidify the reaction and maintain ester bond stability. Protein components were then removed by phenol chloroform precipitation using cold saturated phenol (pH 5.3). tRNA was then precipitated overnight at -80C with 100% ethanol followed by centrifugation for 2 hours at 14,000xg at 4C. The supernatant was removed and the pellet was air-dried for 5-10 mins, followed by resuspension in pH 5.3 and desalting through an illustra MicroSpin G-25 spin column (Cytiva 27-5325-01) pre-equilibrated in 3 mM KoAC. After this stage, absorbance at 260 nm was measured using a NanoDrop to determine tRNA concentration. Pre-charged tRNA was aliquoted, flash frozen in liquid nitrogen and stored at -80C.

<u>Preparation of stalled ribosomal nascent chain samples</u>

A translation master mix was prepared under RNAse-free conditions, consisting of 15 µL Solution A -aa,tRNA (New England Biolabs B6841AVIAL from E6840S), 22.5 µL Solution B -RF123 (NEB P6854AVIAL), 3 µL Murine RNAse inhibitor (NEB M0314L), 3.75 µL of 20 µM BirA (0.7 µM final) , 7.5 µL of *E. coli* tRNA mix (NEB N6842AVIAL), 7.5 µL of complete amino acid mix (NEB N6843AVIAL), and 1.5 µl of 1 mM biotin (14 µM final). This master mix was tehn split into 6 equal aliquots corresponding to the five HaloTag RNC constructs to be generated (with two duplicate reactions for the L34 construct to ensure sufficient volume for undeuterated and maximally deutereated controls), and to each, 130 ng of PCR product encoding the respective gene construct (including N-terminal AviTag + linker and C-terminal strong SecM stalling sequence) was added, as well as molecular biology grade water to a total volume of 15 µL. The reactions were then incubated for 4 hours at 37C. Subsequently, 14 µL of reaction was layered atop a 94 µL sucrose cushion consisting of 1M sucrose in HKMT buffer (25 mM HEPES, 15 mM MgOAC2, 150 mM KCl, 0.1 mM TCEP, pH 7.5) in polycarbonate centrifuge tubes (Beckman Coulter 343775). These samples were then ultracentrifuged in a Beckamn Coulter Optima MAX-XP ultracentrifuge for 80 mins at 200,000xg and 4C using a pre-chilled Beckman Coulter TLA-100 fixed-angle rotor. Following removal of the supernatant, the ribosomal pellets were resuspended in 20 µL HKMT, and the two resuspended pellets corresponding to the L34 construct were pooled together.

All resuspended pellets were now re-equilibrated to 37C for 15 mins, followed by pulse-labeling HDX and streptavidin bead binding as described in the subsequent section. Two technical replicates of the pulse-labeling reaction were performed for each sample. The L34 construct was also used to produce undeuterated and maximally deuterated control samples. Following pulse-HDX, the integrity of the NCs was assessed by mixing 2 µL of resuspended pellet with 10 µL of low-pH sample buffer for a final composition of 7.5% glycerol, 62.5 mM Bis-Tris pH 5.7, 0.01% bromphanol blue (wt/vol), 0.2% DTT (wt/vol) and 2% SDS (wt/vol) *(2)*. These samples were then analyzed by SDS PAGE and western blot (see section Anti-biotin western blotting) to ensure homogeneity of each NC construct and migration ~20-25 kDa larger than expected due to peptidyl-tRNA attachment. To generate "released" samples, a separate aliquot of each construct was treated with RNAse A (final concentration of 0.2 mg/ml, Thermo Scientific EN0531) and incubated for 5 mins at 37C prior to addition of low-pH loading buffer and SDS-PAGE analysis.

<u>Pulse HDX-MS, maximally deuterated sample prep, and nascent chain enrichment</u>

Pierce streptavidin magnetic beads were equilibrated to room temperature and resuspended, then 60 µL of bead slurry was drawn per translation timepoint. Beads were placed against a magnetic stand to remove storage solution and subsequently were washed with 1 ml of nascent chain labeling buffer (10 mM MES, 30 mM KOAc, 12 mM MgOAC2, pH 6.0) in H2O. Beads used in deuterated samples were additionally washed in 0.5 ml of nascent chain labeling buffer in 99.8% D2O (TCI W002 or Thermo 426931000). The beads were then resuspended in 54 of nascent chain labeling buffer in D2O (for deuterated samples) or H2O (for undeuterated controls) per timepoint, and 54.3 µL aliquots corresponding to the respective timepoints of interest were transferred into 0.5 ml Protein LoBind Tubes (Eppendorf 022431064) . These aliquots were pre-warmed for 10 mins at 37C prior to pulse HDX-MS. A pH value of 6.0 (as read by pH meter) was chosen for pulse HDX to ensure that a 10 second labeling pulse would predominantly label solvent-exposed amides, while amides that require structural fluctuations to become accessible would remain unlabeled, as is typical in pulse-labeling HDX. We calculated that this pH would only label EX2 structural fluctuations whose associated energy is less than about 2 kcal per mol at 37C *(3)*.

At indicated elongation timepoints, nascent chains were pulse labeled and bound to streptavidin beads. This was achieved by drawing 6 µL of elongation reaction (see section "Synchronized translation reactions" and thoroughly mixing with the respective bead aliquot in deuterated or undeuterated (for undeuterated control) buffer, prepared as per the previous paragraph. The sample was incubated for 10 seconds at 37C to allow hydrogen-deuterium exchange (for deuterated samples) and nascent chain binding to streptavidin beads, followed by addition of 60 µL of 2x ice-cold quench buffer (3.5M GdnHCl, 1.5M glycine, and 0.5M TCEP, pH 2.4) and rapid mixing. Samples were then flash frozen in liquid nitrogen and stored at -80C until LC-MS injection (see next section).

Maximally deuterated control samples were prepared by mixing 6 µL nascent chains with a 54.3 µL streptavidin bead aliquot pre-equilibrated in D2O, as per the previous paragraph, followed by 15 minute incubation at room temperature on a rotary mixture to allow complete binding of nascent chains to streptavidin beads. The samples were then placed against a magnetic stand and the supernatant was removed. Beads were then resuspended in 60 µL of 8M urea + NC labeling buffer in 90% D2O—this buffer was prepared by resuspending 9M urea in NC labeling buffer in H2O followed by three rounds of lyophilization and resuspension in 90% D2O, and the final urea concentration was adjusted to 8M (as measured by refractometry) using NC labeling buffer in 90% D2O. Beads were incubated overnight at 37C in this deuterated urea buffer, followed by addition of 60 µL of ice cold 2x quench buffer. Samples were then flash frozen in liquid nitrogen and stored at -80C until LC-MS injection. For the first CMPK biological replicate, maximally deuterated samples were incubated at 37C for 3 hours rather than overnight; we confirm this is sufficient to produce maximal deuteration. Maximally deuterated controls were prepared in duplicates for each sample.

Prior to LC-MS injection, samples were thawed by rapid flicking, then passed through a Corning ® Costar ® Spin-X® 0.22 µm centrifugal filter (Costar 8161) by centrifuging for 30 secs at 12,000 x g to separate the quenched labeling solution from the beads, which are retained in the supernatant of the filter. Beads were then resuspended in 1x quench buffer (2x quench buffer diluted in NC labeling buffer in H2O) containing 0.2 mg/ml of porcine pepsin (Sigma P6887) to initiate digestion. A total digestion time of 4 mins was found to yield optimal peptide coverage for HaloTag samples, and 5.5 mins for CMPK and aTS samples. Immediately after resuspension in pepsin-containing 1x quench, beads were vortexed for 5 seconds then put on ice.

Two minutes prior to the end of digestion, the bead-containing centrifugal filter was again centrifuged for 30 secs at 12,000 x g and the flow-through containing peptides was now collected in a clean 1.5 ml lowbind tube, and placed back on ice until the end of the digestion period. At this point, the liquid sample was injected into the LC-MS system.

LC-MS runs

Peptide-containing samples were injected into a cooled valve system (Tajan LEAP) in line with a ThermoUltimate 3000 LC. Peptides were desalted on a C8 pre-column (Waters 186003978) for 4 mins, then subsequently bound to a Waters Acquity C18 analytical column (Waters 86002344), both maintained at 2°C. The bound peptides were gradient-eluted (5–40% acetonitrile w/v and 0.1% w/v formic acid) across the analytical column at a flow rate of 40 µl/min over 14 min followed by a gradient from 40 to 90% buffer B over 30 s. The analytical and trap columns were then sawtooth washed and equilibrated at 5% buffer B. Eluted peptides were analyzed directly using a high-resolution Orbitrap mass spectrometer (Q Exactive, Thermo Fisher) operating in positive mode [resolution, 140,000; automatic gain control target, 3×10^6; maximum injection time, 200 ms; scan range, 300 to 1500 mass/charge ratio (*m*/*z*)]. For each construct, we performed a tandem mass spectrometry experiment on an undeuterated sample (full mass spectrometry resolution 70,000; automatic gain control target, 3×10^6; maximum injection time, 50 ms; scan range, 300 to 1500 mass/charge ratio; dd-MS2 settings as follows: resolution, 17,500; automatic gain control target, 2×10^5; maximum injection time, 100 ms; loop count, 10; isolation window 2.0 *m*/*z*; normalized collision energy, 28; charge state, 1 and ≥8 excluded; dynamic exclusion of 15 s). Between injections, the sample loop and trap column were manually washed at 200ul/min with 50% acetonitrile + 0.1% formic acid for four minutes, followed by one wash of the protease column with 100 µl of 1.6 M GdmCl and 0.1% formic acid and three sawtooth wash of the trap and analytical columns (5-90% acetonitrile gradient each).

SDS-PAGE and Anti-biotin Western blotting

Samples in 1x Laemmli sample buffer (for active translation reactions), or low-pH sample buffer (for stalled RNCs or other samples requiring preservation of peptidyl tRNA integrity) were loaded onto a NuPAGE 4-12% 1.5 mm 15-well gel, alongside 2 µL of WesternSure Pre-stained Chemiluminescent Protein Ladder (LI-COR, 926-98000) for western blto samples, or 0.5 µL of PageRuler plus prestained protein ladder (Thermo Scientific 26619) for samples to be analyzed by fluorescence. The gel was run at 125-130 volts at room temperature, or at 4C (for samples requiring preservation of peptidyl tRNA integrity) for at least one hour in MES running buffer. If gel samples are fluorescently labeled with fluorotect, then the gel was imaged using a Typhoon FLA9500 (GE Healthcare) with a 488-nm laser and a 510LP filter. If labeled with TMR, then a 532 nm excitation laser and 580 nm BP emission filter were used.

Prior to western blot transfer, gels were then rinsed in deionized water and equilibrated for 10 mins in 1x Towbin transfer buffer containing 20% methanol, alongside blotting paper and a 0.2 µm polyvinylidene difluoride membrane (Bio-Rad, 1620174) that had been pre-activated in 100% methanol. Transfer was then performed at 25 V and 1.0 A for 42 minutes using a

TransBlot Turbo apparatus. The membrane was then rinsed in deionized water and incubated with 10 ml EveryBlot Blocking Buffer (Bio-Rad, 12010020) on an orbital shaker for 5 mins at room temperature, or overnight at 4C. The blocking buffer was decanted subsequently incubated in streptavidin solution consisting of 0.5 µL of 2 mg/ml Streptavidin horseradish peroxidase conjugate (Invitrogen™ S911A)in 10 ml Everyblot blocking buffer for 1 hour at room temperature with orbital shaking. The membrane was then washed five times for five minutes with tris-buffered saline solution with tween-20. Prior to chemiluminescent imaging, the membrane was rinsed in deionized water and blotted dry. Then, the blot was imaged via addition of either 5 ml of each solution of Clarity Western ECL substrate (Biorad 1705061), 6 ml of each solution of SuperSignal West Pico PLUS Chemiluminescent Substrate (Bio-Rad, 34580), or 2 ml of each solution of SuperSignal West Femto substrate (Thermo 34094). The membrane was imaged on a Chemi-Doc. Where indicated, an Alexa Fluor™ 488 Streptavidin conjugate (Invitrogen A32361) was used inlieu of Streptavidin HRP, and imaging was carried out using a FLA9500 (GE Healthcare) with a 488-nm laser.

<u>TEV elution experiment</u>

To confirm that streptavidin bead binding does not disrupt nascent chain (NC) integrity, we designed an experiment in which the AviTag is separated from the NC by a linker ending with a TEV cleavage loop, and following bead binding, NCs were eluted via addition of TEV. The TEV cleavege loop (ENLYFQ) was inserted at the end of the linker separating the AviTag from the nascent protein of interest (in this case aTS), and a PCR fragment for *in vitro* transcription + translation was produced as described in subsection Cloning and PCR. This PCR fragment was designed to contain a 35 AA C-terminal linker downstream of aTS, and no stop codon, thus promoting ribosomal stalling at the end of this linker. A translation master mix was prepared consisting of 5 µL solution A -AA, tRNA, 2.5 µL of 10x AA mix, 2.5 µL tRNA, 7.5 µL of solution B-RF123, 1 µL murine RNAse inhibitor, 1 µL of FluoroTect GreenLys, 1.25 µL of 20 µM BirA, and 270 ng PCR fragment. This master mix was then split into three samples (A, B and C), two of which (A and B) were supplemented with biotin to 20 µM, while C was supplemented with an equivalent volume of water. All three were then incubated at 37C for 1 hour to allow for translation to occur. After one hour, sample B was treated with puromycin to a final concentration of 68 µM. At this stage, a 2 µL "input" aliquot was drawn from all three samples and mixed with 8 µL HKMT buffer and low-pH loading dye (to a 1x working concentration) then kept on ice. 6 µL from all three samples were then incubated for 10 seconds at 37C in a 54 µL streptavidin beads aliquot that was pre-equilibrated in D2O (see previous sections). After 10 seconds, the samples were put against a magnetic stand and the supernatant was removed. The beads were then resuspsended in 30 µL protease mix containing 3 µM homemade TEV protease and 1 µL of murine RNAse inhibitor in HKMT buffer, then incubated for 30 mins at room temperature to allow TEV cleaveage and nascent chain elution to occur. Samples were then placed against a magnetic stand and 27 µL of supernatant was removed and mixed with 9 µL of 4x low pH loading dye. All samples were then loaded on a NuPAGe gel which was run in the cold room, and the gel was imagined using a typhoon as described in "SDS-PAGE and anti-bitoin western blot".

<u>Mass spectrometry data analysis</u>

Our basic data analysis pipeline involves the following steps:

1. Peptide identification: Observed MS1 and MS2 spectra from undeuterated controlled samples are assigned to peptides derived from either protein of interest or other proteins in translation + enrichment pipeline.
2. Manual inspection and curation of peptide mass spectra from deuterated samples
3. Fitting of mass spectra to multimodal distributions and identification of optimal # of modes
4. Bootstrapping error analysis

Peptide identification: RAW files from undeuerated control injections, containing precursor and fragment mass spectra (MS1 and MS2 respectively) at all retention times, are loaded Byonic (Protein Metrics) software. For peptide identification we input a FASTA file containing sequences for all *in vitro* translation components present in PURE Express reactions, as well as protein components from nascent chain enrichment steps (namely streptavidin and pepsin) and the translated protein of interest downstream of an AviTag and its respective linker. The following peptide search parameters are used: precursor mass tolerance, 6 ppm; Fragmentation type, QTOF/HCD; Fragment mass tolerance, 20 ppm; Digestion specificity, Non specific; Maximum precursor mass, 10,000; Number of precursors per MS2, 1; Smoothing width (m/z), 0.01. We then retain all peptide + charge state identification associated with a Pep2D score <0.1, and export these spectra and associated retention times for subsequent analysis.
Manual inspection of peptide spectra: RAW files corresponding to all deuterated sampels are loaded into HDExaminer3 (Sierra Analytics) software, alongside peptide lists from previous step and associated retention times. Using HDExaminer, we manually inspect deueterated mass spectra assignemtns for all peptides to ensure that intensity peaks are centered about their expected m/z isotopic windows, and that retention times at which the peptide spectra are observed differ from those in uneuterated spectra by no more than ~0.2 mins. If any retention time shifts are observed for a given peptide, we verify that such a shift is systematically observed across all peptides within that sample. We additionally adjust the retention time windows that are integrated to obtain each peptide's mass spectra to ensure these windows are comparable across samples for that peptide. As an additional quality control step, for each peptide we examine the corresponding m/z and retention time ranges in a negative control sample to rule out interfering or carry-over signal. For each sample, we then discard any peptide spectra for which the negative control signal in relevant m/z windows (namely all isotopic windows ranging from the monoisotopic peak to that largest meaningful window containing signal in the maximally deuterated control) is more than a fifth that within the sample—this indicates that the background is expected to contribute meaningfully to the observed signal. This criterion excludes interference from both background peptides whose spectra overlap with that of the target peptide, as well as carryover; the latter is only observed in a small fraction of peptides. Following manual curation, all resulting high-confidence peptide mass spectra are exported and subsequently fit as described below. These high quality peptides are shown as colored vertical lines in the coverage maps for each protein construct in fig. S2.

Peptide spectra fitting: During (co-translational) protein folding, we expect different structural regions to cooperatively fold and attain protection from deuterium exchange in a time dependent manner. Assuming the deuterium pulse duration is significantly shorter than the timescale

characteristic of the dynamic process of interest (in this case, translation which is coupled to folding), we thus expect our pulse at each translation time to label a diversity of protein conformations that will not significantly interconvert during the time of the pulse itself. At the peptide level, this will result in the appearance of bimodal or multimodal mass spectra—the latter may be observed for larger peptides spanning multiple structural regions that fold asynchronously. For long peptides in which the total number of deuterons taken up in the unfolded state after accounting for back exchange is large, this multimodal behavior can be distinctly seen from the mass spectra (see SI). For each peptide, we thus globally fit all mass spectra from distinct co-translational folding timepoints to a combination of basis spectra indexed by m corresponding to these distinct conformational states, assuming that the centroids themselves are constant across timepoints but that the relative contribution of each bases (its weight) is allowed to vary. That is, for a peptide with charge state z, we assume the mass spectral intensity at mass to charge value m/z and translation time t is given by (

$$I(m/z,t) = A\sum_{m=1}^{N_c} f_t^m \left(\int_0^\infty \frac{dm'}{z}\sum_{i=0}^{N}\left[\delta\left(\frac{m'}{z}-\frac{i}{z}\right)Binom\left(i,N_{eff}^m,p^m\right)\right]\right) * I_0(m/z)$$

Where A describes the signal amplitude related to factors such as digestion and ionization efficiency, N_c is the number of curves being fit, f_t^m is the fraction of signal associated with mode m at time t (at each time, these must sum to 1 over all m), N is the total number of exchangeable amides in the peptide, δ is a Dirac delta function enforcing that all signal peaks must occur at expected isotopic peak, $Binom\left(i,N_{eff}^m,p^m\right)$ refers to a (normalized) binomial distribution evaluated at integer i with parameters N_{eff}^m and p^m (the product of these is the average number of deuterons taken up for mode m), * is the continuous convolution operator, and $I_0(m/z)$ is the natural abundance isotopic distribution for the undeuterated peptide. In the above, we have neglected the mass spectrometric peak width as well as any interference from other peptides.

To analyze data for a given peptide, we globally fit the above equation to mass spectrometric signal at all isotopic peaks across all timepoints, with fitting parameters $A, \{f_t^m\}, N_{eff}^m$, and p^m. The $\{f_t^m\}$ parameters reveal the population fractions associated with each mode at each translation time, while the product $N_{eff}^m p^m$ gives the centroid for mode m. We obtain a better fit by allowing N_{eff}^m to vary rather than insisting it equal the number of exchangeable deuterons in the peptide, as this allows the overall shape of the mode to better adjust to the data. Where technical replicates exist for a given condition, we required that both replicates be fit to the same parameters corresponding to the respective condition. Fitting is implemented in custom Python scripts using the SciPy package. Our approach and fitting algorithm, including our calculation of the natural isotopic distribution, are largely based on those in *(4)*, but adapted to allow for global fitting across timepoints.

To decide on an optimal number of curves N_c, we must balance attaining a high-quality fit with the risk of overfitting. This is achieved in two steps. First, we compute the Bayesian information criterion associated with the fit for each N_c value ranging from 1 to 3 , which we approximate as

$$B(N_c) = n_s \ln\left\{\frac{\sum_{i=1}^{n_s}(\widehat{I_i^{N_c}} - I_i)^2}{n_s}\right\} + k_s \ln\{n_s\}$$

Where the numerator in the first logarithm is the residual sum of squares, obtained by summing the squared differences between the model fit $\widehat{I_i^{N_c}}$ given N_c curves and the experimental intensities peak intensities I_i over data points indexed by i spanning the total number of datapoints for the peptide, n_s, and k_s is the total number of model parameters (excluding the last fraction for each timepoint whose value is constrained by normalization). We note that intensities are normalized per-spectrum prior to being plugged into the above calculation such that timepoints with higher ion abundance are not weighted more heavily. We then determine the smallest number of curves N_B for which the B(N_B) is greater than the smallest B value (across all N_c ranging from 1 to 3) by no more than 10 units. As a second method, we compute the goodness of fit for each fit as

$$R_{N_c}^2 = 1 - \frac{\sum_{i=1}^{n_s}(\widehat{I_i^{N_c}} - I_i)^2}{\sum_{i=1}^{n_s}(I_i - \overline{I_i})^2}$$

Where $\overline{I_i}$ is the average intensity over per-timepoint normalized spectra. From these values we determine N_R which is the smallest value of N_c to achieve $R_{N_c}^2 > 0.95$. Finally, the optimal number of curves is chosen as

$$N_{opt} = \min\{N_B, N_R\}$$

These combined criteria prevent overfitting in a few cases where the Bayesian information calculation favors a larger number of curves than is needed to achieve a satisfactory fit. In SI, we show plots of a few spectra fit to various numbers of curves with an indication of the optimal number per the above criteria, as well as the mathematical justification. In fig. S5, we show fits for three representative peptides from aTS to one, two or three curves as visual evidence that this approach yields a sensible N_{opt} value.The above criteria are applied to the vast majority of peptides and overwritten manually in a limited number of cases, primarily where interfering peaks from other peptides are spuriously fit by the algorithm. Maximally deuterated samples, in contrast, are fit to just one curve in all cases.

Bootstrapping and centroid comparison To obtain error estimates on fitting parameters, we bootstrap by resampling global fit residuals (relative to fit of initial data) with replacement. Residuals are freely shuffled across spectra from different timepoints and technical replicates (where present) for a given peptide; however, we only allow residual exchange between a given pair of datapoints if their respective intensities differ by no more than a factor of 5, to account for the possibility that the noise distribution differs across intensity scales. We also enforce positivity for all resampled intensities. These resampled datapoints are then globally re-fit to the same number of curves determined as per the previous section, and the resulting parameters are recorded. This process is repeated 2000 times for each peptide to obtain distributions and confidence intervals for all parameters, including the number of deuterons for each mode m, computed for each bootstrap iteration as the product $D^m = N_{eff}^m\, p^m$. We discard any bootstrap iterations for which D^m differs from the median value by more than three times the median absolute deviation, as these often represent scenarios where the fit is distorted by spurious peaks due to interfering peptides. We refer to the resulting bootstrapped distribution for the number of deuterons in mode m as $P(\,D^m)$. Separately, we bootstrap the two maximally-deuterated control technical replciates for each sample to obtain a parameter distribution for that condition.

For many analyses, we compare the deuteration of most deprotected (highest D^m) mode with $D^{m,max}$, the number of deuterons in the unfolded state under the null hypothesis that the

former is no smaller than the latter. Extracting $D^{m,u}$ from our maximally deuterated control sample requires correcting for the fact that this sample was prepared via overnight incubation in 90% D2O, whereas pulse HDX-MS samples are deuterated for only 10 seconds. This cannot be trivially corrected experimentally as the presence of 8M urea slows down the rate of exchange rate of exchange in the base-catalyzed regime *(5)*. At pH 6 (read by meter), we find this leads to incomplete labeling of denatured proteins in 10 seconds. Instead, we calculate the fraction of deuterons that we expect to have exchanged in our 10 second pulse for each peptide and scale the number of deuterons taken up in our maximally deuterated control by this fraction. To achieve this, we use a Python implementation of the algorithm in reference *(3)* to compute the expected exchange rates $\{k_i\}$ for all backbone amides across the intact protein sequence. These calculations are performed assuming a temperature of 310 K and pH 5.6. The use of the experimentally-read pH 6 (differing by 0.4 pH unit due to isotope effects on exchange rates) would only increase the number of peptides for which we reject the null, so our conclusions are robust to this choice and, if anything, underestimate the degree of protection.

For a given peptide spanning residues indexed by *a* through *b*, we then define the corrected exchange rates as follows:

$$\{k_i\}_{a \ldots b} = \left.\begin{matrix} 0 \text{ for } i = a, a+1 \\ k_i \text{ otherwise} \end{matrix}\right\}$$

where the first two peptide residues have an effect exchange rate of zero because these two residues back exchange very quickly following digestion (the first residue in fact contains an amine rather than an amide). We then compute the corrected deuteration for the unfolded state as

$$D^{m,corr} = f D^{m,max}$$

where

$$f = \frac{1}{b-a-2}\left(\sum_{i=a+2}^{b} 1 - e^{-k_i t}\right)$$

Where t=10 seconds we have assumed single exponential exchange kinetics for each site. For most peptides, we obtain f values in the range of ~0.9 to 1. We likewise apply this correction to the centroid obtained for all bootstrap iterations performed on the maximally deuterated control to obtain a corrected $P(D^{m,corr})$ for the expected deuteration of an unfolded polypeptide following 10 seconds of pulse labeling under our expeirmnetal conditions. Finally we compute the p-value associated with the null hypothesis that D^m is at least as large as $D^{m,corr}$ as

$$p = \int_0^{\infty} d\, D^{m,corr} P(D^{m,corr}) \int_{D^{m,corr}}^{\infty} dD^m \, P(D^m)$$

For any peptides for which p<0.01 for the least protected mode and for which $D^{m,corr} - D^m \geq 0.5$, we reject the null hypothesis and conclude that the least protected mode is significantly more protected than would be expected if it reflected the unfolded state. This same definition of statistically significant burst-phase protection is also used to designate which peptides are shown with stripped shading in Fig. 2H for aTS. For figures showing mode deuterations normalized by maximally deuterated value, such as Fig. 4G, the confidence intervals are obtained from the distribution of element-wise divided samples from the bootstrapped distribution for the respective mode, and that for the corrected maximally deuterated control, respectively.

We note that, in main text figures showing fractional populations as a function of time and mode protection at each peptide, a subset of high-quality peptides showing poor global fits is

excluded. In addition, in cases where multiple charge states exist for a given peptide, the main text figures only present the charge state showing stronger signal and/or better fitting. the supplementary figures, we present these graphs with all high-quality peptides and redundant charge states included. We observe reproducible behavior across charge states for a given peptide, even though these are fit and analyzed independently (see supplementary figures).

End of translation determination

As a lower bound estimate for the first timepoint associated with the end of translation, we considered, for each timepoint t, the most intense amplitude $A_C^{max,t}$ associated with any peptide/charge state combo spanning the most C-terminal residue for which we have coverage, as well as the least intense amplitude $A_N^{min,t}$associated with any peptide spanning the most N-terminal residue for which we have coverage, and computed their ratio, R_{CN}^t. We then normalized by this intensity ratio for the exact same peptide/charge state pair at the final translation timepoint, $R_{CN}^{t\,final}$, and determined the first timepoint t for which $R_{CN}^t/R_{CN}^{t\,final} > 0.05$—this implies that a nontrivial fraction of nascent proteins are fully translated and that they will appreciably contribute to the detectable mass spectrometric signal at N-terminal peptides. For all proteins, we additionally verify that this timepoint coindicds with appearance of appreciable full-length protein by Western blotting.

Supplementary text

Supplementary Note 1: Evidence that our method detects folding on the ribosome

The control experiments shown in fig. S1D indicate that nascent chain (NC) binding to streptavidin beads under HDX conditions does not disrupt the integrity of NCs. But this experiment does not rule out detection bias in favor of released proteins resulting from subsequent steps in our sample preparation and injection pipeline, including quench or digestion related biases. Here, we provide two lines of evidence pointing against such a bias. Together, these strongly indicate that our MS signal faithfully reports on NCs at timepoints when translation is still occurring.

Evidence 1: Mass spectrometric intensities in CMPK are inconsistent with strong bias in favor of released proteins

Let us consider the alternative hypothesis that our mass spectrometric signal is exclusively sensitive to released proteins, namely either nascent chains that are released prematurely or fully-synthesized, released protein. For CMPK, we observe that roughly ~70% of ribosomes finish translation, as obtained by quantifying the 15 min lane in fig. S3B. Thus, under our **alternative**

hypothesis, the maximum mass spectrometric signal we will be able to detect from nascent chain peptides at co-translational timepoints will be ~30% that at post-translational times, assuming 1) negligible variability in total injected material (see next paragraph) and 2) All peptides whose signal is compared have been fully synthesized by 10 mins; this is likely true for N-terminal peptides on which we focus our attention.

In reality, however, we consistently observe that these two conditions show comparable signal at N-terminal peptides (Table S1). For example, for the core peptide 2-24, z=3, the overall intensities (quantified as the total amplitude associated with the global fits at the respective timepoints) at the 10 and 15 min timepoints nearly identical at 1.8E8 and 1.7E8, respectively. The 10 and 15 min intensities are likewise nearly identical for the lid peptide 40-45 z=2, at 1.2E8 and 1.1E8, respectively. We note that this result holds true when corrected for injection-to-injection variability in total amount of injected material. As a proxy for total injected material, we quantify the intensities associated with peptides from two distinct ribosomal proteins, L5 and L23, as detected in these specific samples. Although our biotin-streptavidin pulldown strategy appreciably enriches for the NC, some signal from ribosomal proteins is nonetheless detected due to nonspecific binding to streptavidin beads and/or co-association with NCs stemming from incomplete ribosomal dissociation during quench.

Although these analyses reveal that the amount of material in the 10 mins sample is slightly greater than that in the 15 mins sample by a factor of 1.5 using the proxy ribosomal protein L5 (Table S2) and a factor of 1.4 when using the proxy protein L23 (Table S3), these ratios are not sufficient to support the alternative hypothesis; the total injected material in the 10 min timepoint would have to be at least ~3.3 times that in the 15 min timepoint in order for the two timepoints to show similar N-terminal CMPK intensities under the hypothesis that no more than 30% of the population (i.e. the spuriously released NCs) is detectable at the 10 mins timepoint.

We additionally note that for lid peptide 40-45, ~60% of this signal at 10 mins is attributed to the protected mode, and the remaining 40% is attributed to a burst phase mode with significant protection (Fig. 2C and fig. S4). Let us consider the related hypothesis that this ~60% detectable protected population consists entirely of spuriously released NCs, which comprise up to 30% of the population. Then this would require that the total amount of injected material associated with the 10 mins timepoint be at least ~2x associated with the 15 mins sample, again larger than the ~1.4x-1.5x factor observed. **This strongly supports that our method can detect co-translational folding in actively translating nascent chains.**

We note that over long timescales (~30 mins to hours), the signal at all peptides, including N-terminal peptides steadily increases, which we attribute to multiphasic biotinylation kinetics.

Evidence 2: Mass spectrometric signal is unlikely to be skewed by nascent-chain-specific precipitation during HDX quench

As a second line of reasoning, we explicitly consider what we believe to be the most likely mechanistic origin for any possible detection bias against nascent chains: namely, selective precipitation of peptidyl-tRNA attached NCs during low-pH quench conditions *(6)*, which could

inhibit digestion of these species while potentially leaving released proteins accessible to digestion. As our quench step is applied to NCs that are physically attached to micron-sized streptavidin beads, we cannot trivially perform a centrifugation-based pelleting assay to quantify the amount of precipitated NCs under our conditions. Instead, we address this hypothesis by systematically increasing the concentration of GdnHCl in our quench buffer from 2M (the concentration used in most of our experiments) to 6M which should significantly reduce precipitation. Thus, if the signal under our standard 2M quench is heavily biased by aggregates, then this [GdnHCl] increase should notably alter the mass spectra we observe. We note that in both cases we reduce the [GdnHCl] to 2M while adding pepsin to ensure the protease can efficiently digest the sample—this complete buffer exchange is uniquely enabled by our bead setup.

Contrary to our hypothesis, we observe highly similar mass spectra for the model protein aTS between two simultaneously-performed pulse-labeling HDX replicates after 8 mins of synchronized elongation—these replicates differ only in their quench concentration as described above. For example, under both quench conditions, peptides 1-7 and 128-152 show deprotection at 8 mins, whereas peptides 35-40 and 85-101 show partial protection. As controls, we also confirm that the 60 mins (post-translational) and maximally deuterated conditions also show similar spectra across both quenches with slightly increased back-exchange when 6M GdnHCl is used. These results strongly suggest that our signal is not appreciably biased against NCs due to acid-induced precipitation under our standard quench conditions.

We note that, formally, we cannot rule out the possibility that some on-bead aggregation occurs amongst bead-attached NCs in our 6M sample following buffer-exchange into the 2M buffer to enable digestion. However, this is extremely unlikely under single-turnover translation conditions where the maximal NC concentration equals the ribosome concentration, which is about 2 μM in PURE systems *(7)*. We estimate that under our experimental conditions assuming this upper-bound concentration, the average spacing between NCs on the beads would be about 16 nm. This spatial isolation strictly constraints macro-aggregation, limiting any possible interactions to low-order oligomerization at most. Moreover, the concentration of NCs is almost certainly much less than 2 μM in our experiments, as per our observation that the total intensity due to translated protein on a Coomassie-stained gel is significantly less than that associated with ribosomal proteins (see fig. S7C for such a gel on HaloTag).

	2-21, z2	2-24, z3	2-26, z3	27-39, z2	40-45, z2	46-67, z4
5mins	3.85E+07	5.22E+07			1.91E+07	
6mins	2.31E+07	6.31E+07	1.01E+07		2.59E+07	1.46E+07
7mins	7.88E+07	1.18E+08	1.64E+07	2.93E+07	7.33E+07	1.53E+07
8mins	6.89E+07	1.20E+08	1.44E+07	2.42E+07	7.70E+07	2.66E+07
10mins	7.79E+07	1.79E+08	3.14E+07	3.82E+07	1.19E+08	5.96E+07

15mins	8.88E+07	1.73E+08	3.11E+07	3.61E+07	1.05E+08	6.16E+07
30mins	1.97E+08	2.81E+08	5.81E+07	7.81E+07	2.64E+08	6.10E+07
2hrs	4.27E+08	6.56E+08	1.09E+08	1.49E+08	5.60E+08	1.90E+08

	58-67, z2	68-78, z2	120-126, z2	127-147, z3	136-147, z2	148-162, z4
5mins	1.52E+07					
6mins	1.93E+07	7.17E+06				
7mins	1.10E+08		1.03E+07	3.46E+06		
8mins	1.18E+08	1.48E+07	2.45E+07	2.03E+07	2.04E+07	
10mins	1.65E+08	3.25E+07	7.40E+07	9.84E+07	1.09E+08	5.66E+06
15mins	1.69E+08	3.82E+07	9.68E+07	1.99E+08	1.92E+08	2.52E+07
30mins	4.58E+08	5.42E+07	2.71E+08	3.78E+08	6.80E+08	2.95E+07
2hrs	1.07E+09	1.52E+08	8.21E+08	7.74E+08	1.90E+09	1.19E+08

	175-200, z5	207-218, z2	208-218, z2	211-218, z2
5mins				
6mins				
7mins				
8mins				
10mins	2.04E+06		1.72E+06	
15mins	3.40E+07		3.08E+07	6.49E+06
30mins	6.81E+07	1.93E+07	1.17E+08	4.04E+07
2hrs	2.24E+08	7.40E+07	4.19E+08	1.44E+08

Table S1.
Global fit amplitude, a quantification of overall mass spectrometric intensity, as a function of translation time for indicated CMPK peptides. No value indicates the peptide was not detected at the indicated time

	20-36, z3	37-57, z3	139-153, z4	139-153, z3	171-179, z3
8mins	6.80E+08	4.93E+09	1.51E+09	7.77E+08	1.43E+09
10mins	5.40E+08	4.24E+09	1.57E+09	7.45E+08	1.48E+09
15mins	4.29E+08	3.31E+09	9.37E+08	4.61E+08	9.88E+08

30mins	4.29E+08	3.10E+09	7.49E+08	4.04E+08	9.08E+08

Average 10min to 15min ratio	1.46585922

Table S2.

Global fit amplitude, a quantification of overall mass spectrometric intensity, as a function of translation time for indicated peptides from ribosomal protein L5 during translation reaction of CMPK. Only a subset of translation times are shown. Final row shows average ratio of intensity at 10 mins to that at 15 mins over all peptides.

	1-7, z2	1-7, z3	8-29, z4	8-29, z5	30-50, z5	33-50, z4	33-50, z5
8mins	6.30E+07	6.30E+07	8.96E+07	1.03E+08	7.84E+07	6.80E+07	4.17E+07
10mins	6.20E+07	7.17E+07	9.13E+07	1.02E+08	8.88E+07	7.60E+07	6.32E+07
15mins	5.74E+07	5.87E+07	7.19E+07	6.17E+07	7.73E+07	5.19E+07	3.16E+07
30mins		6.85E+07	4.76E+07	6.08E+07	3.79E+07	5.01E+07	3.99E+07

Average 10min to 15min ratio	1.40545405

Table S3.

Global fit amplitude, a quantification of overall mass spectrometric intensity, as a function of translation time for indicated peptides from ribosomal protein L23 during translation reaction of CMPK. Only a subset of translation times are shown. Final row shows average ratio of intensity at 10 mins to that at 15 mins over all peptides.

<u>Supplementary Note 2:</u> Altered HaloTag folding sequence cannot be accounted for by lagging ribosomes

We note that peptides from N-terminal regions will generally appear to fold more slowly in our assay than peptides from the C-terminus, even if the regions fold simultaneously in reality, as lagging ribosomes will present as a population of unfolded N-terminal peptides that is slow to vanish. However, this effect is not sufficient to account for our observation that newly-translated

HaloTag folds via a different intermediate solely involving the C-terminus, in contrast to refolding from denaturant which involves an intermediate that includes both termini. Below, we provide two lines of evidence supporting this:

1. During refolding from denaturant, the native folding of peptides 274-285 lags that of peptide 286-294 by over 5 mins (Fig. 3E). If newly-translated HaloTag folded via the exact same mechanism and similar kinetics upon release from the ribosome, than we would expect 274-285 to lag peptide 286-293 (which reports on nearly the same region as 286-294) by at least 5 minutes. But in reality, these fold simultaneously immediately upon release from the ribosome (Fig. 3C).
2. During refolding, peptide 129-144 folds slightly more rapidly than peptide 263-273. If newly-translated HaloTag folded via the exact same mechanism upon release from the ribosome, then at 20 mins translation time, we would expect to see at least 45% native population of peptide 145-151 (which reports on nearly the same region as 145-152) at 20 mins, at which point peptide 265-273 (which reports on nearly the same region as 263-273) is ~60% folded. Namely, quantification of the gel in fig. S7B indicates that at 20 mins, at least ~75% of ribosomes have finished translation—in reality it is likely more as the full-length band is overloaded. Thus if we assume that all folded populations stems entirely from fully-translated proteins, then the expected folded fraction for 131-144 is at least 0.6*0.75 = 0.45 (likely larger than this as 145-151 folds slightly more rapidly than 265-273 in our null model). But in reality, 131-144 is only about 20% folded at this time.

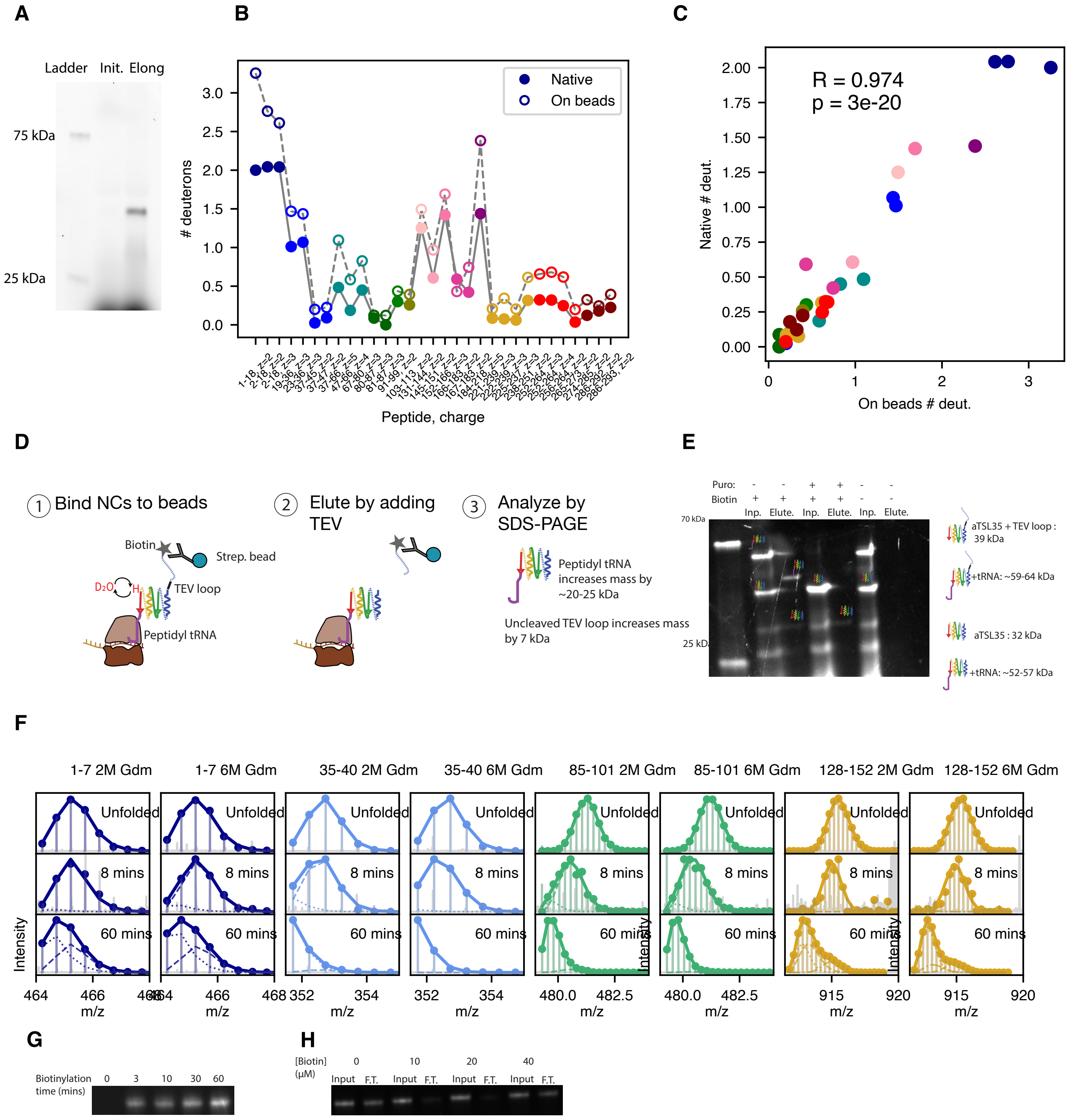
A
Ladder
Init.
Elong
75 kDa
25 kDa
B
Native
On beads
deuterons
Peptide, charge
C
R = 0.974
p = 3e-20
Native # deut.
On beads # deut.
D
1 Bind NCs to beads
2 Elute by adding TEV
3 Analyze by SDS-PAGE
Biotin
Strep. bead
TEV loop
Peptidyl tRNA
Peptidyl tRNA increases mass by ~20-25 kDa
Uncleaved TEV loop increases mass by 7 kDa
E
Puro:
Biotin
Inp.
Elute.
70 kDa
25 kDa
aTSL35 + TEV loop : 39 kDa
+tRNA: ~59-64 kDa
aTSL35 : 32 kDa
+tRNA: ~52-57 kDa
F
1-7 2M Gdm
1-7 6M Gdm
35-40 2M Gdm
35-40 6M Gdm
85-101 2M Gdm
85-101 6M Gdm
128-152 2M Gdm
128-152 6M Gdm
Unfolded
8 mins
60 mins
Intensity
m/z
G
Biotinylation time (mins)
0
3
10
30
60
H
[Biotin] (μM)
0
10
20
40
Input
F.T.

fig. S1. Validation of single-turnover translation and HDX-MS enrichment protocols

(A) Addition of 75 μM aurintricarboxylic acid (ATCA) at the elongation step leads to single-turnover translation. A synchronized translation reaction of aTS with Fluorotect GreenLys was treated with ATCA at either the initiation or elongation step, resolved via SDS-PAGE, and visualized by fluorescent imaging. Full-length aTS product is observed only when ATCA is added at the elongation step (right lane, "Elong.") but not at the initiation step (left lane, "Init."), indicating that ATCA blocks translation initiation without inhibiting actively elongating ribosomes. **(B)** Nascent protein enrichment coupled to HDX-MS does not globally disrupt protein structure. Pulse HDX-MS was performed on a HaloTag sample after 4 hours of elongation, enriched via biotin/streptavidin affinity capture. The number of deuterons accumulated per peptide in the protected mode (empty markers) is compared to a standard 10-second pulse-labeled HDX control on purified HaloTag protein (filled markers). **(C)** Correlation between peptide deuteration levels for the two experiments shown in (B). The high Pearson correlation coefficient and low p-value indicate a strong linear correlation. The slope of less than 1 indicates that deuterium uptake under enrichment conditions is systematically increased across the protein by a constant factor, suggesting that while the enrichment protocol (biotinylation and streptavidin bead binding) slightly destabilizes the protein, it does not globally alter its folded structure. **(D)** Schematic of protocol for assessing nascent chin (NC) integrity following streptavidin bead binding. We translate a stalled NC construct with an N-terminal AviTag separated from the protein of interest by a linker containing a TEV loop. Following hydrogen-deuterium exchange and bead binding, TEV protease is added to elute NCs from beads, and samples are analyzed by SDS PAGE. If NCs are ribosomally attached upon elution, then they will migrate ~20-25 kDa larger than expected due to peptidyl tRNA attachment, a migration shift that vanishes if NCs are deliberately released from the ribosome via puromycin addition. For details, see Materials and Methods **(E)** SDS-PAGE gel, imaged via fluorescence of NCs containing FluorTect GreenLYs (see Methods), showing input (prior to bead binding) and elution (post TEV addition) samples as described in (D). Puromycin is added prior to bead binding, and biotin is included in IVT reactions, as indicated. We observe elutants migrate ~20-25 kDa larger in the absence of puromycin than in its presence, indicating peptidyl tRNA attachment, assuming biotin is added to the translation reaction. The absence of biotin precludes streptavidin bead binding, and thus no elutants are observed. We note that elutants migrate 7 kDa faster than their respective input samples due to loss of N-terminal linker following TEV loop cleavage. **(E)** Mass spectra (represented as described in main text Fig. 2B) of various aTS-derived peptides derived from maximally deuterated control ("Unfolded") , 8 mins translation timepoint, and 60 mins translation timepoint following pulse-labeling HDX and bead binding reaction quenched with low pH buffer containing either 2M or 6M GdnHCl. The high degree of similarity between trends observed in the spectra from these two conditions both during mid-translation (8 mins) and post-translation (6 mins) strongly suggests against the possibility that our observed signal is biased towards spuriously released NCs due to acid-induced precipitation of peptidyl-tRNA attached species, as if precipitation occurs, then increasing [GdnHCl] from 2 to 6M should significantly reduce its extent and thus affect the observed signal. We note that the 6M GdnHCl quench leads to slightly increased back exchange, as observed in respective maximally deuterated control samples. (**F**) Biotinylation timecourse: The protein HaloTag with an N-terminal AviTag and C-terminal 35 AA linker was translated *in vitro* in the presence of 20

μM biotin for 1.5 hours, then was supplemented with 75 μM ATCA (to prevent further intiaiton) and 1 μM BirA to begin biotinylation. At indicated times (including t=0, immediately prior to addition of BirA), a 4 μL reaction aliquot was drawn and translation was quenched with 18 μM puromycin at room temperature for 30 seconds, and samples were then mixed with Lamelli dye at 1X final concentration and immediately incubated at 95C for 5 mins to fully inactive BirA. All samples were then loaded on an SDS-PAGE gel and transferred to a PVDF membrane for anti-biotin western blotting with an Alexa-488 conjugated streptavidin probe. We conclude that nascent chain biotinylation is multiphasic, with a substantial degree of biotinylation occurring within a few minutes, indicating it is possible to appreciably enrich for nascent chains at early translation times. However, complete biotinylation requires hour timescales (H) Determination of optimal biotin concentration for nascent chain binding to streptavidin beads. The protein HaloTag with an N-terminal AviTag and C-terminal 35 AA linker was translated *in vitro* for 1 hour at 37C in the presence FluoroTect and variable biotin concentrations as indicated. Subsequently, 2 μL of each sample was drawn as an "input sample", diluted in HKMT for a total volume of 8 μL, and frozen, then the remaining volume was bound for 2 hours with rotary mixing to a 15 μL aliquot of Pierce Streptavidin beads pre-equilibrated in HKMT. The flow-through was removed, and 8 μL of flow-through (F.T.) was drawn for gel analysis—we note that, if we assume no bead binding, this 8 μL aliquot would contain the same quantity of nascent chains as the input sample. All 8 μL samples were then treated with RNAse A to 1 mg/ml, followed by addition of Laemmli buffer to 1X concentration. Samples were then analyzed on a NUPAGE 4-12% Bis Tris gel and FluoroTect fluorescence was imaged. We conclude the 20 μM biotin corresponds to the optimal concentration for bead binding—increasing to 40 μM leads to reduced bead binding due to free biotin competing with streptavidin binding sites.

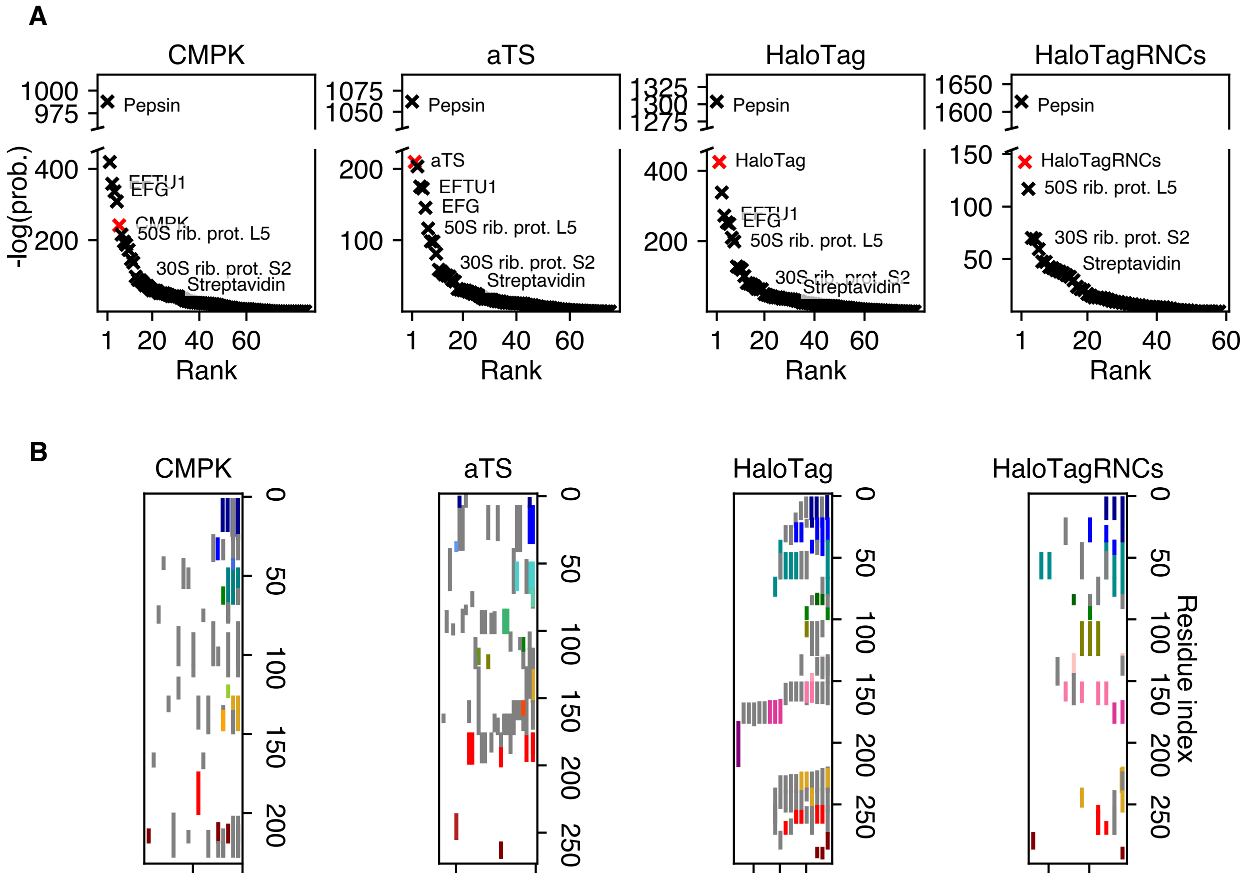

fig. S2. Nascent chain enrichment and coverage

(A) For each protein of interest that we analyze in this work (indicated above respective plots), we show confidence associated with identification of various protein species derived from our *in vitro* translation mixture, as well as proteins from our enrichment + digestion pipeline. Protein identification confidence are ranked by negative log p-values, with higher values indicating a lower probability of false assignment by the Byonic search algorithm. In each plot, the red marker indicates the protein of interest, while other proteins are shown in black. The positions corresponding to various proteins are annotated including pepsin (used for digestion), elongation factors EFTU1 and EFG, two ribosomal proteins L5 and S2, as well as streptavidin. For all proteins except CMPK, the protein of interest produces the highest confidence identification other than pepsin, which is added to bead slurry at high levels for digestion. The score corresponding to CMPK is slightly lower due to a lower digestion efficiency for this protein, but nonetheless high compared to the majority of proteins that were originally present in the non-enriched sample. HaloTagRNCs refers to a HaloTag + 34 AA C-terminal linker containing a strong SecM stalling sequence purified by sucrose cushion ultracentrifugation, which de-enriches

non-ribosomal proteins from this sample. **(B)** Coverage maps indicating high confidence peptides detected from the indicated proteins. Each vertical bar represents a unique peptide + charge state combination that spans the residues indicated on the right axis provided its corresponding Pep2D value (a confidence metric used by Byonic) is less than 0.1, and colored bars represent high quality peptides used for HDX-MS analysis. We note that these figures show representative coverage maps from a single replicate; the exact coverage varies slightly from replicate to replicate.

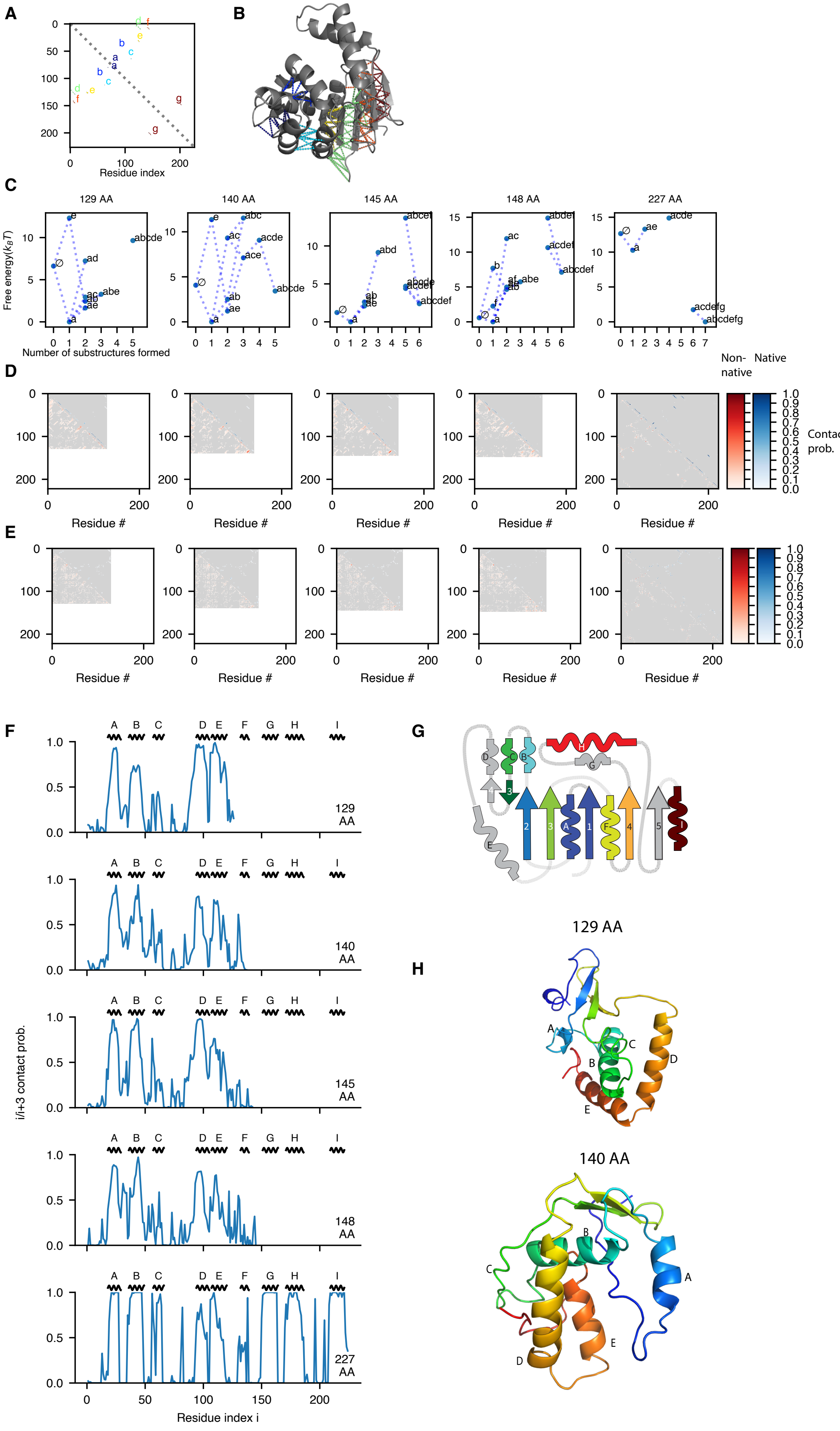
A
B
C
D
E
F
G
H
Residue index
129 AA
140 AA
145 AA
148 AA
227 AA
Free energy(k_BT)
Number of substructures formed
Non-native
Native
Contact prob.
Residue #
i/i+3 contact prob.
Residue index i
129 AA
140 AA

fig. S3 DBFOLD simulations predict CMPK NMP subdomain folds co-translationally while core folding is delayed until after translation

Replica-exchange Monte-Carlo simulations with native-contact centric umbrella biasing using the MCPU method from reference *(8)* were reanalyzed as follows: **(A)** CMPK native contact map substructures, defined as in *(8),* shown in color and labeled. Each substructure defines a set of contiguous native contacts that is expected to form cooperatively during protein folding. For details on this substructure approach, see *(9)* **(B)** CMPK substructures mapped onto the native structure—each dash is a native contact belonging to a substructure color-coded as in (A). **(C)** Potentials of mean force (PMFs) showing free energy as a function of topological configuration—defined as a state in which a given subset of substructures is formed or absent--for CMPK constructs in which various numbers of amino acids (AAs) have been synthesized. This analysis was performed using a slightly more lenient *f* parameter (defined as the maximum allowable ratio of the average distance between contacting residues in a given snapshot, to that same distance in the native state, such that a substructure involving those contacts is deemed present in the snapshot) to more accurately capture the presence of dynamic inter-helix contacts. For details on this approach, see *(9)*. These PMFs are shown at a simulation temperature of T=0.5 (in arbitrary simulation energy units), at which point the folding free energy at full length (227 AA) is about 10 k_BT, comparable to the experimentally-measured value of 6.4 kcal/mol in *(10)* **(D)** Average alpha-carbon contact maps, computed using the MBAR method *(11)*, as a function of translation length at a simulation temperature of T=0.5. Two alpha-carbons are deemed in contact in a given snapshot if their distance is within 6 Angstroms. The color shading reflects contact probability (as in colorbar shown) and blues in the upper triangular region reflect native contacts, while nonnative contacts are shown in shades of red in the lower triangular region. For details on this approach, see *(12)*. This analysis reveals that native N-terminal alpha-helices form co-translatioanlly with high probability, but native = beta sheets (with the exception of beta hairpin reflected by substructure *a* in previous panels) do not form until translation is complete. **(E)** Same as (D) but now showing average side-chain contact maps, with a distance cutoff threshold of 5 Angstroms for defining a contact. These contact maps reveal a diversity of interconverting, nonnative tertiary packings at intermediate lengths, while stable, persistent native contacts do not form until full length as reflected by the dark shades of blue at length 227. **(F)** The third diagonal from the upper right triangular region in panel (D) is extracted at each chain length and plotted to reveal the probability of alpha helical contacts between each residue pair i and i+3. The locations for native alpha helices (labeled as in main text Fig. 2) are indicated above the panels. According to this analysis, DBFOLD simulations predict a high probability of NMP subdomain helix formation at intermediate chain lengths while core helices form post-translationally. The exception is helix A, which is predicted to form co-translationally despite being part of the core—this is in disagreement with experimental results and may reflect an artificial over-stabilization of this helix in the simulation forcefield. **(G)** Topology map for CMPK with secondary structures labeled and color coded as in Fig. 2. **(H)** Sample simulation snapshots from indicated lengths with helices lableed. Note that these snapshots are shown in chainbow coloring that does not match the coloring shown in panel G.

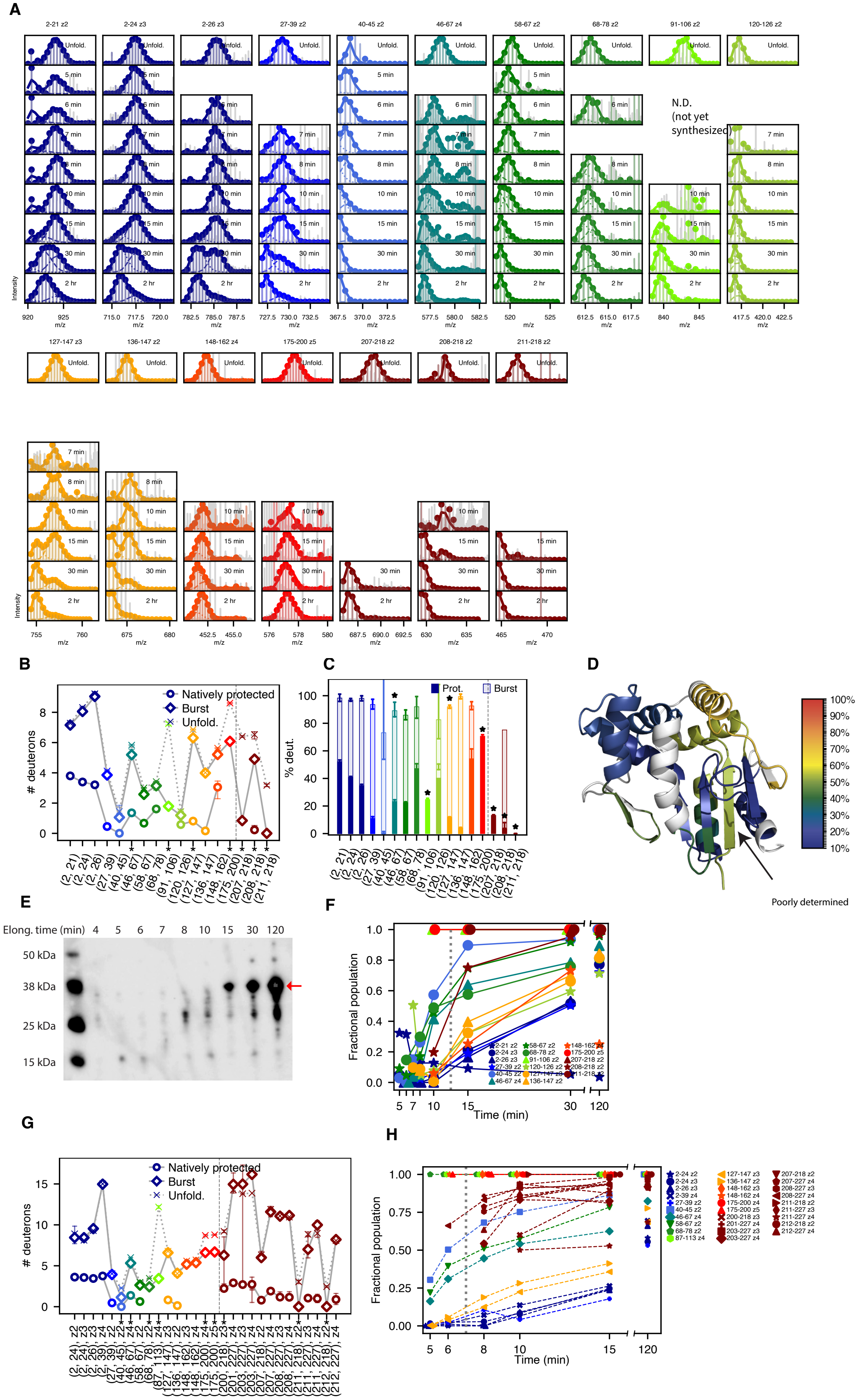
A
2-21 z2
2-24 z3
2-26 z3
27-39 z2
40-45 z2
46-67 z4
58-67 z2
68-78 z2
91-106 z2
120-126 z2
Unfold.
5 min
6 min
7 min
8 min
10 min
15 min
30 min
2 hr
N.D.
(not yet
synthesized)
Intensity
m/z
127-147 z3
136-147 z2
148-162 z4
175-200 z5
207-218 z2
208-218 z2
211-218 z2
B
Natively protected
Burst
Unfold.
deuterons
C
Prot.
Burst
% deut.
D
100%
90%
80%
70%
60%
50%
40%
30%
20%
10%
Poorly determined
E
Elong. time (min)
4
5
6
7
8
10
15
30
120
50 kDa
38 kDa
25 kDa
15 kDa
F
Fractional population
Time (min)
G
Natively protected
Burst
Unfold.
deuterons
H
Fractional population
Time (min)

fig. S4 CMPK synchronized translation + pulse-labeling HDX-MS
(A) Mass spectra for all CMPK peptides derived from unfolded control (top row) and all elongation times, as in main text Fig. 2B. Empty panels reflect lack of peptide signal, either because the peptide is not yet synthesized or due to stochastic variabilities in mass-spectrometric detection for low-abundance peptides. **(B)** Number of deuterons associated with natively protected mode (circles), burst-phase mode (the deprotected mode observed early in translation, diamonds) and unfolded state (Xs) for each peptide. Unfolded deuteration are corrected for 10 second labeling (see Materials and Methods). Error bars represent 95% confidence intervals from bootstrapping and asterisks denote peptides exhibiting statistically significant burst-phase protection (see Methods). Peptides to the right of the dashed line are only detected after 10 mins (post-translation). **(C)** Same data as (B) but with deuterations expressed as a percentage of maximally deuterated value. **(D)** Percentage deuterations of all peptides are mapped onto the CMPK structure (PDB ID *2cmk*). In general, protection from deuteration correlates with secondary structure. Beta strand 1 appears to show anomalously high deuteration as it is only spanned by large peptides that encompass significant disordered regions, and hence its deuteration cannot be accurately determined. **(E)** Anti-biotin western blot (as in main text Fig. 1C.) showing progress of CMPK synchronized translation reaction as a function of time. This blot reflects the replicate that was used for pulse-labeling experiments shown in main text Fig. 2 and panel (A). Red arrow indicates full-length protein. At around 8 mins, we observe appreciable accumulation of a ~26 kDa translation intermediate, which corresponds to translation of roughly 180 AA from CMPK (with additional ~6 kDa due to the N terminal AviTag + linker). At this stage, the lid subdomain (residues 36-120) are fully released from the ribosome. Our observation that lid folding begins around this time (Fig. 2C) strongly suggests that this subdomain folds shortly after its emergence from the ribosome. **(F)** Same as main text Fig. 2C, now showing all peptides with high quality mass spectra and replicate charge states where present **(G)** Same as (B) for replicate CMPK synchronized translation reaction. (**H)** Same as main text Fig. 2C for a replicate CMPK synchronized translation reaction. As in the first replicate, we observe that lid peptides synchronously begin acquiring protection prior to completion of synthesis, followed by post-translational core folding and rapid NMP subdomain folding.

Peptide: (50, 69), Charge: 3.0

A

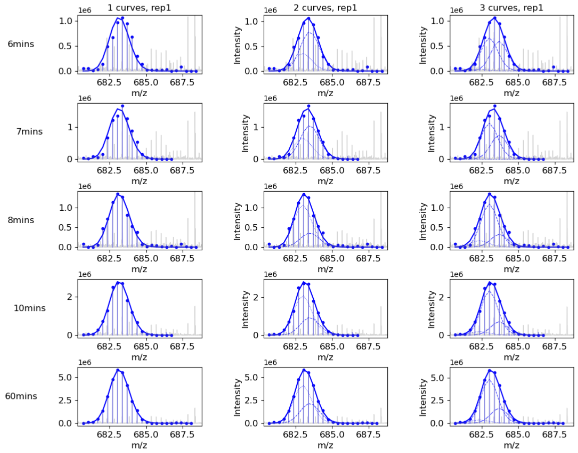

ncurves	RSS	R^2	k	n	BIC
1	0.011483	0.972820	7	95	-825.096922
2	0.004037	0.990444	14	95	-892.528740
3	0.003607	0.991462	21	95	-871.352148

opt n curves = 1

B

Peptide: (35, 40), Charge: 2.0

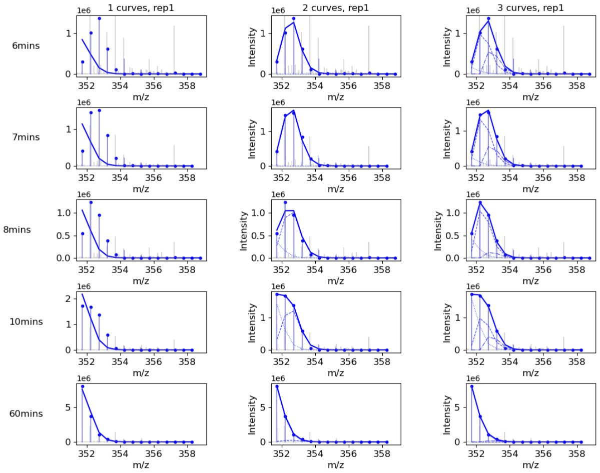

ncurves	RSS	R^2	k	n	BIC
1	0.922705	-0.259103	7	30	-80.640907
2	0.011201	0.984716	14	30	-189.172831
3	0.003223	0.995602	21	30	-202.733914

opt n curves = 2

Peptide: (85, 101), Charge: 4.0

C

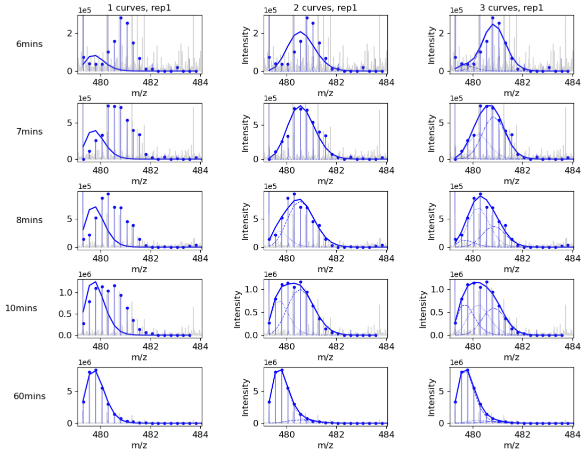

ncurves	RSS	R^2	k	n	BIC
1	0.621762	-0.570887	7	80	-357.903718
2	0.045262	0.885646	14	80	-536.837308
3	0.011983	0.969725	21	80	-612.480767

opt n curves = 3

fig. S5 Determination of optimal number of curves for fitting aTS peptides. **(A)-(C)** For indicated peptides and charge states, we globally fit mass spectra from aTS synchronized translation experiments to either one, to or three curves. Mass spectra for each translation time and number of curves, alongside fits, are shown as in main text Fig. 2B. To the right of each set of plots, we indicate the RSS (residual sum of squares), R^2 (goodness of fit), k (number of free fitting parameters), n (number of fitted datapoints) and BIC (Bayesian information criterion) associated with each number of curves, as well as the optimal number, determined as described in Materials and Methods.

A

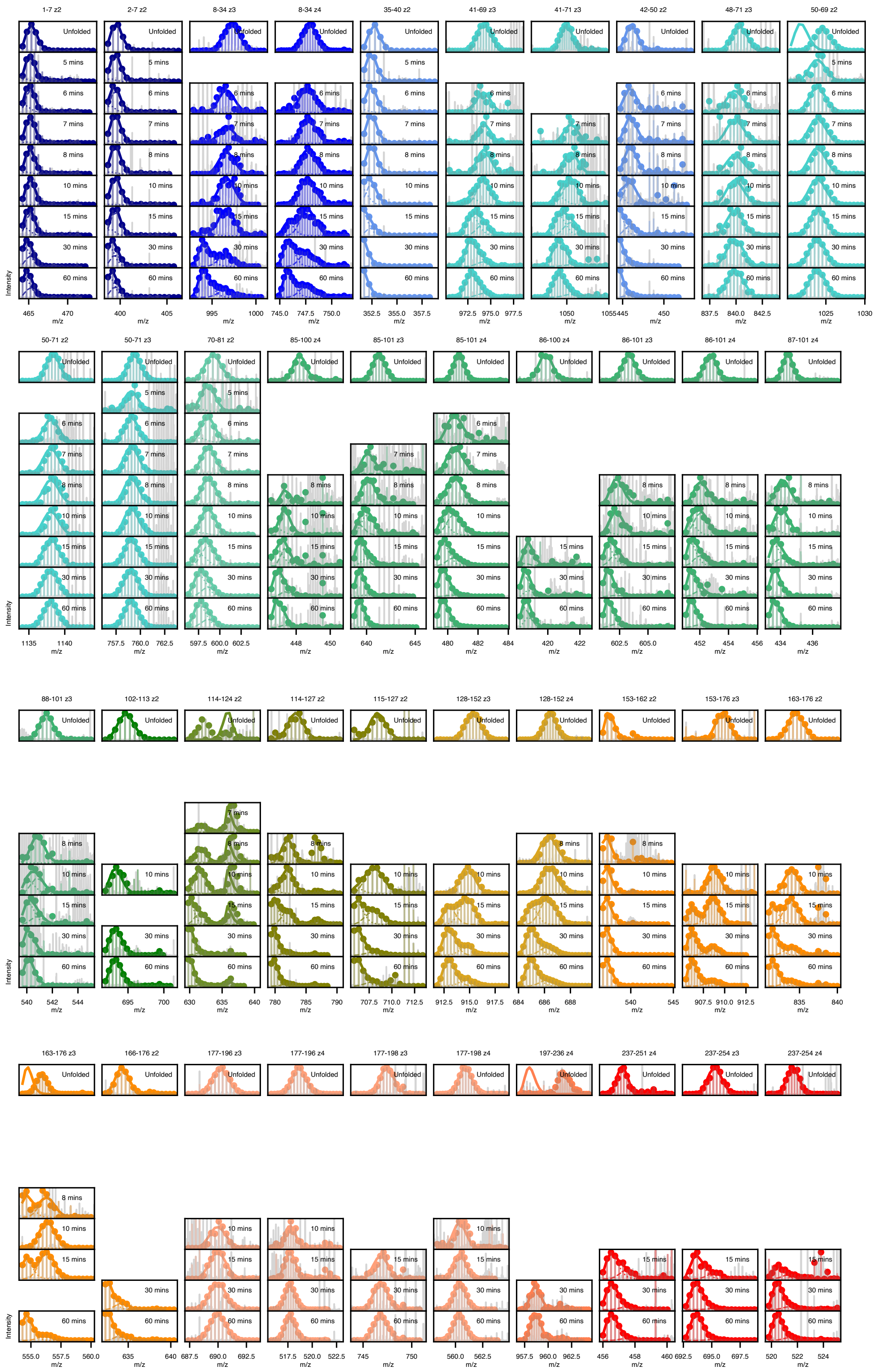
1-7 z2
2-7 z2
8-34 z3
8-34 z4
35-40 z2
41-69 z3
41-71 z3
42-50 z2
48-71 z3
50-69 z2
50-71 z2
50-71 z3
70-81 z2
85-100 z4
85-101 z3
85-101 z4
86-100 z4
86-101 z3
86-101 z4
87-101 z4
88-101 z3
102-113 z2
114-124 z2
114-127 z2
115-127 z2
128-152 z3
128-152 z4
153-162 z2
153-176 z3
163-176 z2
163-176 z3
166-176 z2
177-196 z3
177-196 z4
177-198 z3
177-198 z4
197-236 z4
237-251 z4
237-254 z3
237-254 z4
Unfolded
5 mins
6 mins
7 mins
8 mins
10 mins
15 mins
30 mins
60 mins
Intensity
m/z

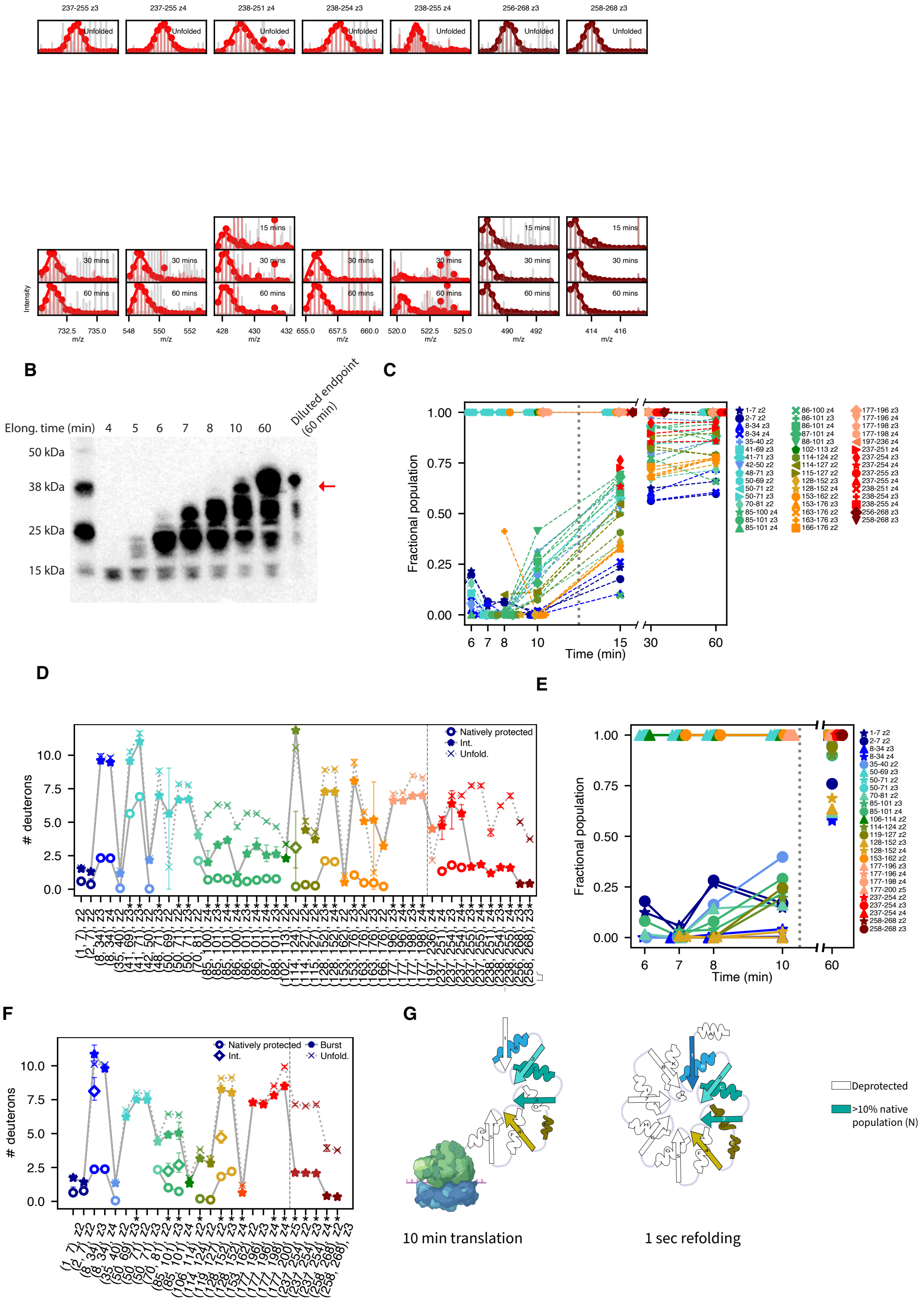
237-255 z3
237-255 z4
238-251 z4
238-254 z3
238-255 z4
256-268 z3
258-268 z3
Unfolded
15 mins
30 mins
60 mins
Intensity
m/z
B
Elong. time (min)
4
5
6
7
8
10
60
Diluted endpoint (60 min)
50 kDa
38 kDa
25 kDa
15 kDa
C
Fractional population
Time (min)
D
deuterons
Natively protected
Int.
Unfold.
E
Fractional population
Time (min)
F
deuterons
Natively protected
Int.
Burst
Unfold.
G
10 min translation
1 sec refolding
Deprotected
>10% native population (N)

fig. S6 aTS synchronized translation + pulse-labeling HDX-MS

(A) Mass spectra for all aTS peptides derived from unfolded control (top row) and all elongation times, represented as in main text Fig. 2B. Empty panels reflect lack of peptide signal, either because the peptide is not yet synthesized or due to stochastic variabilities in mass-spectrometric detection for low-abundance peptides. **(B)** Anti-biotin western blot (as in main text Fig. 1C.) showing progress of CMPK synchronized translation reaction as a function of time. This blot reflects the replicate that was used for pulse-labeling experiments shown in main text Fig. 2 and panel (A). Red arrow indicates full-length protein. (**C)** Same as main text Fig. 2G, now showing all peptides with high quality mass spectra and replicate charge states where present. **(D)** Number of deuterons associated with natively protected mode (circles), intermediately protected mode where present (stars), burst-phase mode (the deprotected mode observed early in translation, diamonds) and unfolded state (Xs) for each peptide. Unfolded deuteration are corrected for 10 second labeling (see Materials and Methods). Error bars represent 95% confidence intervals from bootstrapping and asterisks denote peptides exhibiting statistically significant burst-phase protection (see Methods). Peptides to the right of the dashed line are only detected after 10 mins (post-translation). Various aTS peptides optimally fit to a single mode, For these peptides, only a star and x are shown, representing deuteration of the single global fit mode and the unfolded mode, respectively. **(E)** Same as panel C for a replicate aTS synchronized translation reaction. **(F)** Same as (D) for replicate aTS synchronized translation reaction **(G)** Comparison of folding intermediates populated during aTS translation and refolding from denaturant. Left panel shows secondary structures spanned by peptides showing a natively protected fractional population of at least 10%,, provided the deuterium uptake in this native mode is no more than 50% that in the maximally deuterated control, as in main text Fig. 2H, while right panel shows structures spanned by at least one peptide showing a natively protected fractional population of at least 10% at 1 second of refolding from denaturant in the pulse-labeling HDX-MS experiment from Wintrode et. al. 2005 *(13).* For numerical values, see table S4. The co-translational and refolding intermediates largely resemble each other with the exceptions of beta strand 1 as well as the small helix D, which are protected during refolding but not in the co-translational intermediate.

Peptide	Charge	Native Fraction (10 min translation)	Native Fraction (1 sec refolding)
**(1, 7)**	2	0	0.04
**(2, 7)**	2	0.02	N.D.
**(8, 11)**	N.S.	N.D.	0.05
**(8, 34)**	3	0	N.D.
**(8, 34)**	4	0.02	N.D.
**(12, 19)**	N.S.	N.D.	0.22
**(20, 34)**	N.S.	N.D.	0.46

**(35, 40)**	2	0.31	0.44
**(40, 46)**	N.S.	N.D.	0.31
**(41, 49)**	N.S.	N.D.	0.44
**(41, 69)**	3	0.21	N.D.
**(41, 71)**	3	0.22	N.D.
**(42, 50)**	2	0.19	N.D.
**(48, 71)**	3	1	N.D.
**(50, 65)**	N.S.	N.D.	N.D.
**(50, 69)**	2	1	N.D.
**(50, 71)**	2	1	N.D.
**(50, 71)**	3	1	N.D.
**(70, 81)**	2	0.08	0.16
**(82, 86)**	N.S.	N.D.	0.79
**(85, 100)**	4	0.22	N.D.
**(85, 101)**	3	0.26	N.D.
**(85, 101)**	4	0.31	N.D.
**(86, 101)**	4	0.26	0.43
**(86, 101)**	3	0.08	0.43
**(87, 101)**	4	0.16	N.D.
**(88, 101)**	3	0.41	N.D.
**(102, 105)**	N.S.	N.D.	N.D.
**(102, 113)**	2	1	N.D.
**(106, 113)**	N.S.	N.D.	N.D.
**(114, 118)**	N.S.	N.D.	0.15
**(114, 124)**	2	0.07	N.D.
**(114, 127)**	2	0.12	N.D.
**(115, 124)**	N.S.	N.D.	0.11
**(115, 127)**	2	0.11	N.D.
**(128, 134)**	N.S.	N.D.	N.D.
**(128, 152)**	3	0	0.06
**(128, 152)**	4	0	0.06
**(153, 162)**	2	1	N.D.
**(153, 176)**	3	0	N.D.
**(163, 172)**	N.S.	N.D.	0.07
**(163, 176)**	3	0	0.07
**(163, 176)**	2	0	0.07
**(177, 196)**	4	1	N.D.
**(177, 196)**	3	1	N.D.
**(177, 198)**	4	1	0.03

**(199, 236)**	N.S.	N.D.	0.1
**(237, 251)**	N.S.	N.D.	0.04
**(258, 268)**	N.S.	N.D.	0.05

Table S4: aTS co-translational folding intermediate resembles refolding intermediate from denaturant. For each peptide and charge state, we indicate the fraction protected in both the 10 minute co-translational timepoint (our experiment) and the 1 second refolding timepoint (Wintrode et. al. 2005) *(13).* N.D. indicates peptide is not detected in the respective experiment, and N.S. indicates that the charge state was not specified for peptides in Wintrode et. al. 2005.

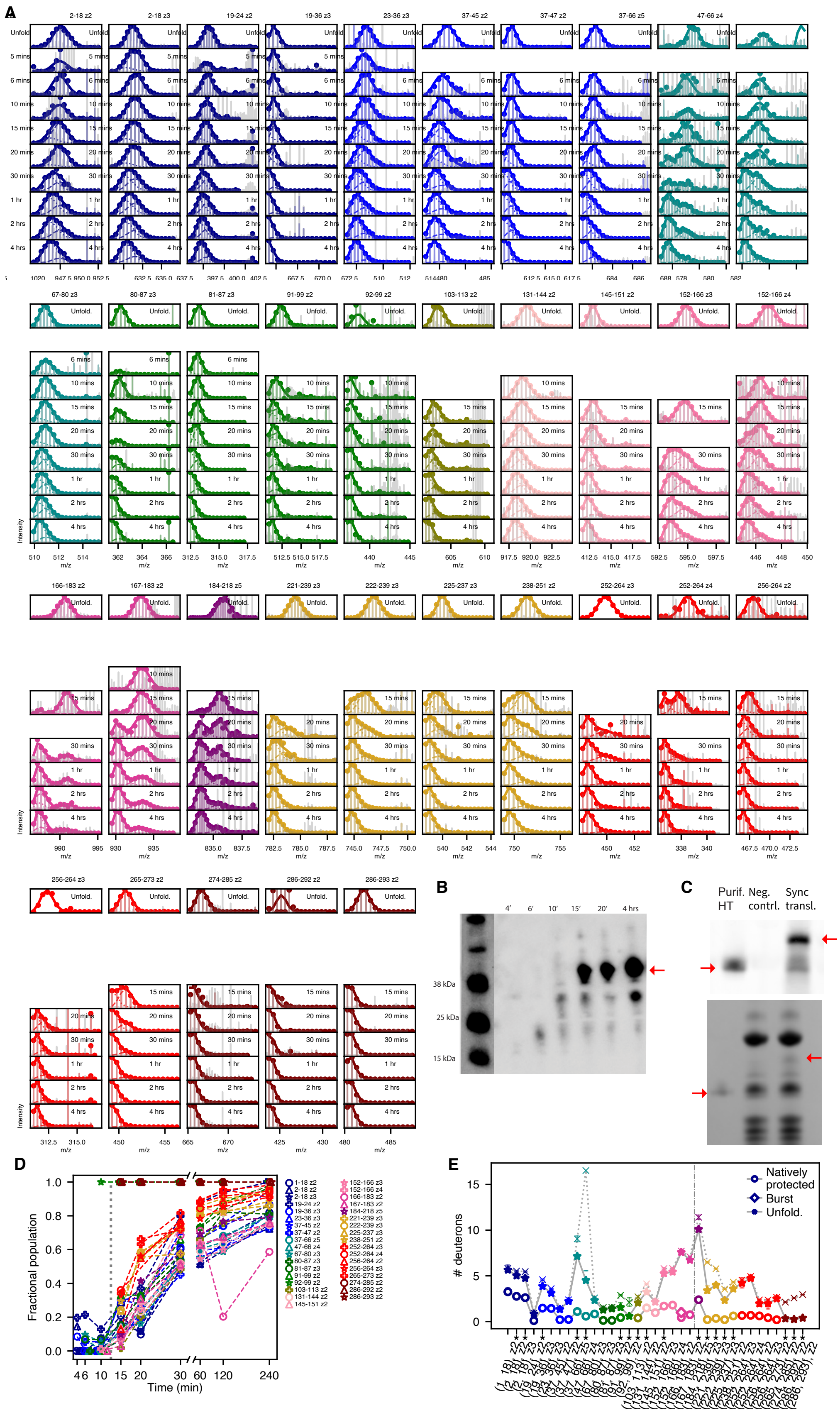

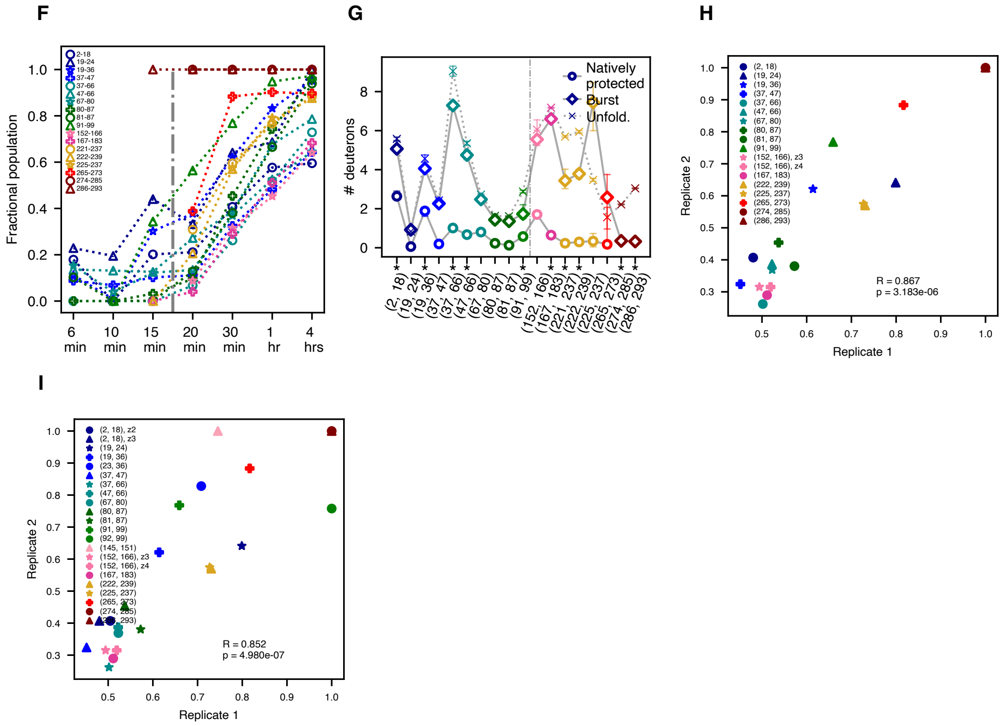

fig. S7: HaloTag synchronized translation + pulse-labeling HDX-MS
(A) Mass spectra for all HaloTag peptides derived from unfolded control (top row) and all elongation times, represented as in main text Fig. 2B. Empty panels reflect lack of peptide signal, either because the peptide is not yet synthesized or due to stochastic variabilities in mass-spectrometric detection for low-abundance peptides. **(B)** Anti-biotin western blot (as in main text Fig. 1C.) showing progress of HaloTag synchronized translation reaction as a function of time. This blot reflects the replicate that was used for pulse-labeling experiments shown in main text Fig. 2 and panel (A). Red arrow indicates full-length protein. **(C)** Top: To verify HaloTag folds into its native structure in our experimental conditions, we perform a synchronized translation reaction in the presence of 10 µM of fluorescent HaloTag ligand TMR, and load the final timepoint on an SDS-PAGE gel which we fluorescently image to confirm covalent TMR bidning (middle lane). As a negative control, we perform an equivalent translation reaction without the HaloTag gene (rightmost lane), and we also load purified HaloTag that was similarly incubated with 10 µM TMR as a positive control (leftmost lane). Translated HaloTag runs larger than purified protein due to the added presence of the N-terminal AviTag and linker. Truncated fragments are also observed to bind TMR to some extend as seen previously *(14)* Bottom: Coomassie-stained gel from above. In both images, red arrows indicate HaloTag bands. (**D**) Same as main text Fig. 3C now showing all peptides with high quality mass spectra and replicate charge states where present. **(E)** Same as main text Fig. 3D now showing all peptides with high quality mass spectra and replicate charge states where present. **(F)** Same as main text Fig. 3C for a replicate HaloTag synchronized translation reaction. As in our first replicate, peptides from across the protein do not begin showing native protection until after translation is complete, and we observe protection in three similar stages, namely 1) rapid folding of C-terminal most helices, 2) folding of C-terminal regions of the core, with limited involvement from the N-terminus, and 3) folding of the majority of N-terminus and lid. We note that translation occurred at a slightly

slower and yielded slightly reduced peptide coverage than the initial replicate, particularly near the C-terminus. **(G)** Same as main text Fig. 3C for replicate HaloTag synchronized translation reaction. As in the initial replicate, we observe subtle albeit significant burst-phase protection at various N-terminal peptides. We note that for peptide 91-99, the maximally deuterated control for this replicate gave poor spectra, and we thus used the spectra from the initial replicate instead. We validated this substitution by verifying that the maximally deuterated for this peptide from a third experiment (namely derived from stalled nascent chains) showed nearly identical deuteration. (**H)** To confirm peptides acquire protection in a reproducible sequence across replicates, we compare, for each peptide, the fractional population associated with the natively protected mode at 30 mins in both replicates. A high Spearman correlation coefficient and low p value (shown on the plot) indicate a high degree of rerpodcubility. This plot only includes peptides with high quality fits in both replicates. **(I)** Same as (H) now showing *all* peptides common to both replicates.

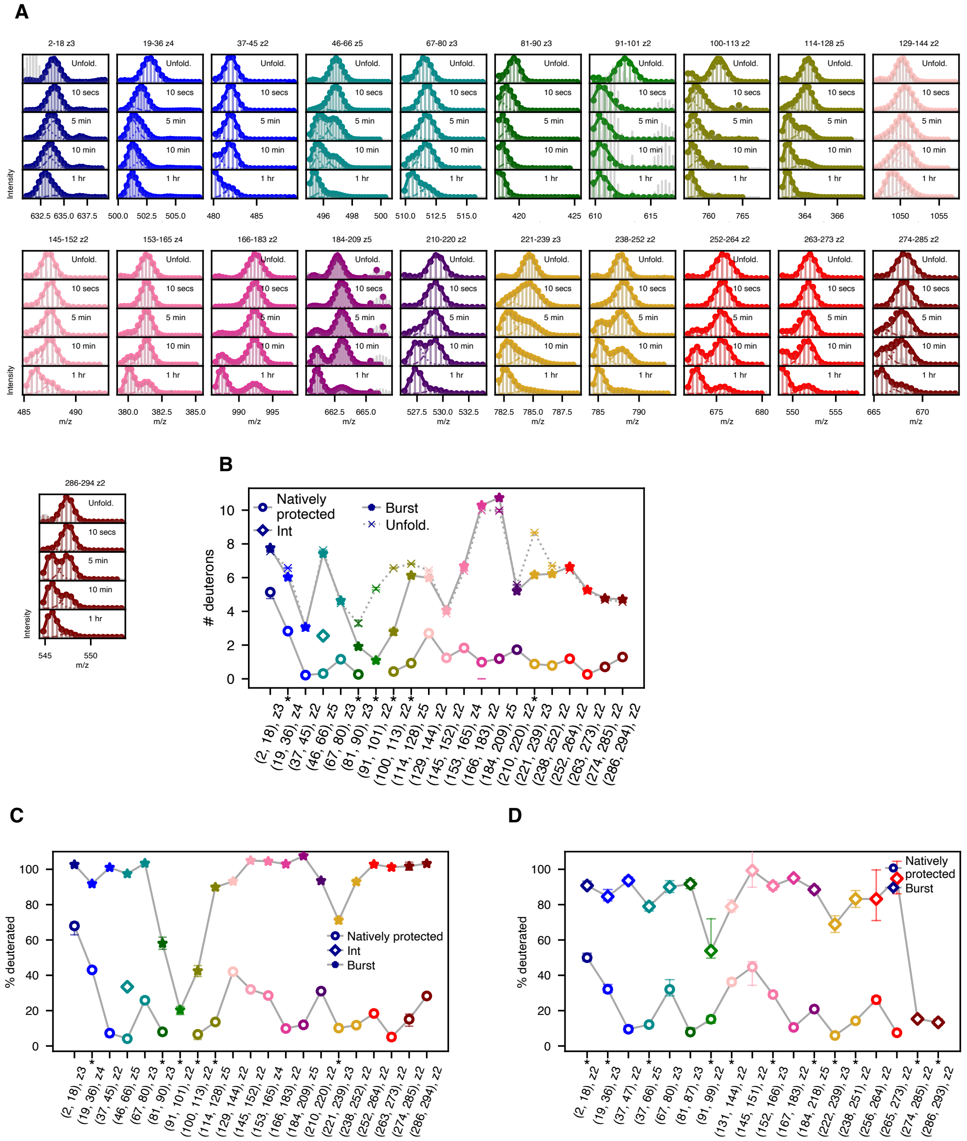

Fig. S8: HaloTag refolding from denaturant + pulse-labeling HDX-MS
(A) Mass spectra for all HaloTag peptides derived from unfolded control (top row) and all timepoints during refolding from denaturant, as in main text Fig. 2B. Data was obtained from reference*(15)* and reanalyzed using the same global fitting approach applied to co-translational expeiments as described in Materials and Methods . **(B)** Number of deuterons associated with natively protected mode (circles), intermediately protected mode where present (stars), burst-phase mode (the deprotected mode observed early in translation, diamonds) and unfolded state (Xs) for each peptide. Error bars represent 95% confidence intervals from bootstrapping and asterisks denote peptides exhibiting statistically significant burst-phase protection (see Methods). (**C**) Same as (B) with all # of deuton values expressed as a percentage relative to the respective values in the unfolded state. (**D**) Same as main text Fig. 3D, which shows deuteration of native

and burst modes in active translation experiment, with all # of deuton values expressed as a percentage relative to the respective values in the unfolded state. We note that the pattern of burst phase protection differs from that observed in the refolding experiment, with generally higher protection seen across the N terminus. This strongly suggests that the burst-phase intermediate in co-translational folding differs from that observed in refolding.

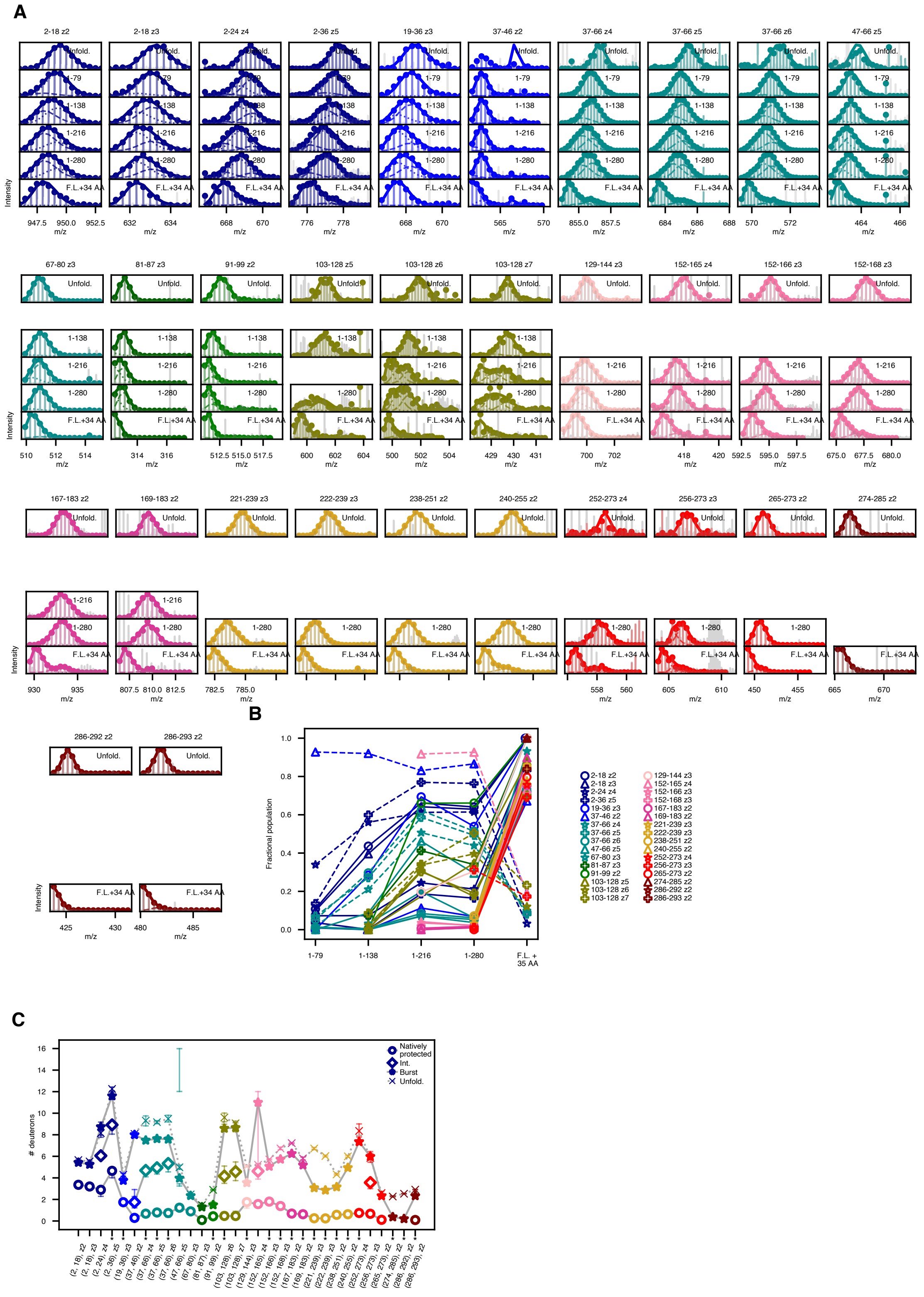

Fig. S9: HaloTag stalled ribosomal nascent chains (RNCs) + pulse-labeling HDX-MS
(A) Mass spectra for all HaloTag RNC peptides derived from unfolded control (top row) and all translation lengths, as in main text Fig. 4B. Empty panels reflect lack of peptide signal, either

because the peptide is yet synthesized at that length or due to stochastic variabilities in mass-spectrometric detection for low-abundance peptides. **(B)** Same as main text Fig. 4E, now showing all peptides with high quality mass spectra and replicate charge states where present. **(C)** Number of deuterons associated with natively protected mode (circles), intermediately protected mode where present (stars), burst-phase mode (the deprotected mode observed early in translation, diamonds) and unfolded state (Xs) for each peptide. Unfolded deuteration are corrected for 10 second labeling (see Materials and Methods). Error bars represent 95% confidence intervals from bootstrapping and asterisks denote peptides exhibiting statistically significant protection, relative to the maximally deuterated control corrected for 10 sec labeling, in the deprotected mode (see Materials and Methods).

References

1. Amiri, H.; Van Patten, W. J.; Rexroad, G.; Desai, V. P.; Sterwerf, B. A.; Lancaster, L.; et al. The ribosome derives the energy to translocate and unwind mRNA from EF-G binding. *Nature Communications 2025 17:1* **2025**, *17*(1), 121-. doi:10.1038/s41467-025-66812-7.
2. Cassaignau, A. M. E.; Launay, H. M. M.; Karyadi, M. E.; Wang, X.; Waudby, C. A.; Deckert, A.; et al. A strategy for co-translational folding studies of ribosome-bound nascent chain complexes using NMR spectroscopy. *Nature Protocols 2016 11:8* **2016**, *11*(8), 1492–1507. doi:10.1038/nprot.2016.101.
3. Bai, Y.; Milne, J. S.; Mayne, L.; Englander, S. W. Primary Structure Effects on Peptide Group Hydrogen Exchange. *Proteins* **1993**, *17*(1), 75. doi:10.1002/PROT.340170110.
4. Tuttle, L. M.; James, E. I.; Georgescauld, F.; Wales, T. E.; Weis, D. D.; Engen, J. R.; et al. Rigorous Analysis of Multimodal HDX-MS Spectra. *Journal of the American Society for Mass Spectrometry* **2025**, *36*(2), 416–423. doi:10.1021/JASMS.4C00471.
5. Sanders, R.; Loftus, D.; Kim, P. S.; Baldwin, R. L. *Effects of Denaturants on Amide Proton Exchange Rates: A Test for Structure in Protein Fragments and Folding Intermediatest*; 1986; Vol. 25.
6. Roeselová, A.; Pajak, A.; Wales, T. E.; Pellowe, G. A.; Kjær, S.; Engen, J. R.; et al. Hydrogen/deuterium exchange mass spectrometry analysis of ribosome-nascent chain complexes to study protein biogenesis at the peptide level. *Nature Protocols* **2026**. doi:10.1038/s41596-025-01279-w.
7. Shimizu, Y.; Kanamori, T.; Ueda, T. Protein synthesis by pure translation systems. *Methods* **2005**, *36*(3), 299–304. doi:10.1016/J.YMETH.2005.04.006.
8. Bitran, A.; Jacobs, W. M.; Shakhnovich, E. Validation of DBFOLD: An efficient algorithm for computing folding pathways of complex proteins. *PLoS Computational Biology* **2020**, *16*(11), e1008323. doi:10.1371/journal.pcbi.1008323.
9. Bitran, A.; Jacobs, W. M.; Zhai, X.; Shakhnovich, E. Cotranslational folding allows misfolding-prone proteins to circumvent deep kinetic traps. *Proceedings of the National Academy of Sciences of the United States of America* **2020**, *117*(3), 1485–1495. doi:10.1073/pnas.1913207117.
10. Beitlich, T.; Lorenz, T.; Reinstein, J. Folding properties of cytosine monophosphate kinase from E. coli indicate stabilization through an additional insert in the NMP binding domain. *PLoS ONE* **2013**, *8*(10). doi:10.1371/journal.pone.0078384.

11. Shirts, M. R.; Chodera, J. D. Statistically optimal analysis of samples from multiple equilibrium states. *The Journal of chemical physics* **2008**, *129*(12), 124105. doi:10.1063/1.2978177.
12. Wang, S.; Bitran, A.; Samatova, E.; Shakhnovich, E. I.; Rodnina, M. V. Cotranslational protein folding through non-native structural intermediates. *Science Advances* **2025**, *11*(36), 1–11. doi:10.1126/sciadv.ady2211.
13. Wintrode, P. L.; Rojsajjakul, T.; Vadrevu, R.; Matthews, C. R.; Smith, D. L. An obligatory intermediate controls the folding of the α-subunit of tryptophan synthase, a TIM barrel protein. *Journal of Molecular Biology* **2005**, *347*(5), 911–919. doi:10.1016/j.jmb.2005.01.064.
14. Samelson, A. J.; Bolin, E.; Costello, S. M.; Sharma, A. K.; O'Brien, E. P.; Marqusee, S. Kinetic and structural comparison of a protein's cotranslational folding and refolding pathways. *Science Advances* **2018**, *4*(5). doi:10.1126/sciadv.aas9098.
15. Dall, N. R.; Mendonça, Carolina A.T.F.; Torres Vera, H. L.; Marqusee, S. The importance of the location of the N-terminus in successful protein folding in vivo and in vitro. *Proceedings of the National Academy of Sciences* **2024**, *121*(34), e2321999121.
